# Phylogeny and evolution of the genus *Cervus* (Cervidae, Mammalia) as revealed by complete mitochondrial genomes

**DOI:** 10.1038/s41598-022-20763-x

**Published:** 2022-09-30

**Authors:** Paweł Mackiewicz, Maciej Matosiuk, Magdalena Świsłocka, Frank E. Zachos, Ghaiet M. Hajji, Alexander P. Saveljev, Ivan V. Seryodkin, Tarlan Farahvash, Hamid Reza Rezaei, Rasoul Vaez Torshizi, Stefano Mattioli, Mirosław Ratkiewicz

**Affiliations:** 1 Faculty of Biotechnology, University of Wrocław, Fryderyka Joliot-Curie 14a, 50-383 Wrocław, Poland; 2 Faculty of Biology, University of Bialystok, Ciołkowskiego 1J, 15-245 Białystok, Poland; 3 Mammal Collection, Natural History Museum Vienna, Burgring 7, 1010 Vienna, Austria; 4 Department of Evolutionary Biology, University of Vienna, Vienna, Austria; 5 Department of Genetics, University of the Free State, Bloemfontein, South Africa; 6 Zoological Institute, Christian-Albrechts-Universität, Am Botanischen Garten 1-9, 24118 Kiel, Germany; 7 Department of Animal Ecology, Russian Research Institute of Game Management and Fur Farming, Preobrazhenskaya 79, 610000 Kirov, Russia; 8 Laboratory of Ecology and Conservation of Animals, Pacific Geographical Institute of Far East Branch of Russian Academy of Sciences, Radio 7, 690041 Vladivostok, Russia; 9 Department of Animal Science, Faculty of Animal Science and Veterinary, Islamic Azad University, Shabestar Brunch, Shabestar, Iran; 10 Department of Environmental Science, Faculty of Fisheries and Environmental Science, Gorgan University of Agricultural Sciences and Natural Resources, Gorgan, Iran; 11 Department of Animal Science, Faculty of Agriculture, Tarbiat Modares University, Tehran, Iran; 12 Research Unit of Behavioural Ecology, Ethology and Wildlife Management, Department of Life Sciences, University of Siena, Siena, Italy

**Keywords:** Molecular evolution, Phylogenetics

## Abstract

Mitochondrial DNA (mtDNA) lineages are recognized as important components of intra- and interspecific biodiversity, and allow to reveal colonization routes and phylogeographic structure of many taxa. Among these is the genus *Cervus* that is widely distributed across the Holarctic. We obtained sequences of complete mitochondrial genomes from 13 *Cervus* taxa and included them in global phylogenetic analyses of 71 Cervinae mitogenomes. The well-resolved phylogenetic trees confirmed *Cervus* to be monophyletic. Molecular dating based on several fossil calibration points revealed that *ca*. 2.6 Mya two main mitochondrial lineages of *Cervus* separated in Central Asia, the Western (including *C. hanglu* and *C. elaphus*) and the Eastern (comprising *C. albirostris*, *C. canadensis* and *C. nippon*). We also observed convergent changes in the composition of some mitochondrial genes in *C. hanglu* of the Western lineage and representatives of the Eastern lineage. Several subspecies of *C. nippon* and *C. hanglu* have accumulated a large portion of deleterious substitutions in their mitochondrial protein-coding genes, probably due to drift in the wake of decreasing population size. In contrast to previous studies, we found that the relic haplogroup B of *C. elaphus* was sister to all other red deer lineages and that the Middle-Eastern haplogroup E shared a common ancestor with the Balkan haplogroup C. Comparison of the mtDNA phylogenetic tree with a published nuclear genome tree may imply ancient introgressions of mtDNA between different *Cervus* species as well as from the common ancestor of South Asian deer, *Rusa timorensis* and *R. unicolor*, to the *Cervus* clade.

## Introduction

Due to sufficient variation, the lack of recombination and simple maternal inheritance, mitochondrial DNA (mtDNA) has become very useful in various phylogenetic and evolutionary studies. These analyses often found clear phylogeographic patterns enabling the inference of evolutionary and biogeographic histories for many mammals^1–9^. Among mammalian taxa that show complex phylogenetic and phylogeographic mtDNA patterns is the genus *Cervus*, which underwent a successful radiation together with other ruminants^10^.

**Figure 1:**
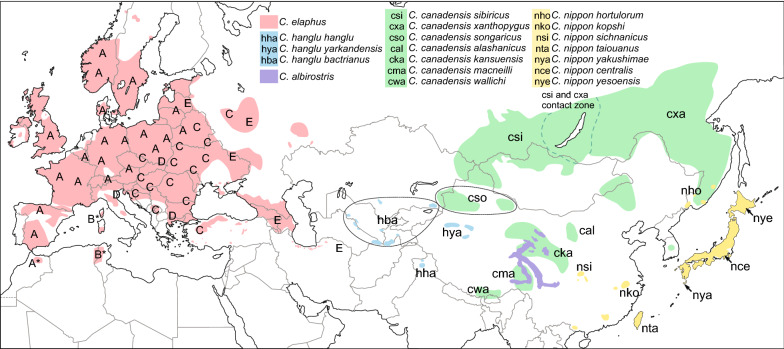
Contemporary distribution of *Cervus* species and subspecies. Haplogroups (rather than subspecies) of *C. elaphus* (Western red deer) are marked as letters A-E. Deer haplogroups introduced to selected regions are marked with an asterisk*. The distribution ranges were compiled from several sources^2,104–119^. Figure was created in CorelDRAW Graphics Suite 11 (https://www.coreldraw.com/en/) based on OpenStreetMap (URL https://www.openstreetmap.org/copyright; ODbL licence 1.0 https://opendatacommons.org/licenses/odbl/1-0/ by OSMF).

Different species and subspecies of *Cervus* occupy a variety of habitats so that the range of *Cervus* is the largest among all cervids^11^. However, many similarities in the morphology make the taxonomic classification within the Cervinae difficult, especially within the highly plastic genus *Cervus*. The genus *Cervus* has a wide range across Eurasia and North America and is usually taken to include the following species: Western red deer (*Cervus elaphus*), Central Asian red deer (*C. hanglu*), wapiti (*C. canadensis*), sika (*C. nippon*) and Thorold’s or white-lipped deer (*C. albirostris*)^12^ (Fig. 1). *C. elaphus* and *C. hanglu* are also described as the Western mtDNA lineage or clade, whereas wapiti and sika deer are classified to the Eastern mtDNA lineage or clade^13,14^. The position of *C. albirostris* has been ambiguous (see Fig. 1).

*Cervus elaphus* is one of the best-studied mammal species with respect to intraspecific mtDNA phylogeny and phylogeographic history^2,3,13,15–21^. It plays a major role in shaping forest vegetation in Europe^22^ and is an important game animal^23^. Five extant mtDNA haplogroups have been identified in contemporary *C. elaphus*: Western (A) in western and central Europe; Eastern (C) in central-eastern and south-eastern Europe; Italian/Mediterranean (B) native, but extinct, to the Italian mainland, introduced on the Tyrrhenian islands Sardinia and Corsica and in northern Africa; Mesola (D) in the Po delta region of Italy and south-eastern Poland; and Caucasian (sometimes called Caspian or maral) (E) in the Caucasus and south-western Asia^2,15,18,21,24^ (Fig. 1). Additionally, several extinct lineages based on fossil samples have been identified^2,3,13,17^.

Several analyses provided inconsistent results on the phylogeny of *Cervus*. For example, some studies indicated the monophyly of the genus *Cervus*^15,25,26^ but in others, different *Cervus* taxa were separated by representatives of *Rusa*^13,27–29^. *C. hanglu* was usually assigned to the Western clade^3,13–16,28,30^ but other analyses grouped this species with the Eastern clade^2^. Most analyses showed that *C. albirostris* is a member of the Eastern lineage^13,26,28,31^ but other authors found it closer to the Western lineage^32^.

Potential reasons for such disagreements are the use of a single molecular marker and/or biased taxon sampling. The majority of these studies were based on short mtDNA control region or cytochrome b sequences only, which may produce not fully resolved phylogenies. This also hampers taxonomic decisions or in-depth studies of evolutionary processes and estimations of divergence times within and among species of *Cervus*. For example, the split of the Western and Eastern lineages varies among studies from 374 kya or 1.37 Mya^2^ to 6 Mya^14^.

Thus, to arrive at a better-resolved and dated phylogeny of *Cervus* species and to draw conclusions about mitogenome evolution in deer we analysed 13 newly sequenced mitochondrial genomes representing *C. elaphus* and *C. canadensis* and enlarged our dataset with mitogenomes of other deer species available in GenBank. We aim to make maximal use of the phylogenetic signal carried by mtDNA and produce novel insights from protein coding genes.

## Results

### Comparison of deer mitogenomes

The compared mitochondrial genomes of *Cervus* show an organization typical of other mammals but are variable in size, which is associated mainly with the different length of the control region. Eastern lineage mitogenomes (median: 16,432; min–max: 16,353–16,663) are significantly longer (p = 3.6E−06) than Western lineage mitogenomes (median: 16,352.5; min–max: 16,350–16,357). Mitogenome length of *Rusa timorensis* and *R. unicolor* (median: 16,436.5; min–max: 16,434–16,477) is similar to the Eastern lineage (p = 0.49) but statistically longer than for the Western lineage (p = 6. 3E−04).

**Figure 2:**
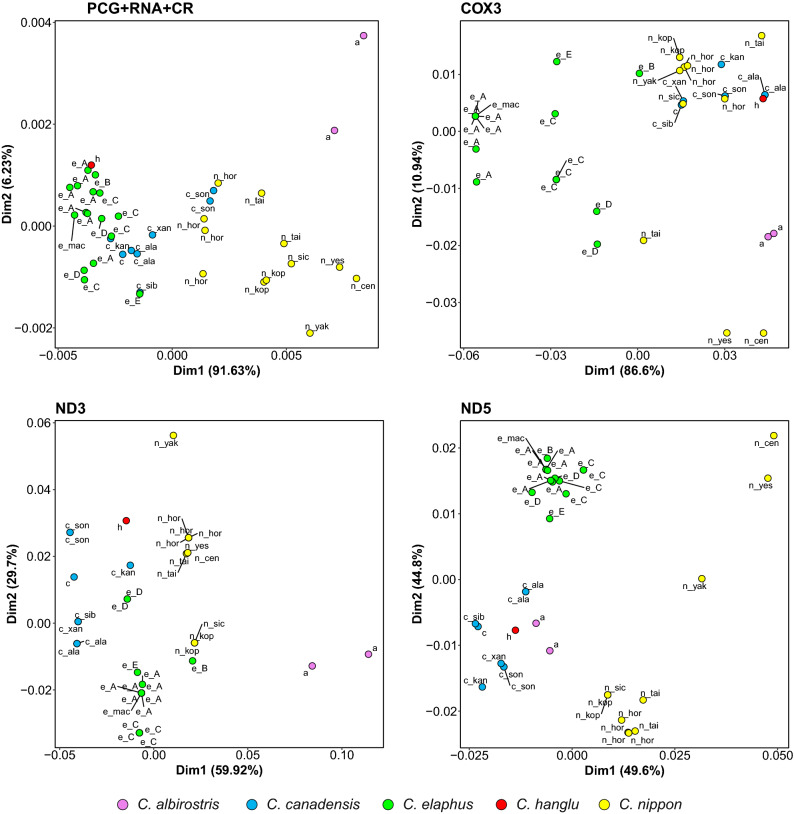
Correspondence analysis plots obtained for the nucleotide composition of 13 protein-coding genes (PCG), RNA genes and control region (CR) as well as selected PCG from various *Cervus* taxa: a—*C. albirostris*; c—*C. canadensis*; c_ala—*C. canadensis alashanicus*; c_kan—*C. canadensis kansuensis*; c_sib—*C. canadensis sibiricus*; c_son—*C. canadensis songaricus*; c_xan—*C. canadensis xanthopygus*; e_A—*C. elaphus* haplogroup A; e_B—*C. elaphus* haplogroup B; e_C—*C. elaphus* haplogroup C; e_D—*C. elaphus* haplogroup D; e_E—*C. elaphus* haplogroup E; e_mac—*C. elaphus macneilli* (probably mislabelled, see caption to Fig. 5); h—*C. hanglu yarkandensis*; n_cen—*C. nippon centralis*; n_hor—*C. nippon hortulorum*; n_kop—*C. nippon kopschi*; n_sic—*C. nippon sichuanicus*; n_tai—*C. nippon taiouanus*; n_yak—*C. nippon yakushimae*; n_yes—*C. nippon yesoensis.* The plots for other genes and control region are shown in Fig. S1.

Despite the conserved genome organization, we noticed differences in nucleotide composition of these mitogenomes and individual genes, usually in their third codon position. In the correspondence analysis plot, the mitogenomes from the Western lineage were clearly separated from *C. nippon* mitogenomes (Figs. 2, S1). *C. canadensis* mitogenomes occupied an intermediate position, whereas *C. albirostris* was represented by distant points. Generally, mitogenomes from the Western lineage and most *C. canadensis* have more thymine and adenine. In turn, *C. nippon* mitogenomes are richer in cytosine and *C. albirostris* also in guanine. *C. albirostris* shows differences in composition from other mitogenomes in the *atp6*, *nd2*, *nd3*, *nd4*, *nd6* and 12S rRNA genes (Figs. 2, S1). Likewise, *C. hanglu* also deviates from others in the composition of the *cox2*, *nd6* and 16S rRNA genes (Fig. S1). We also noticed that mitogenomes of Japanese deer, i.e. *C. nippon yakushimae*, *C. n. centralis* and *C. n. yesoensis*, showed deviating nucleotide compositions in *atp6*, *atp8*, *cox2* and *nd5* (Fig. S1). *C. nippon yakushimae* occupied a distant position in the plot also for the *cytb*, *nd2*, *nd3*, *nd4* and *nd4L* genes, whereas the two other subspecies showed a nucleotide bias in *cox3* and the control region (Figs. 2, S1). Similarly, the genes *atp8*, *cox1*, *cox2*, *cox3*, *nd1*, *nd2*, and *nd4L* of *C. elaphus barbarus* differed in their nucleotide compositions from other red deer haplogroups (Figs. 2, S1).

*C. hanglu* showed contradictory results. In line with its phylogenetic position as sister to *C. elaphus* (see below), the nucleotide compositions of most studied sequences are similar in these two species, but for some other mitochondrial genes, *C. hanglu* was closer to representatives of the Eastern clade: *C. nippon* and *C. canadensis* in the case of *cox3*, *cytb*, *nd1* and *nd3* as well as *C. albirostris* and *C. canadensis* in the *cox1*, *nd5* and tRNA genes (Figs. 2, S1).

**Figure 3:**
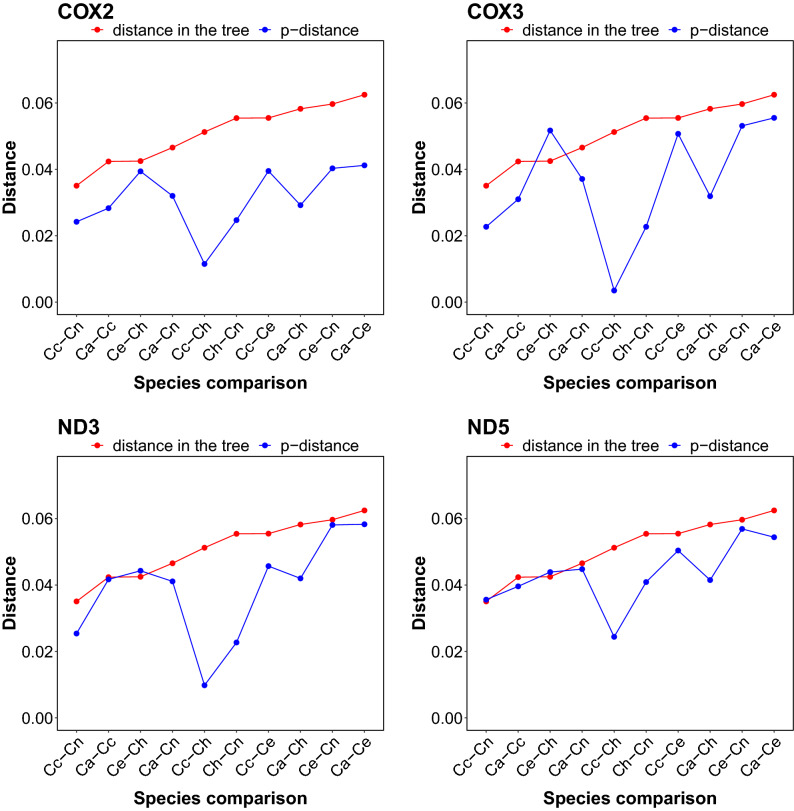
Comparison of p-distances for all pairs of *Cervus* species with the distances obtained from the MrBayes phylogenetic tree for selected protein-coding genes. The species pairs were arranged according to the tree distance. Ca—*C. albirostris*; Cc—*C. canadensis*; Ce—*C. elaphus*; Ch—*C. hanglu*; Cn—*C. nippon.* The plots for other genes and the control region are shown in Fig. S2.

**Figure 4:**
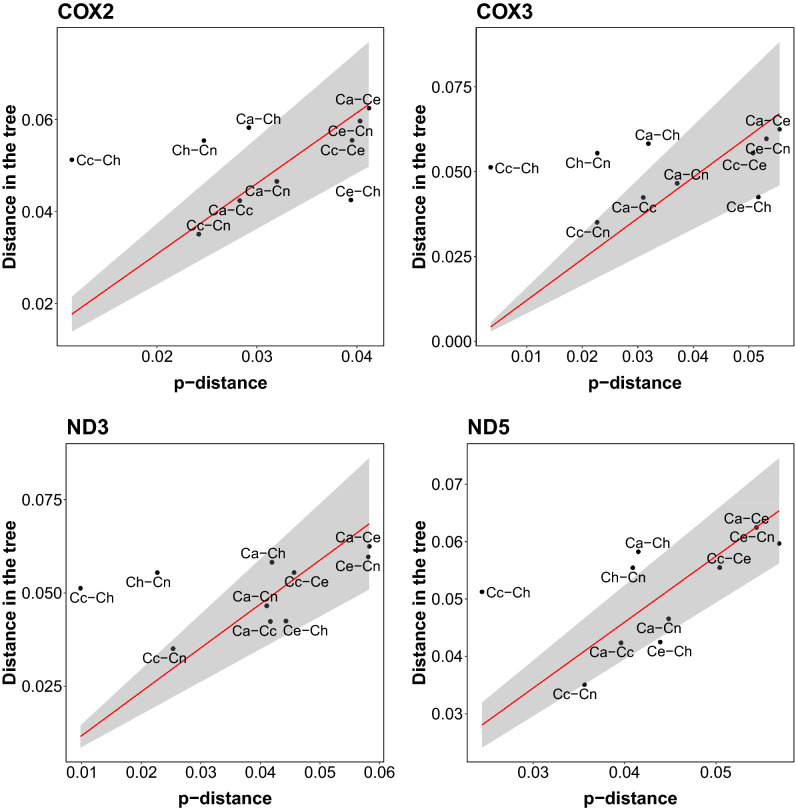
Relationships between the distances in the MrBayes phylogenetic tree and p-distances for all pairs of *Cervus* species and selected protein-coding genes. A regression line (red line) with 95% confidence interval (grey area) is shown. Ca—*C. albirostris*; Cc—*C. canadensis*; Ce—*C. elaphus*; Ch—*C. hanglu*; Cn—*C. nippon.* The plots for other genes and the control region are shown in Fig. S3.

The similarity in the nucleotide composition is reflected in substantially smaller p-distances (the proportion of different sites) than expected between individual genes from *C. hanglu* and the Eastern lineage representatives in comparisons with the distances obtained from the MrBayes phylogenetic tree (Figs. 3, S2). The p-distance is substantially smaller for the *cox1*, *cox2*, *cox3*, *nd3, nd4, nd4L* and *nd5* genes, especially between *C. hanglu* and *C. canadensis*. Similar findings are visible in plots showing relationships between the distance in the tree and the p-distance (Figs. 4, S3). Points representing distances between *C. hanglu* and the Eastern lineage deer are often positioned on the left of a regression line due to the smaller p-distance. Besides the protein-coding genes, the large shift is also visible for tRNA genes (Fig. S3).

### Phylogenetic analyses of mitogenome sequences

**Figure 5:**
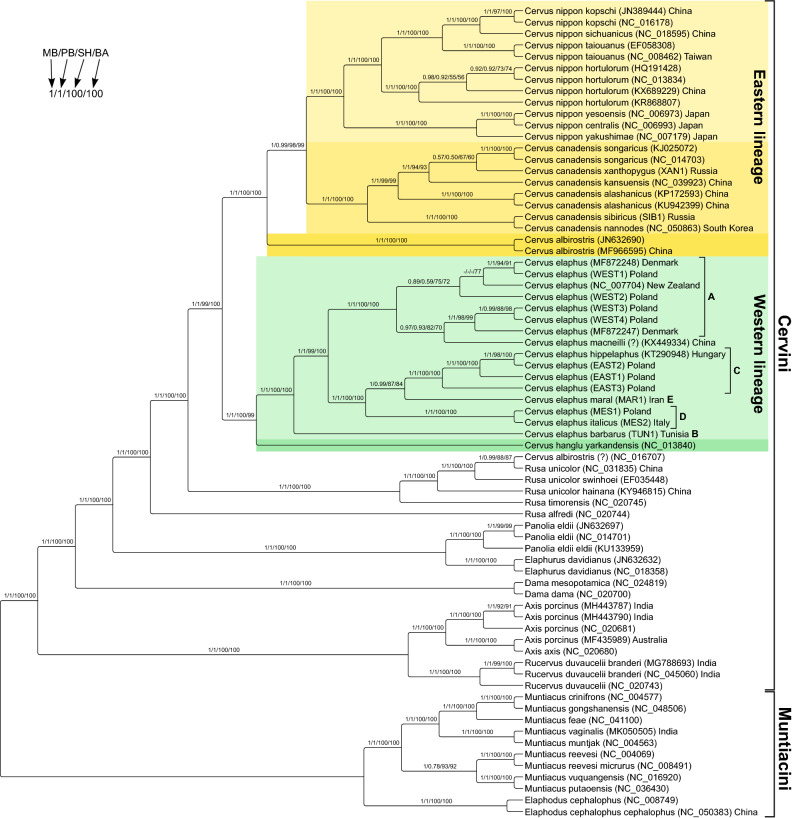
The consensus of trees obtained in three approaches for the alignment of control region and genes coding for 13 proteins, two rRNAs and 22 tRNAs with a total length of 16,306 bp from Cervinae (Muntiacini + Cervini). Numbers at nodes, in the following order, correspond to: posterior probabilities estimated in MrBayes (MB) and PhyloBayes (PB) as well as support values obtained by the approximate likelihood ratio test based on a Shimodaira-Hasegawa-like procedure (SH) and the bootstrap method (BA) calculated in IQ-TREE. Values of the posterior probabilities and bootstrap percentages lower than 0.50 and 50%, respectively, were indicated by a dash "–". Letters (A–E) next to *Cervus elaphus* clades refer to intraspecific phylogeographic haplogroups. *C. albirostris* (NC_016707) is likely a contamination or incorrect labelling because its sequence is more similar to *Rusa unicolor* than other samples of *R. unicolor* to each other, whereas two other sequences assigned to *C. albirostris* are clearly separated from NC_016707 and are grouped with *Cervus* sequences with high support. The same holds for *C. elaphus macneilli* (KX449334), which is a subspecies of the wapiti native to Western China and typically called *C. canadensis macneilli*. Therefore, this sample could also be mislabelled or contaminated. Alternatively, these two specimens can be a result of mitochondrial introgression. *C. canadensis nannodes* sample (NC_050863) was translocated or imported for farming to South Korea^120^ from its native site in California in North America. The specimen *C. elaphus* (NC_007704) from New Zealand is also an introduction by humans.

All three phylogenetic approaches produced the same very well-resolved tree topology (Fig. 5). Out of 68 internal nodes, 45 obtained the maximal support with all these methods, 57 with at least two methods and 62 with at least one method. Only four nodes of closely related mitogenomes were weakly supported. Representatives of Muntiacini and Cervini are clearly separated into two separate clades. Within Cervini, *Rucervus*, *Axis*, *Dama*, *Elaphurus*, *Panolia* and *Cervus* are all monophyletic (Fig. 5). *Rucervus* and *Axis* are sister taxa, as are *Elaphurus* and *Panolia*. The genus *Rusa* is non-monophyletic in our tree due to the position of *R. alfredi* as sister to the other *Rusa* species and *Cervus* combined.

The genus *Cervus* is clearly separated into the Western and Eastern mtDNA lineages, which are both monophyletic and highly supported (Fig. 5). The Western lineage comprises *C. hanglu* and *C. elaphus*. Within the latter, *C. elaphus barbarus* (haplogroup B) is sister to all other *C. elaphus* samples grouped into two well-supported clades. The first comprises samples of haplogroup A from more western localities in Europe. The other haplotypes are clustered in the second group, in which the Mesola lineage (haplogroup D) is sister to a clade comprising the East-European lineage (haplogroup C) and the Middle-Eastern lineage (haplogroup E).

In the clade of eastern species, *C. albirostris* is sister to *C. canadensis* and *C. nippon* combined. The two new wapiti mitogenomes from *C. c. sibiricus* and *C. c. xanthopygus* are clustered with high support with *C. c. nannodes* and poorly with *C. c. songaricus*, respectively (Fig. 5). The sika group consists of two subgroups, Japanese deer and samples from Taiwan and mainland Eastern Asia.

### Estimating divergence times within Cervini

Cervini started its evolution after separation from Muntiacini *ca.* 10.4 Mya (Fig. S4). Later, the next split separated the *Axis* + *Rucervus* lineage from the remaining Cervini *ca.* 7.5 Mya. At *ca.* 5.7 Mya, *Axis* and *Rucervus* split as did the *Dama* clade and the remaining Cervini. About 4.4 Mya, *Panolia* + *Elaphurus* separated from the remaining taxa, and approximately one million years later these two genera split. *Rusa alfredi* and *R. timorensis* + *R. unicolor* separated from their respective sister taxa at *ca.* 3.6 Mya and *ca.* 2.9 Mya, respectively.

**Figure 6:**
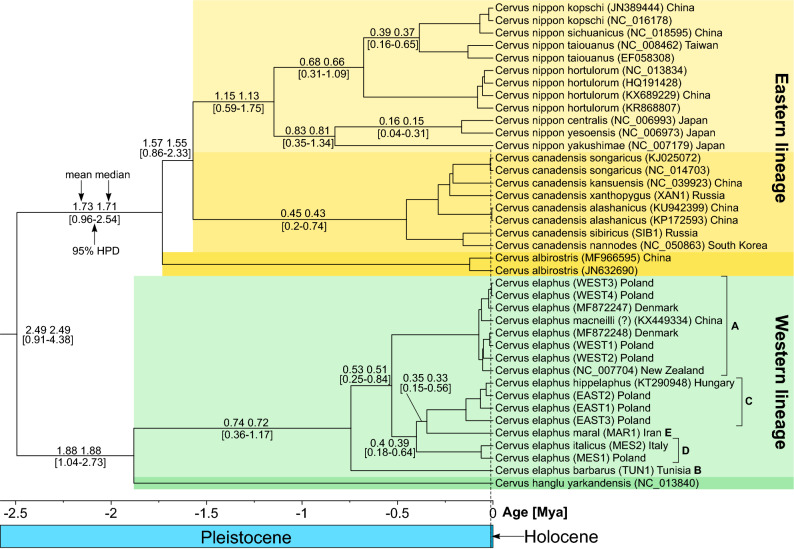
The chronogram for *Cervus*. Mean and median as well as 95% Highest Posterior Density (HPD) of individual node ages are shown at selected branches. Letters (A–E) next to *Cervus elaphus* clades refer to intraspecific phylogeographic haplogroups.

The deepest split within *Cervus* into the Western and Eastern lineages was estimated at *ca.* 2.5 Mya. The split between *C. hanglu* and *C. elaphus* as well as between *C. albirostris* and *C. canadensis* + *C. nippon* occurred at *ca.* 1.9 and 1.7 Mya, respectively (Fig. 6). Finally, *C. canadensis* and *C. nippon* separated *ca.* 1.6 Mya. The Japanese lineage of *C. nippon* evolved from the mainland deer *ca.* 1.1 Mya, whereas the northern and central Japanese subspecies split from the southern one *ca.* 0.8 Mya. *C. elaphus* haplogroups identified within the Western lineage have an age from 0.7 to 0.3 Mya. Our datings are in agreement with the fossil deer *C. magnus* (2.25–1.26 Mya), which is considered to be ancestral to or close to the ancestor of *C. canadensis* after the split of the Western and Eastern lineages^33^. In turn, *C. nestii*, used by us as a calibration point (2.1–1.95 Mya), is most likely associated with the Western lineage^34,35^. The age of *C. grayi* (1.3–1.25 Mya) fits the timing of the *C. nippon* lineage to which this fossil is ascribed^36^. *C. elaphus acoronatus* is the oldest representative of red deer^35^, and its age (1–0.8 Mya) is in agreement with our molecular dating for the oldest branch of the *C. elaphus* clade.

The results of molecular dating were compared with the δ^18^O curve ^37^, which is used as a climate proxy (Fig. S4). The comparison indicates that the emergence of many clades within *Cervus* corresponds well to the increase in climate oscillations in the Pleistocene since its beginning *ca.* 2.6 Mya.

### Analysis of substitutions in mitochondrial protein-coding genes

**Figure 7:**
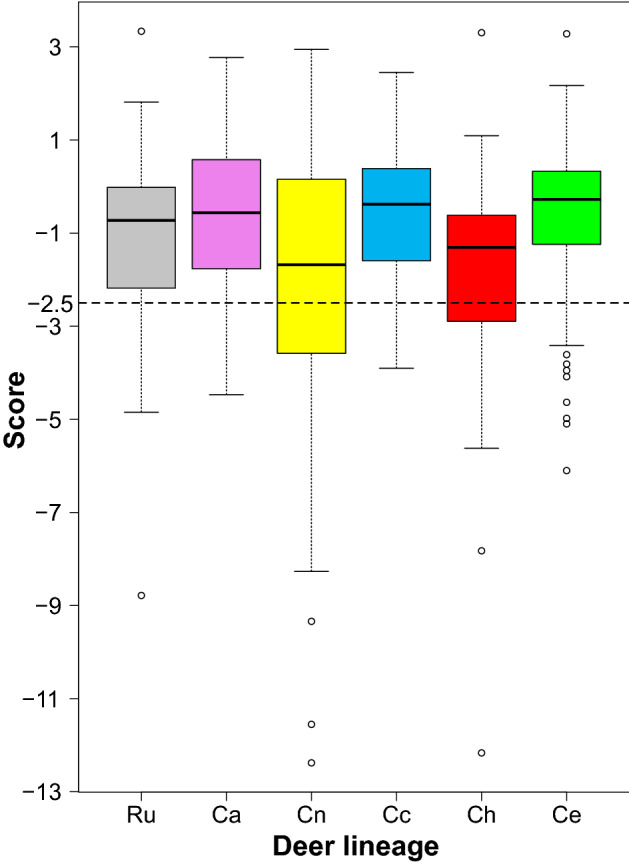
Box-plots of Provean scores in various deer species: Ru—*R. unicolor* and *timorensis*; Ca—*C. albirostris*; Cn—*C. nippon*; Cc—*C. canadensis*; Ch—*C. hanglu*; Ce—*C. elaphus.* The scores quantify negative effects of amino acid substitutions on proteins. The thick line indicates the median, the box shows quartile ranges, and the whiskers denote the range without outliers shown as circles. The dashed line shows the threshold assuming deleterious (< − 2.5) and neutral (> − 2.5) mutations^94^.

We found that amino acid sequences of mitochondrial genes accumulated variable numbers of deleterious mutations in different deer lineages (Table S1). The largest proportion of deleterious mutations was accumulated in the lineages of *C. nippon* (0.37) and *C. hanglu* (0.33), whereas the smallest was found in *C. albirostris* and *C. canadensis* (0.12 and 0.13, respectively). This proportion in *C. nippon* was statistically significantly greater (p = 0.023) than in *C. albirostris*, *C. canadensis* and *C. elaphus*. We also compared the distribution of scores describing the deleterious effect (Fig. 7). The more negative the Provean score is, the more negative the impact on the biological function of a protein by a given substitution. *C. nippon* and *C. hanglu* were characterized by a larger number of substitutions with more negative effect and demonstrated significantly lower scores in pairwise comparisons with all other deer lineages (p < 0.041 and p < 0.019, respectively).

We checked if the individual protein-coding genes were subjected to positive selection in *Cervus* evolution. A significant (p < 0.044) excess of nonsynonymous over synonymous nucleotide substitutions was demonstrated by four approaches (original and modified Nei-Gojobori methods with Jukes-Cantor and proportion models) for *atp8* in the comparisons of: *C. canadensis xanthopygus* (XAN1) with *C. canadensis alashanicus* (KU942399 and KP172593) as well as *C. elaphus barbarus* (TUN1) with *C. elaphus hippelaphus* (KT290948), *C. elaphus* (EAST2) and *C. elaphus maral* (MAR1). Significant (p < 0.045) positive selection was also yielded by six approaches, the four mentioned above as well as Liu-Wu-Luo^38^ and Pamilo-Bianchi-Li^39^ methods, for *cox1* between two Japanese deer, *C. nippon yesoensis* (NC_006973) and *C. nippon centralis* (NC_006993). When the protein-coding genes were agglomerated into groups of *Cervus* species, significant (p < 0.029) positive selection was still observed for *atp8* in *C. elaphus* by the four approaches.

Tajima's and Fu’s neutrality tests calculated for nucleotide sequences of protein-coding genes did not provide very low or very high values of their respective statistics, but we observed the most negative values for the Western deer species (D = − 1.3) and *C. elaphus* (D = − 0.6) (Table S2). For the Eastern deer species, the statistics were much closer to zero (from − 0.3 to 0.1) except Fs for *C. canadensis* and *C. nippon*. In these cases, this parameter reached or exceeded 1. The highest positive D and Fs values were found for *C. nippon* from mainland Asia, 0.97 and 1.24, respectively.

## Discussion

### Evolution of deer mitogenomes

The differences between cervid mitogenomes have accumulated over time since their divergence. The mitogenome sequences of the Western deer lineage became shorter during their evolution, whereas the Eastern lineage preserved a length similar to the sister taxa *Rusa timorensis* and *R. unicolor* and probably to the common ancestor. However, in some taxa, e.g. *C. canadensis alashanicus*, *C. albirostris*, *C. nippon yesoensis*, *C. nippon hortulorum* and *C. nippon centralis*, the mitogenome increased in length mainly due to insertions in the control region.

Deviating nucleotide compositions are present especially in the mitogenomes of *C. albirostris* and *C. hanglu* as well as *C. elaphus barbarus*. The nucleotide bias could be related to the isolation and separate evolution of these deer populations and perhaps a founder effect and genetic drift, which led to the accumulation of differences in the nucleotide composition when compared to their common and widespread relatives.

*C. hanglu* is included within the Western mtDNA lineage. Therefore, the similarity of selected genes from *C. hanglu* and the Eastern lineage deer in their composition could result from convergent evolution or inheritance from the common ancestor (ancestral polymorphism). To decide between these possibilities we repeated the correspondence analysis including the nucleotide composition of sequences inferred in the common ancestor of *Cervus*. A similar composition of such sequences to those from *C. hanglu* and the Eastern lineage deer would be indicative of the ancestral state. The analysis showed that the composition of *cox1* and *cytb* could have evolved convergently, whereas that of *cox3*, *nd1*, *nd3*, *nd5* and tRNA genes probably represents a plesiomorphic (ancestral) state (Fig. S5). Interestingly, the Central Asian geographic distribution of *C. hanglu* is closer to that of the Eastern lineage. Thus, it is not inconceivable that similar evolutionary pressures related to environmental and climatic conditions influenced the mitochondrial genes involved in energy production. The similar nucleotide composition of *C. hanglu* and Eastern lineage representatives is responsible for a decrease in p-distance between their mitochondrial sequences.

### Phylogenetic relationships within Cervini

Our trees are characterized by high statistical node support, and the phylogenetic relationships among the studied taxa corresponded to those from some other studies^26,31^. Our findings based on mtDNA suggest that *Rucervus eldii* should be moved into *Panolia eldii*^40^ based on mtDNA (albeit nuclear DNA seems to suggest otherwise, see^44^), which is grouped with *Elaphurus davidianus* and separated from *Rucervus duvaucelii* that is clustered with *Axis*. Similarly, *Przewalskium albirostris* (or *albirostre*) should be classified as *Cervus albirostris*^25,41^ because it is grouped with the other *Cervus* species. The phylogenetic position of *Elaphurus* has been debated in the context of its hybrid origin^28,29,42,43^. Its female parent was related to *Panolia/Rucervus eldii* as indicated by mtDNA trees, similar habitat and some morphological features, whereas the male parent could have been *Cervus*, e.g. *C. canadensis*, due to the same karyotype, several morphological and behavioural similarities as well as phylogenetic relationships in the trees based on nuclear genes, which are, however, not well-resolved. In fact, hybrids between *Elaphurus* and *C. elaphus* seem to be fertile^44^.

The genus *Rusa* is paraphyletic with respect to *Cervus* in our mtDNA tree, which has also been found by other authors^25–27,29,43^. However, the phylogenetic analyses involving nuclear data did not yield *R. unicolor*^45^ or *R. timorensis*^29^ as sister taxa to *Cervus*. To reconcile these results, one could assume ancestral polymorphisms or mitochondrial introgression from the lineage of *R. timorensis* and *R. unicolor* to the common ancestor of *Cervus* or in the opposite direction. Since *Cervus* and *Rusa* are related taxa, occasional crossbreedings between them could have been possible in the past. In agreement with that, a high probability of interspecific gene exchange was suggested between *C. albirostris* and *P. eldii*, *C. albirostris* and *R. unicolor* as well as *C. nippon* and *R. unicolor* based on nuclear genome studies^45^.

Our phylogenetic trees consistently yielded the monophyly of *Cervus*. This has also been found based on cytochrome b^15,25^ and more mitochondrial markers^26,31^, but those studies included a smaller number of taxa and/or provided weaker support. In other analyses, the monophyly was disrupted as *R. timorensis* and *R. unicolor* were grouped with the Eastern^13,27,28^ or Western^27^ lineage of *Cervus*. Our study including the largest number of species represented by complete mitogenomes clearly supports the monophyly of the genus *Cervus.*

### Phylogenetic relationships between species within the genus *Cervus*

Our phylogenetic analyses confirmed two well-supported clades of *Cervus* corresponding to the western and eastern part of its range. The Western lineage includes *C. elaphus* and *C. hanglu*. In the Eastern lineage, *C. albirostris* was sister to *C. canadensis* and *C. nippon* combined. The same, albeit less supported topology, was received by Ludt et al.^15^ based on cytochrome b, and *C. hanglu* was also found part of the Western lineage in the trees based on the control region and cytochrome b^3,13,14,16,28,30^. However, in a recent study based on a large number of cytochrome b sequences *C. hanglu* was sister to *C. elaphus, C. canadensis* and *C. nippon* combined^2^.

The assignment of *C. albirostris* to the Eastern lineage is also supported by other phylogenetic studies^13,26,28,31^ with one exception^32^. Moreover, *C. albirostris*, *C. canadensis* and *C. nippon* share morphological similarities in rump-patch colours and antler conformation but they were considered as convergent features^46,47^. The sister group relationship between *C. canadensis* and *C. nippon* for mtDNA is also an ubiquitous result across many studies^2,13,14,16,26–28,30,48,49^.

Contrary to the mitochondrial tree (Fig. 5), nuclear genome analyses^45^ showed a close relationship of *C. canadensis* and *C. hanglu*, which both combined were sister to *C. elaphus*. *C. nippon* was a sister taxon to these three, and *C. albirostris* was sister to all other *Cervus* species. To reconcile these conflicting phylogenies based on nuclear and mitochondrial genomes, one could hypothesise ancient introgression events of the mitochondrial genome between *C. albirostris* and *C. nippon* and another between *C. nippon* to *C. canadensis*. This scenario assumes that the phylogenetic tree based on nuclear data^45^ reflects the true evolutionary relationships among *Cervus* species. However, it may not be the case because the interspecific gene exchange between *C. albirostris* and *P. eldii*, *C. albirostris* and *R. unicolor* as well as *C. nippon* and *R. unicolor*^45^ could disturb the true phylogenetic signal in the nuclear genome. It would explain a more distant position of *C. albirostris* and *C. nippon* in the tree based on the nuclear data due to attraction by *Rusa*. Moreover, only one representative of *C. elaphus* was included in the nuclear genomic study^45^ and not from a native locality but from New Zealand, where there are many hybrids because of human-mediated translocations.

Although mtDNA can be subjected to introgression, mitochondrial genes are located on a single molecule and are inherited together^50,51^. Therefore, the individual markers carry the same phylogenetic signal in contrast to nuclear genes, which are more susceptible to incomplete lineage sorting and hidden gene paralogy^51–58^, which can cause disagreement between gene and species trees. To arrive at a better species tree within Cervini, additional detailed genomic analyses with more taxa and based on orthologous genes are necessary.

### Phylogenetic relationships within the Western lineage of *Cervus*

The obtained tree topology and the current distribution of mtDNA clades (Fig. 5) indicate that the ancestor of *C. elaphus* had a common origin with *C. hanglu* in Central Asia, and then migrated to Europe. A newly sequenced mitogenome of *C. elaphus barbarus*, which carries the B haplogroup^59^ originally native to the Italian Peninsula^60^, is sister to all other *C. elaphus* mitogenomes. Before its extinction on the Italian mainland this lineage was introduced by humans to Sardinia, Corsica and North Africa. Among the other red deer lineages, the western lineage A is sister to the monophyletic assembly of eastern haplogroups C, D and E so that the intraspecific relationships mirror geographic distribution.

Haplogroup A is the glacial refugial lineage in south-western Europe and at the end of the last glacial expanded from the Iberian Peninsula and southern France to Central and Northern Europe^2,13^. The other dominant haplogroup in Europe today is C, which recolonised the eastern parts of the continent from a refuge in the Balkans^2,13^. Taxonomically, A and C roughly represent the western and eastern European subspecies: *C. e. hippelaphus* or *C. e. elaphus* and *C. e. pannoniensis*, respectively. Haplogroups B, D and E are also in good accordance with the Mediterranean subspecies *C. e. corsicanus* on the Tyrrhenian islands and *C. e. barbarus* in North Africa (B), the recently described *C. e. italicus* in the Mesola Po delta region (D)^61^ and *C. e. maral* in Asia Minor and further east (E). Geographic outliers such as D haplogroup in a few Polish red deer^24^ may reflect true biogeographic patterns^3^ or human translocations.

The placement of haplogroup E sample from Iran within European haplogroups C and D suggests migrations from Balkans to Asia. Alternatively, haplogroup E could be a remnant of broader *Cervus* range in the past. Closer relationships between haplogroups C and E are confirmed in morphological similarities, e.g. massive antlers with a relatively simple crown, a frequent fourth tine or „dagger” below the crown and scarcely developed mane^47,62^.

### Phylogenetic relationships within the Eastern lineage of *Cervus*

All trees yielded a monophyletic eastern group of *Cervus* species with high support, comprising *C. albirostris, C. canadensis* and *C. nippon.* Based on these relationships and current geographic ranges of *Cervus*, it can be hypothesised that the ancestor of the Eastern lineage lived in Central China. *C. canadensis* expanded north-westwards giving rise to *sibiricus* but other populations evolved in China (*alashanicus* and *kansuensis*), from where another migration route led to the north and west (represented by *songaricus*) as well as in the opposite direction to North-eastern Asia, which is today inhabited by the subspecies *xanthopygus*. For a more detailed picture nuclear markers as well as data on *C. c. wallichi* are necessary. *C. nippon* shows two monophyletic, well-supported clades: on the mainland plus Taiwan and in Japan. The former lineage separated into a north-eastern subspecies *C. n. hortulorum*, a south-eastern subspecies *C. n. sichuanicus* and *C. n. kopschi*, whose common ancestor could have migrated to Taiwan, where it evolved into *C. n. taiouanus*.

The monophyly of Japanese deer suggests a single colonisation event and was also obtained based on shorter sequences of cytochrome b^13^, the control region^63^ and complete mitogenomes^64^. However, in the tree based on longer cytochrome b sequences^13^ and other trees based on the control region^65–67^, the northern and central subspecies grouped with the mainland clades, which suggests at least two colonisation events. Nevertheless, even when assuming the monophyly of Japanese deer, it is possible that the mitochondrial lineages of Japanese *C. nippon* split already on the Asian mainland and later colonized the islands in two waves from Sakhalin and the Korean Peninsula. A late arrival of sika in Japan would be supported by the oldest *C. nippon* remains found on Honshu, which are from 0.220 Mya^36^.

*C. canadensis nannodes*, native to California, is grouped with high support in the phylogenetic tree with *C. canadensis sibiricus*, which indicates that the American wapiti could carry mtDNA from this subspecies or its relatives. This close relationship is supported by evident affinities in morphology between *C. canadensis sibiricus* and North American wapitis^47^. It contrasts with the phylogenetic results based on the control region, which yields that the North American wapiti is related, albeit with poor support, with *C. canadensis xanthopygus*^16,30^ or *C. canadensis songaricus*^14^. Our results are more congruent with those based on cytochrome b, in which American wapiti were grouped with *C. canadensis sibiricus* and also *C. canadensis songaricus*^3,13–15^. According to Meiri et al.^68^, colonisation of North America occurred around 15 kya, and the northeast Siberian source population became extinct within the past 500 years.

### Estimating divergence times within Cervini

The best approach to estimate divergence times is to include calibration points within the studied group but most previous studies used only the split between Cervini and Muntiacini^8,27,28,31^. Only Doan et al.^13^ applied a point located within Cervini, namely the separation of *Axis* and *Rucervus* from other taxa. Therefore, besides the Cervini-Muntiacini split, we used three more points placed within Cervini.

**Figure 8:**
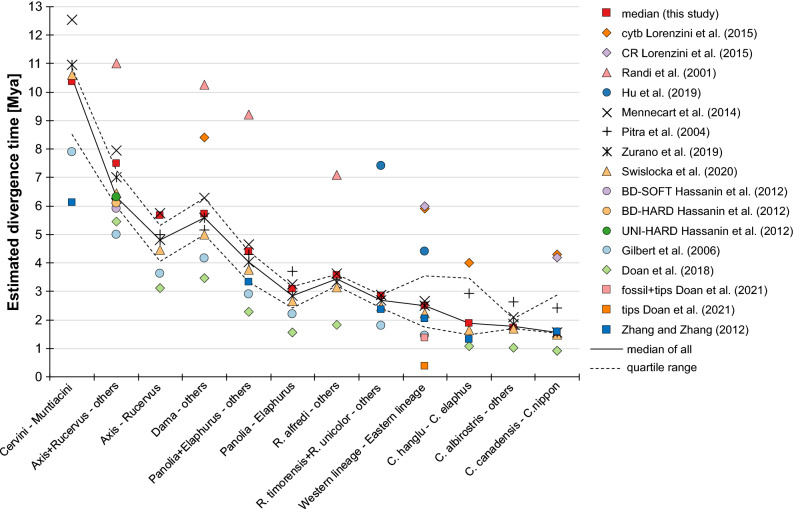
Comparison of selected divergence times of and within Cervini obtained in this study with those of previous analyses. BD-SOFT, BD-HARD and UNI-HARD indicate models with different priors uniform (UNI) and birth–death (BD) on divergence times, combined with either hard or soft fossil calibrations^31^.

Figure 8 shows a comparison of our estimates with those from previous studies for selected nodes in Cervini phylogeny. Our calculations correspond very well to the median calculated overall estimates and agree with some other authors^8,26,69^. Relatively old nodes were produced by methods that used old events as calibration point, e.g. the split between Cervidae and Bovidae (25.8 Mya)^14^, *Rangifer tarandus* and *Cervus nippon aplodontus* (13.9–13.6 Mya)^45^ as well as Cervini and Muntiacini (16.7–15.0 Mya)^27^, which is much older than the oldest known fossils of Muntiacini, i.e. 11–9 Mya^70^. Too old calibration points can result in imprecise estimates of divergence times for younger nodes. On the other hand, some authors applied points that were probably too young, e.g. assumptions on the oldest Muntiacini at 9–7 Mya^64,71^ or 10–8 Mya^31^, which shifted divergence events to more recent times. The inclusion of fossil calibrations can strongly impact molecular dating, e.g. young estimates of the node separating eastern and western deer were shifted back in time when a fossil calibration point was added to the tip dates of ancient samples^2^.

### Evolution of deer populations and mitochondrial protein-coding genes

The evolution of Cervini since the Late Miocene is connected to the expansion of open woodlands and grasslands associated with climate changes^72,73^. Accordingly, our results showed that many *Cervus* clades emerged since 2.6 Mya (the Early Pleistocene), when the oscillations of colder and warmer periods became more intense. Changes of climate to drier and colder could cause the expansion of grasslands and more open habitats as well as the appearance of new corridors, which enabled the migration and the dispersal of deer^15,31^. Also the uplift of the Tibetan Plateau and the Himalayan range in the Late Pliocene and Early Pleistocene could have influenced the spreading and diversification of deer in Asia^74^. The evolution of deer could follow a contraction–expansion model^6,75,76^. During glacials, its larger populations were fragmented into smaller isolated ones in refugia, which were subjected to bottlenecks and genetic drift. This resulted in distinct lineages with different gene pools, which later expanded during interglacials.

Analyses of nucleotide substitutions in protein-coding genes showed their different evolution in individual deer lineages, e.g. a higher proportion of more deleterious mutations in *C. nippon* and *C. hanglu*, which may be due to decreasing population sizes in the past and a larger impact of genetic drift in the small, isolated populations. We also found that two genes (*atp8* and *cox1*) were subjected to positive selection in some deer lineages, which could be associated with changing climatic and environmental conditions after migration of deer into new habitats, e.g. the expansion of the genus *Cervus* from Central Asia to Europe and its further diversification, split of *C. canadensis* into separate populations in Asia or the colonisation of separated islands by the Japanese deer subspecies.

The application of neutrality tests to sequences of protein-coding genes suggests that the Western lineage populations expanded recently after a bottleneck related to migration from Central Asia to Europe. On the other hand, *C. nippon* populations could have been subjected to isolation by geographic barriers or a recent bottleneck, which is reflected by the patchy distribution of its subspecies in Asia.

## Materials and methods

### Sample collection and DNA extraction

The novel mitogenome sequences were obtained for 11 individuals of *Cervus elaphus* representing all five phylogenetic haplogroups (A-E) as well as for two wapiti subspecies (Altai wapiti, *C*. *canadensis sibiricus* and Manchurian wapiti or izubra, *C*. *c*. *xanthopygus*). Table S3 includes the sampling localities and geographic coordinates for the analysed specimens. No live animals were used in our survey. We worked in the laboratory with only DNA isolates of *Cervus* from Italy (*C*. *elaphus italicus*) and Tunisia (*C*. *elaphus barbarus*), not their tissue samples. These samples were the same ones as used by Hajji et al.^59^ (for Tunisia red deer) and Zachos et al.^61^ (for Mesola red deer). Samples from Tunisia were legally collected in the wild from animal faecal samples. The DNA isolates of Mesola red deer originated from blood and hair tissue fragments collected from live-captured animals, marked and released over the years 1994–1998 during a long-term study of red deer ecology in a Natural Preserve with the permission of the Italian Ministry of Forestry which manages the protected area since 1954. Tissue samples of deer from Iran (*C*. *elaphus maral*) were collected from animals found dead in the wild. In the case of tissue samples from Poland and Russia, in these two countries, the hunting situation is very similar. In Russia, the red deer, including the Siberian (*C*. *canadensis sibiricus*) and Far Eastern (*C*. *canadensis xanthopygus*) subspecies, is a legal object of hunting in accordance with Federal Law No. 209-FZ of July 24, 2009 "On hunting and the conservation of hunting resources and amendments to certain legislative acts of the Russian Federation". In Poland, the list of hunting mammals was determined by the Order of the Minister of the Environment (Rozporządzenie Ministra Środowiska of 11 March 2005) (see Journal of Laws of 2005, No. 45, paragraph 433). The order came into effect on 17 August 2014. Thus, tissue samples from animals killed during legal hunting in Poland and Russia do not require specific permissions. Total genomic DNA was extracted using the DNeasy Blood & Tissue Kit (Qiagen, Hilden, Germany) following the manufacturer’s protocol.

### PCR and sequencing

The PCR thermal cycling was performed in 5 µL reaction volumes containing 2 µL genomic DNA (~ 20 ng), 1.7 µL Qiagen Multiplex PCR Master Mix (1 ×), 0.3 µL primer mix (0.2 µM of each primer), and 1 µL RNase-free water. The reaction conditions were the same for all used primer pairs and consisted of an initial denaturation step at 95 °C for 15 min, followed by 35 cycles at 94 °C for 30 s for denaturation, annealing for 90 s, extension at 72 °C for 60 s, and final elongation for 30 min at 60 °C. For details of the annealing temperature for different primer pairs see Table S4. We applied a set of 37 primer pairs for rapid amplification of deer mitochondrial genomes belonging to the Western and Eastern lineages, and to generate overlapping reads. Table S4 contains the primers used in this study, including 27 primer pairs newly designed with the FastPCR software^77^ on the basis of available mitogenomes of *Cervus* (Table S5). The amplicons were purified with the shrimp alkaline phosphatase (SAP) and Exonuclease I (Thermo Scientific) in an enzymatic reaction following the manufacturer’s protocol. Purified PCR products were bidirectionally sequenced using a BigDye™ Terminator Cycle Sequencing Kit v.3.1 (Applied Biosystems, Foster City, CA, USA). Unincorporated dideoxynucleotides were eliminated from the sequencing reaction with an ExTerminator Kit (A&A Biotechnology, Gdynia, Poland), and sequences were analysed on an automated ABI 3130 Genetic Analyzer (Applied Biosystems, Foster City, CA, USA).

### Sequence analysis

Mitogenomic sequences were manually revised and aligned using the sequence-editing program BioEdit, version 7.0.5.3^78^. All analysed protein-coding genes, as well as tRNA and rRNA genes, were identified using the MITOS online mitochondrial genome annotation server^79^ and the reference mitogenome sequences of *Bos taurus* (NC_006853). To avoid nuclear DNA sequences of mitochondrial origin (pseudogenes/numts), we also checked all coding regions for open reading frames and stop codons.

### Statistical analyses

For statistical analyses, we applied the Shapiro–Wilk test to for normality of the studied variables and Levene’s test to test for homogeneity of variances in the analysed groups. As these assumptions were not fulfilled, we applied non-parametric tests. The difference in mitogenome length and Provean scores between deer lineages were verified by means of a Kruskal–Wallis test with Dunn's post-hoc test in pairwise comparisons between the groups. The pairwise proportion test was also applied in the comparison of distributions of deleterious mutations between these groups. In the pairwise comparisons, we applied the Benjamini–Hochberg method^80^ for p-value correction to control for the false discovery rate. Differences were considered significant if p-values were lower than 0.05. Statistical tests and correspondence analyses of nucleotide composition of mitochondrial sequences were performed with the R software using the packages car, FactoMineR, FSA and stats^81^.

### Phylogenetic analyses of mitogenome sequences

Phylogenetic analyses were performed on complete mitogenomic sequences—13 red deer and wapiti mitogenomes newly obtained in this study and 58 mitogenomes downloaded from GenBank including 24 *Cervus* mitogenomes and 34 other representatives of Cervinae (Table S5). We studied all mitogenomic loci: control region, 13 protein-coding genes, 12S and 16S rRNAs, and 22 tRNAs. The sequences were aligned in MAFFT using an accurate algorithm L-INS-i with 1000 cycles^82^. Sites of protein-coding sequences suitable for phylogenetic analyses were selected in GBlocks assuming codon organization of the sequences^83^, whereas poorly aligned regions in other sequence types were removed using trimAl applying the best automated method^84^. The resulting alignments were inspected in JalView^85^. The final alignment consisted of 16,306 bp.

We run three phylogenetic analyses: the maximum likelihood method in IQ-TREE^86^, as well as Bayesian inference in MrBayes^87^ and PhyloBayes^88^. We took into account all potential partitions in finding the best substitution models, i.e. three codon positions for individual protein-coding genes and separate partitions for individual RNA genes and the control region.

In IQ-TREE analyses, ModelFinder^89,90^ was used to select the best-fitting scheme of substitution models (Table S6). To assess significance of branches, we applied the Shimodara-Hasegawa-like approximate likelihood ratio test (SH-aLRT) with 10,000 replicates and non-parametric bootstrap with 1000 replicates. In the tree search, we used a more thorough NNI (nearest neighbor interchange) search and assumed 1000 initial parsimony trees and 100 top initial parsimony trees to optimize with the NNI search to initialize the candidate set.

In MrBayes, we used the partitioned scheme of substitution models from PartitionFinder^91^ (Table S6). Nevertheless, we applied mixed models to specify appropriate substitution models across the large parameter space^92^. The models describing heterogeneity rate across sites were taken from the PartitionFinder results. Two independent runs using 32 Markov chains were applied. The trees were sampled every 100 generations for 10,000,000 generations. We generated a posterior consensus tree based on the trees from the last 6,581,000 generations, when the runs had reached convergence, i.e. the standard deviation of split frequencies had stabilized and was much below the recommended threshold of 0.01.

In PhyloBayes, we applied the CAT-GTR+Γ model with parameters inferred from the data. Two independent Markov chains were run for 100,000 generations. The last 50,000 were collected from each chain to compute a posterior consensus after reaching convergence, when the largest discrepancy observed across all bipartitions (maxdiff) was much below the recommended threshold of 0.1. The consensus tree of the trees obtained by the three approaches was calculated in IQ-TREE.

The proportion of different sites (p-distance) between deer sequences was calculated in MEGA 11^93^. They were compared with distances obtained from the MrBayes phylogenetic tree calculated as the sum of branch lengths.

### Inferring ancestral sequences and analysis of substitutions in protein-coding genes

Ancestral sequences of 13 individual mitochondrial proteins of deer as well as the most probable amino acid substitutions were inferred using the maximum likelihood method in MEGA 11^93^ for each protein alignment applying the best substitution model found in MEGA 11. *Rusa unicolor* and *R. timorensis* were used as outgroup taxa. Based on the inferred sequences and substitutions, we assessed the deleterious effect of these mutations using the standalone version of Provean with non-redundant GenBank database and default assumptions, i.e. a clustering of BLAST hits was performed by CD-HIT assuming 75% global sequence identity, the top 30 clusters of closely related sequences from the supporting sequence set were used to generate the prediction, and the threshold − 2.5 was assumed for separation of deleterious and neutral mutations^94^. To check for the presence of positive selection in deer protein-coding genes, we applied all methods and models with codon-based Z-test and Fisher's exact test available in MEGA 11. Tajima's test of neutrality was also conducted with this software, whereas Fu’s test was done with DnaSP^95^.

Ancestral sequences of selected mitochondrial genes in the common ancestor of *Cervus* were inferred in IQ-TREE using the fixed tree found in this program based on the complete mitogenomic sequences including *Rusa unicolor* and *R. timorensis* as an outgroup. For each data set we applied the best-fitting scheme of substitution models taking into account appropriate partitions.

### Molecular dating

Divergence times were estimated using BEAST 1.10.4^96^ for two data sets, one including representatives of Muntiacini and Cervini (as described above) and the other consisting of 39 *Cervus* sequences only. We assumed substitution models as proposed by PartitionFinder (Tables S6 and S7). For the first data set we introduced four calibration points. The normal distribution with the mean of 10.825 and a standard deviation of 1.1215 with 95% HPD interval of 12.67–8.98 Mya was assumed for the split of Cervinae into Cervini and Muntiacini based on our previous estimations conducted for cervid mitogenomes^8^. This corresponds well with the oldest representative of Muntiacini dated to 11–9 Mya^70^. Additionally, we assumed the lognormal distribution prior with the offset of 5.3 Mya for the split of *Axis* and *Rucervus* based on the oldest fossils of *Axis* dated to 6.6–5.3 Mya^97,98^. This assumption also agrees with the oldest *Rucervus* dated to 7–5 Mya^99^. The divergence of *Rusa* and *Cervus* clades was assumed according to the oldest *Rusa* dated to 3.4–2.6 Mya^100^. We applied the lognormal distribution prior with the offset of 2.6 Mya. Finally, we used the exponential distribution prior with the offset of 1.95 Mya and the 95% HPD of 2.6 Mya for the separation of two *Cervus* clades: *C. elaphus* + *C. hanglu* and *C. canadensis* + *C. nippon* + *C. albirostris*. This assumption was based on the fossil *C. nestii* dated to 2.1–1.95 Mya from Olivola^101^, which probably belongs to the lineage of *C. elaphus* + *C. hanglu*^33,35^. For the *Cervus* set, we assumed the normal distribution for the age of the root with the mean of 2.59 Mya and 95% HPD 2.03–3.15 Mya as obtained in the dating for the Muntiacini and Cervini set.

In the case of the Muntiacini and Cervini set, we applied the Yule process model, whereas for the *Cervus* set, we used the coalescent constant size model because it was yielded as the best fitting according to the marginal likelihood values calculated in path sampling and the stepping stone algorithm^102,103^. In these calculations, we applied a chain length of 10,000,000 and a number of path steps of 20. Besides the coalescent constant size model, we considered the following models: coalescent Bayesian SkyGrid, coalescent GMRF Skyride with time-aware and uniform smoothing, coalescent Bayesian Skyline piecewise-constant and piecewise-linear, speciation Yule and Birth–Death process. For both data sets, we applied the lognormal relaxed clock model rather than the strict clock because the coefficient of variation of the relaxed clock was quite high (0.43 and 0.61, respectively). Posterior distributions of parameters were estimated with a sampling frequency of 1000 steps for 1 billion and 800 million generations for the first and the second dataset, respectively.

Convergence and sufficient sampling were checked using loganalyzer and Tracer 1.7 (http://beast.bio.ed.ac.uk/Tracer). All parameters had an Effective Sample Size (ESS) exceeding 200. The phylogenetic trees were summarized in TreeAnnotator with a 10% burn-in and assuming common ancestor heights. The final trees were visualized in FigTree 1.4.3 (http://tree.bio.ed.ac.uk/software/figtree).

## Supplementary Information

Supplementary Information.

## Data Availability

Raw sequencing data of the deep-sequenced genomes are available on the National Center for Biotechnology Information under project accession number OL679912–OL679924 (https://www.ncbi.nlm.nih.gov/nuccore/OL679912; https://www.ncbi.nlm.nih.gov/nuccore/OL679913; https://www.ncbi.nlm.nih.gov/nuccore/OL679914; https://www.ncbi.nlm.nih.gov/nuccore/OL679915; https://www.ncbi.nlm.nih.gov/nuccore/OL679916; https://www.ncbi.nlm.nih.gov/nuccore/OL679917; https://www.ncbi.nlm.nih.gov/nuccore/OL679918; https://www.ncbi.nlm.nih.gov/nuccore/OL679919; https://www.ncbi.nlm.nih.gov/nuccore/OL679920; https://www.ncbi.nlm.nih.gov/nuccore/OL679921; https://www.ncbi.nlm.nih.gov/nuccore/OL679922; https://www.ncbi.nlm.nih.gov/nuccore/OL679923). Sequences in Fasta and GenBank formats are also available on FigShare under https://doi.org/10.6084/m9.figshare.20337573. Other data supporting the findings of the study are available in this article and its Supplementary Information files, or from the corresponding authors upon request.
