# Supplementary material for: Phylogeny and evolution of the genus Cervus (Cervidae, Mammalia) as revealed by complete mitochondrial genomes
Source: Sci Rep. 2022 Sep 30;12:16381. doi: 10.1038/s41598-022-20763-x (PMC9525267; doi:10.1038/s41598-022-20763-x)
Supplement: Supplementary file 1 — Supplementary Information. [file 41598_2022_20763_MOESM1_ESM.pdf]

Table S1. The number and proportion of deleterious and neutral mutations accumulated in mitochondrial protein genes in various deer lineages.

Species	Number		Proportion	
	deleterious	neutral	deleterious	neutral
C. albirostris	6	40	0.13	0.87
C. canadensis	5	37	0.12	0.88
C. elaphus	12	65	0.16	0.84
C. hanglu	7	14	0.33	0.67
C. nippon	48	82	0.37	0.63
R. unicolor and *timorensis*	10	34	0.23	0.77

Table S2. Results of Tajima's and Fu's neutrality tests calculated for nucleotide sequences of protein-coding genes from various deer groups.

Group	m	S	π	D	Fs
Ce+Ch	16	773	0.014	-1.30	-0.04
Ce	15	457	0.011	-0.59	-0.26
Cc+Cn+Ca	22	1202	0.027	-0.23	-0.07
Cc+Cn	20	984	0.025	0.07	0.07
Cc	8	241	0.008	-0.34	1.00
Cn	12	707	0.020	-0.11	1.11
mainland Cn	9	318	0.012	0.97	1.24

m - number of sequences, S - Number of segregating sites, π - nucleotide diversity, D - the Tajima's test statistic, Fs - the Fu's test statistic; Ca - *C. albirostris*; Cc - *C. canadensis*; Ce - *C. elaphus*; Ch - *C. hanglu*; Cn - *C. nippon.*

Table S3. List of the 13 mitochondrial genomes of *Cervus* obtained in this study, with their sampling locality, geographic coordinates and accession numbers.

Sample ID	Species/subspecies	Locality	Coordinates	Length [bp]	GenBank acc. num.
WEST1	*C. elaphus*	Alfredówka, Poland	50°27' N 21°45' E	16351	OL679912
WEST2	*C. elaphus*	Maków Podhalański, Poland	49°43' N 19°40' E	16351	OL679913
WEST3	*C. elaphus*	Trzebielino, Poland	54°11' N 17°05' E	16352	OL679914
WEST4	*C. elaphus*	Bytnica, Poland	52°09' N 15°10' E	16352	OL679915
EAST1	*C. elaphus*	Łazy, Poland	50°25' N 19°23' E	16355	OL679916
EAST2	*C. elaphus*	Ocieka, Poland	50°08' N 21°35' E	16355	OL679917
EAST3	*C. elaphus*	Lądek Zdrój, Poland	50°20' N 16°52' E	16354	OL679918
MAR1	*C. elaphus maral*	Mazandaran, Iran	36°27' N 52°06' E	16350	OL679919
MES1	*C. elaphus*	Rymanów, Poland	49°33' N 21°51' E	16351	OL679920
MES2	*C. elaphus italicus*	Riserva Naturale Bosco della Mesola, Italy	44°51' N 12°14' E	16351	OL679921
TUN1	*C. elaphus barbarus*	Zoological Park Belvédère in Tunis, from El Feidja National Park, Tunisia	36°29' N 8°19' E	16353	OL679922
SIB1	*C. canadensis sibiricus*	Kyzyl, Tuva, Russia	52°10' N 95°45' E	16428	OL679923
XAN1	*C. canadensis xanthopygus*	Terneysky District, Primorsky Krai, Russia	45°18' N 136°78' E	16353	OL679924

Table S4. List of primer pairs used for PCRs and DNA sequencing of *Cervus elaphus* (haplogroups A-E), *C. canadensis sibiricus* (SIB) and *C. c. xanthopygus* (XAN) mitogenomes.

Primer	Primer sequence	mtDNA position	Tm [°C]	Product length [bp]	Amplified genes	Primer source	*C. elaphus*					*C. canadensis*	
							A	B	C	D	E	SIB	XAN
12S-FW<br>12S-REV	GGTAAATCTCGTGCCAGCCA<br>TCCAGTATGCTTACCTTGTTACGAC	00295<br>01006	57.3<br>56.2	711	<12s_rRNA>	Fajardo et al. (2007)	●	●	●	●	●	●	●
00868cee_F<br>01461cee_R	AGTGCTTAGTTGAACTAGGCCAT<br>TTCTGGACAACCAGCTATCACCA	00869<br>01461	55.8<br>57.6	592	<12s_rRNA…tRNA-Val…16s_rRNA>	This study		●		●			
00871c_F<br>02052c_R	TGCTTAGTTGAATTAGGCAATG<br>AGAGAACAAGTGATTATGCTACC	00871<br>02049	51.3<br>52.2	1178	<12s_rRNA…tRNA-Val…16s_rRNA>	Matosiuk et al. (2014)	●		●		●	●	●
01438cee_F<br>02033cee_R	TGGTGATAGCTGGTTGTCCAGAA<br>TACCTTTGCACGGTCAGGATA	01439<br>02030	57.6<br>55.3	591	<16s_rRNA>	This study		●		●			
01950c_F<br>03402c_R	ACCTCCAGCATAACTAGTATTGG<br>AATGGTCCTGCTGCATACTCTA	01947<br>03401	53.7<br>55.2	1454	<16s_rRNA…tRNA-Leu…ND1>	Matosiuk et al. (2014)	●	●	●	●	●	●	●
02233cee_F<br>02667cee_R	TGGGCTAACAGCTTTGGTTG<br>AAGCCCTTTTCTAGGGCAAG	02231<br>02664	55.8<br>55.1	433	<16s_rRNA…tRNA-Leu>	This study		●					
02683cee_F<br>03412cee_R	CCCGGTAATTGCGTAAAACT<br>ATAGGGCAAATGGTCCTGCT	02681<br>03409	53.2<br>56.2	728	<tRNA-Leu…ND1>	This study		●					●
03140c_F<br>04165c_R	CTACGAGCAGTAGCTCAAACA<br>ACAGTTCATTGGCCTGAAAATA	03139<br>04165	54.1<br>52.5	1026	<ND1…tRNA-Ile…tRNA-Gln…trNA-Met…ND2>	Matosiuk et al. (2014)	●	●	●	●	●	●	●
3910cee_F<br>4300cee_F2<br>5430cee_R	CCTTCCCGTACTAATAAACC<br>TCATCAGGCCTAATTCTACT<br>TATGCCTGCTCARGCACCAA	03896<br>04281<br>05417	50.0<br>-<br>56.0	1521	<tRNA-Met…ND2…tRNA-Trp…tRNA-Ala…tRNA-Asn…tRNA-Cys…tRNA-Tyr…COX1>	This study	●		●		●	●	
03986cee_F<br>04812cee_R	TGACTACTTGTCTGAATCGG<br>CAGTGGAATATGTGAGTCGT	03984<br>04809	51.3<br>51.6	825	<ND2>	This study		●		●		●	●
04637cee_F<br>05475cee_R	CTAGTCCTCATTACCCTCCT<br>AGTACCAGGTTGGCCCAGTTC	04635<br>05471	52.1<br>59.1	836	<ND2…tRNA-Trp…tRNA-Ala…tRNA-Asn…tRNA-Cys…tRNA-Tyr…COX1>	This study		●		●			●
COX1_F<br>COX1_R	TCAGCCATTTTACCTATGTTCA<br>ATRTAGCCAAARGGTTCTTTTT	05319<br>06145	51.7<br>48.5	826	<tRNA-Tyr…COX1>	GQ336878.1	●	●	●	●	●	●	●
05986c_F<br>06999c_R	TTCTTTGACCCAGCAGGAGGC<br>ACCTGTAACTTAACCTTGACAAAG	05988<br>07000	59.6<br>53.0	1012	<COX1…tRNA-Ser…tRNA-Asp>	Matosiuk et al. (2014)	●	●	●		●	●	●
06011cee_F<br>06815cee_R	TCTTTGACCCAGCAGGAGG<br>GTTCTACGGTTGAGACTTCTCG	05989<br>06792	56.6<br>54.8	803	<COX1>	This study				●			
06381cee_F<br>07029cee_R	ACAGTTGGAGGCTTGACTGGAAT<br>GCCATATGAGATGTACGGGG	06378<br>07025	58.2<br>54.9	647	<COX1…tRNA-Ser…tRNA-Asp…COX2>	This study				●			
06680cee_F<br>07760cee_R	GCATACACAACATGAAAYACCA<br>CTAGCTTAAAAGGCTAGCGCTATT	06669<br>07745	52.2<br>54.8	1076	<COX1…tRNA-Ser…tRNA-Asp…COX2…tRNA-Lys>	This study	●		●		●	●	●
06822cee_F<br>07740cee_R	AATGGATGTCCCCCACCATATCA<br>AAGGCTAGCGCTATTCTAGC	06819<br>07736	57.6<br>54.1	917	<COX1…tRNA-Ser…tRNA-Asp…COX2…tRNA-Lys>	This study		●		●			

07723cee_F<br>08650cee_R	GAATAGCGCTAGCCTTTTAAGC<br>GTGATAAGCGTGGGTTTGGT	07721<br>08644	54.2<br>55.4	923	<tRNA-Lys…ATP8…ATP6…COX3>	This study		●		●			●
07540cee_F<br>08940cee_R	GCAATTCCAGGTCGCCTCAAC<br>AGGCTTGAGTGGTAAAATGCTC	07546<br>08934	59.0<br>55.4	1388	<COX2…ATP8…ATP6…COX3>	This study	●	●	●	●	●	●	●
08750cee_F<br>09560cee_R	CAATAGTCCTATTAACACTTGGC<br>TAATTGAGGGAGTCAGAATGC	08733<br>09551	51.4<br>52.1	818	<COX3…tRNA-Gly…ND3>	This study	●		●		●	●	●
08820cee_F<br>09610cee_R	GACACCATACTCCAGCCGTC<br>GATCCTATGGGGTCGAATCCAC	08823<br>09607	57.3<br>56.9	784	<COX3… tRNA-Gly…ND3>	This study		●		●			
09302c_F<br>10872c_R	AAGCCACCATTTTGGTTTTGAAGC<br>GCTGCAAGGACTATAGAGCCTG	09304<br>10874	57.5<br>57.2	1570	<COX3… tRNA-Gly…ND3…tRNA-Arg…ND4L…ND4>	Matosiuk et al. (2014)	●	●	●	●	●	●	●
09930cee_F<br>10304cee_R	AGTGGCCCTAGCAGGACTA<br>TAAGGAGAAGGCTTGTGAGGCT	09925<br>10300	56.7<br>57.5	375	<ND4L…ND4>	This study		●		●	●	●	
10349cee_F<br>11010cee_R	TTCCGACTCCCTATCAGCAC<br>GGAGGCAGATTGAGCTGGTT	10347<br>11008	55.9<br>57.2	661	<ND4>	This study		●		●			
10740cee_F<br>12060cee_R	AGTACAACCCCTACCTAACTCC<br>TCATGTAACAAATAGTGCTACTG	10722<br>12048	55.1<br>50.4	1326	< ND4…tRNA-His…tRNA-Ser…tRNA-Leu…ND5>	This study	●		●		●	●	
10730cee_F<br>11480cee_R	CCTACCTAACTCCTGATCAAACG<br>TCCCGTGTAAAGGAGGGTGT	10731<br>11474	54.7<br>57.5	743	<ND4>	This study		●		●			●
11265cee_F<br>12058cee_R	CAAGCCTAACTAACTTAGCTCTC<br>GATGGATCATGTAACAAATAGTGC	11261<br>12054	52.7<br>52.1	793	<…tRNA-His…tRNA-Ser…tRNA-Leu…ND5>	This study		●		●			●
11710cee_F<br>13150cee_R	TTAGAGGATGACAGAAATCCGTT<br>TCCTGCGAAAAGGCTTCCAAT	11689<br>13149	53.3<br>57.1	1460	< tRNA-Leu…ND5>	This study	●		●		●	●	●
11920cee_F<br>12700cee_R	ATCCATACTGGCCAAGAAATA<br>ATTATGAGGCCTAGTTGGCT	11917<br>12695	51.0<br>53.0	778	<ND5>	This study		●		●			
12570cee_F<br>13363cee_R	AGTTTGCACAATCCATCCTACTA<br>CGGTGTATAATTGTGGGGAAATA	12566<br>13358	53.6<br>52.6	792	<ND5>	This study		●		●			
13020cee_F<br>14240cee_R	ACCTCCTTCACAGCCATCTACA<br>TGGGAGGTCAATAAATGCGTTG	13000<br>14225	57.2<br>55.6	1225	<ND5…ND6…tRNA-Glu..cytb>	This study	●		●		●	●	●
13268cee_F<br>14232cee_R	ATCAGCAACATAACCCAAAACCT<br>GCTGGGAGGTCAATAAATGCG	13264<br>14227	54.9<br>56.5	963	<ND5…ND6…tRNA-Glu..cytb>	This study		●		●			
14096cee_F<br>15235cee_R	CTCACATGGAATCTAACCATGAC<br>TAAGACAGATGCTAGTTGTCCAA	14091<br>15230	53.3<br>53.2	1139	< tRNA-Glu…cytb>	This study		●					
ML103<br>ML104	GACTAATGATATGAAAAACCATCGTTG<br>TTGTTCTTCATCTCTGGTTTACAAGAC	14111<br>15354	52.8<br>55.0	1243	<tRNA-Glu…cytb…tRNA-Thr>	Chikuni et al. (1995)	●		●	●	●	●	●
15180cee_F<br>15530cee_R	ACATGAATCGGAGGACAACCA<br>TGTTGAGTGGAAAGCTGTATTG	15165<br>15531	55.8<br>53.2	366	<cytb…tRNA-Thr…tRNA-Pro…d-loop>	This study	●	●	●	●	●	●	●
tRNA-PRO<br>tRNA-PHE	CACCATCAACCCCCAAAGCTGAAG<br>CAGTGCCTTGCTTTGGGTTAAGC	15393<br>00034	60.7<br>59.5	1003	<tRNA-Pro…d-loop…tRNA-Phe>	Wood and Phua (1996)	●	●	●	●	●	●	●
16104c_F<br>00487c_R	CTGGACTTAACTGCATCTTGAGC<br>CAGTTTGGGTCTTAGCTATCGTG	16093<br>00487	55.9<br>55.4	756	<d-loop…tRNA-Phe…12s_RNA>	Matosiuk et al. (2014)	●	●	●	●	●	●	●

● primers pairs used in a given phylogenetic lineage/haplogroup analysi

Table S5. List of *Cervinae* representatives and GenBank accession numbers of their mitogenomic sequences used in phylogenetic analyses. The names are given as found in GenBank, not necessarily according to the presently accepted taxonomy.

**Acc. num.**	**Taxon**
NC_020680.1	*Axis axis*
MF435989.1	*Axis porcinus*
MH443787.1	*Axis porcinus*
MH443790.1	*Axis porcinus*
NC_020681.1	*Axis porcinus*
NC_050863.1	*Cervus canadensis*
NC_039923.1	*Cervus canadensis kansuensis*
KJ025072.1	*Cervus canadensis songaricus*
NC_014703.1	*Cervus canadensis songaricus*
KP172593.1	*Cervus elaphus*
MF872247.1	*Cervus elaphus*
MF872248.1	*Cervus elaphus*
NC_007704.2	*Cervus elaphus*
KU942399.1	*Cervus elaphus alxaicus*
KT290948.1	*Cervus elaphus hippelaphus*
KX449334.1	*Cervus elaphus macneilli*
NC_013840.1	*Cervus hanglu yarkandensis*
NC_006993.1	*Cervus nippon centralis*
HQ191428.1	*Cervus nippon hortulorum*
KR868807.1	*Cervus nippon hortulorum*
KX689229.1	*Cervus nippon hortulorum*
NC_013834.1	*Cervus nippon hortulorum*
JN389444.1	*Cervus nippon kopschi*
NC_016178.1	*Cervus nippon kopschi*
NC_018595.1	*Cervus nippon sichuanicus*
EF058308.1	*Cervus nippon taiouanus*
NC_008462.1	*Cervus nippon taiouanus*
NC_007179.1	*Cervus nippon yakushimae*
NC_006973.1	*Cervus nippon yesoensis*
NC_020700.1	*Dama dama*
NC_024819.1	*Dama mesopotamica*
NC_008749.1	*Elaphodus cephalophus*
NC_050383.1	*Elaphodus cephalophus cephalophus*
JN632632.1	*Elaphurus davidianus*
NC_018358.1	*Elaphurus davidianus*
NC_004577.1	*Muntiacus crinifrons*
NC_041100.1	*Muntiacus feae*
NC_048506.1	*Muntiacus gongshanensis*
NC_004563.1	*Muntiacus muntjak*
NC_036430.1	*Muntiacus putaoensis*
NC_004069.1	*Muntiacus reevesi*
NC_008491.1	*Muntiacus reevesi micrurus*
MK050505.1	*Muntiacus vaginalis*
NC_016920.1	*Muntiacus vuquangensis*
JN632697.1	*Panolia eldii*
NC_014701.1	*Panolia eldii*
KU133959.1	*Panolia eldii eldii*
JN632690.1	*Przewalskium albirostris*
MF966595.1	*Przewalskium albirostris*
NC_016707.1	*Przewalskium albirostris*
NC_020743.1	*Rucervus duvaucelii*
MG788693.1	*Rucervus duvaucelii branderi*
NC_045060.1	*Rucervus duvaucelii branderi*
NC_020744.1	*Rusa alfredi*
NC_020745.1	*Rusa timorensis*
NC_031835.1	*Rusa unicolor*
KY946815.1	*Rusa unicolor hainana*
EF035448.1	*Rusa unicolor swinhoei*

Table S6. Substitution models and partitions applied in phylogenetic analyses of data set including representatives of Muntiacini and Cervini. A digit 1, 2 or 3 after the dot indicates the codon position in protein-coding genes.

Software	Substitution model	Partitions
IQ-TREE	TIM2+F+R3	atp6.1, atp8.1, cyt.1, nd2.1, nd4.1, nd5.1, rRNA, 12s, rRNA, 16s, tRNA-Arg, tRNA-Asp, tRNA-Gly, tRNA-His, tRNA-Ile, tRNA-Leu2, tRNA-Lys, tRNA-Phe, tRNA-Thr, tRNA-Trp, tRNA-Tyr, tRNA-Val
	HKY+F+R2	atp6.2, cox1.2, cox2.2, cox3.2, cyt.2, nd1.2, nd2.2, nd3.2, nd4L.2, nd4.2, nd5.2, nd6.2, tRNA-Met
	TN+F+I+Γ4	atp6.3, atp8.3, cox1.3, cox2.3, nd4L.3
	K3Pu+F+R3	atp8.2, tRNA-Ser2, control region
	TNe+I+Γ4	cox1.1, cox2.1, cox3.1, nd1.1, nd3.1, nd4L.1
	TIM2+F+I+Γ4	cox3.3, cyt.3, nd1.3, nd2.3, nd3.3, nd4.3, nd5.3
	HKY+F+I+Γ4	nd6.1, tRNA-Ala, tRNA-Asn, tRNA-Cys, tRNA-Gln, tRNA-Glu, tRNA-Leu1, tRNA-Pro, tRNA-Ser1
	HKY+F+Γ4	nd6.3
MrBayes	mixed+I+Γ5	nd4.1, atp6.1, cyt.1
	mixed+I+Γ5	nd5.2, nd2.2, atp6.2
	mixed+I+Γ5	atp6.3, cox1.3, cox2.3
	mixed+Γ5	tRNA-Ser2, atp8.1, tRNA-Asp, tRNA-Lys
	mixed+I+Γ5	atp8.2, tRNA-Arg
	mixed+Γ5	atp8.3
	mixed+I+Γ5	tRNA-Cys, cox1.1
	mixed+I	cox2.2, cox3.2, cox1.2
	mixed+Γ5	nd3.1, cox2.1
	mixed+I+Γ5	cox3.1, tRNA-Met, tRNA-Ile
	mixed+I+Γ5	cox3.3, nd1.3, nd4L.3, nd4.3, cyt.3, nd5.3
	mixed+I+Γ5	cyt.2
	mixed+I+Γ5	nd1.1, tRNA-Trp, nd4L.1
	mixed+I+Γ5	nd1.2, nd3.2, nd4L.2, nd4.2
	mixed+I+Γ5	nd2.1, tRNA-Val, tRNA-Thr, nd5.1
	mixed+Γ5	nd2.3, nd3.3
	mixed+I+Γ5	nd6.1
	mixed+I	nd6.2
	mixed+Γ5	nd6.3
	mixed+I+Γ5	rRNA 12s, tRNA-Gly, tRNA-Tyr, rRNA 16s, tRNA-Leu2
	mixed+I+Γ5	tRNA-Gln, tRNA-Glu, tRNA-Ala
	mixed+I	tRNA-Leu1, tRNA-Asn, tRNA-Pro, tRNA-Ser1
	mixed+I+Γ5	tRNA-His, tRNA-Phe
	mixed+I+Γ5	control region
Beast	TRN+I+Γ5	nd4.1, atp6.1, cyt.1
	TRN+I+Γ5	nd5.2, nd2.2, atp6.2
	TRN+I+Γ5	atp6.3, cox1.3, cox2.3
	HKY+Γ5	tRNA-Ser2, atp8.1, tRNA-Asp, tRNA-Lys
	HKY+I+Γ5	atp8.2, tRNA-Arg
	HKY+Γ5	atp8.3
	TIMEF+I+Γ5	tRNA-Cys, cox1.1
	HKY+I	cox2.2, cox3.2, cox1.2
	TRNEF+Γ5	nd3.1, cox2.1
	TRNEF+I+Γ5	cox3.1, tRNA-Met, tRNA-Ile
	GTR+I+Γ5	cox3.3, nd1.3, nd4L.3, nd4.3, cyt.3, nd5.3
	TRN+I+Γ5	cyt.2
	SYM+I+Γ5	nd1.1, tRNA-Trp, nd4L.1
	GTR+I+Γ5	nd1.2, nd3.2, nd4L.2, nd4.2
	TRN+I+Γ5	nd2.1, tRNA-Val, tRNA-Thr, nd5.1
	TRN+Γ5	nd2.3, nd3.3
	GTR+I+Γ5	nd6.1
	HKY+I	nd6.2
	HKY+Γ5	nd6.3
	GTR+I+Γ5	rRNA 12s, tRNA-Gly, tRNA-Tyr, rRNA 16s, tRNA-Leu2
	TRN+I+Γ5	tRNA-Gln, tRNA-Glu, tRNA-Ala
	HKY+I	tRNA-Leu1, tRNA-Asn, tRNA-Pro, tRNA-Ser1
	HKY+I+Γ5	tRNA-His, tRNA-Phe
	TIM+I+Γ5	control region

Table S7. Substitution models and partitions applied in Beast analyses of data set including only representatives of *Cervus*. A digit 1, 2 or 3 after the dot indicates the codon position in protein-coding genes.

Substitution model	Partitions
HKY+I	atp6.1, tRNA-Ser2
TIM+I	atp6.2, nd2.2, nd4L.2, nd3.2, nd4.2
TRN+Γ5	atp6.3
HKY+Γ5	atp8.1, atp8.2
K81UF+I	atp8.3, cox2.3
TRN+I	cox1.1
HKY+I	nd6.2, cox1.2, cytb.2, cox3.2
GTR+Γ5	cox1.3
K80+I	nd4L.1, cox2.1
F81	cox2.2
TRNEF+I+Γ5	nd1.1, tRNA-Met, nd3.1, cox3.1
TRN+Γ5	cox3.3, nd5.3
TRN+I	cytb.1, nd5.1
TRN+Γ5	cytb.3
K81UF+I	nd1.2, nd5.2
TRN+Γ5	nd2.3, nd3.3, nd4.3, nd1.3, nd4L.3
TRN+I+Γ5	nd2.1, tRNA-Val, rRNA 12s, tRNA-Trp, nd4.1, tRNA-Thr
GTR	nd6.1
TVM	nd6.3
HKY+I+Γ5	tRNA-Leu1, tRNA-Leu2, tRNA-Gly, tRNA-Arg, rRNA 16s
TRN+I	tRNA-Ala, tRNA-Glu
HKY	tRNA-Cys, tRNA-Gln, tRNA-Asn, tRNA-Ser1, tRNA-Pro
HKY+Γ5	tRNA-Lys, tRNA-Phe, tRNA-Asp, tRNA-Tyr
HKY	tRNA-Ile, tRNA-His
TIM+I+Γ5	control region

Figure S1. Correspondence analysis plots obtained for the nucleotide composition of 13 protein-coding genes (PCG), RNA genes and control region (CR) from various *Cervus* taxa: a - *C. albirostris*; c - *C. canadensis*; c_ala - *C. canadensis alashanicus*; c_kan - *C. canadensis kansuensis*; c_sib - *C. canadensis sibiricus*; c_son - *C. canadensis songaricus*; c_xan - *C. canadensis xanthopygus*; e_A - *C. elaphus* haplogroup A; e_B - *C. elaphus* haplogroup B; e_C - *C. elaphus* haplogroup C; e_D - *C. elaphus* haplogroup D; e_E - *C. elaphus* haplogroup E; e_mac - *C. elaphus macneilli*; h - *C. hanglu yarkandensis*; n_cen - *C. nippon centralis*; n_hor - *C. nippon hortulorum*; n_kop - *C. nippon kopschi*; n_sic - *C. nippon sichuanicus*; n_tai - *C. nippon taiouanus*; n_yak - *C. nippon yakushimae*; n_yes - *C. nippon yesoensis*.

C. albirostris

C. canadensis

C. elaphus

C. hanglu

C. nippon

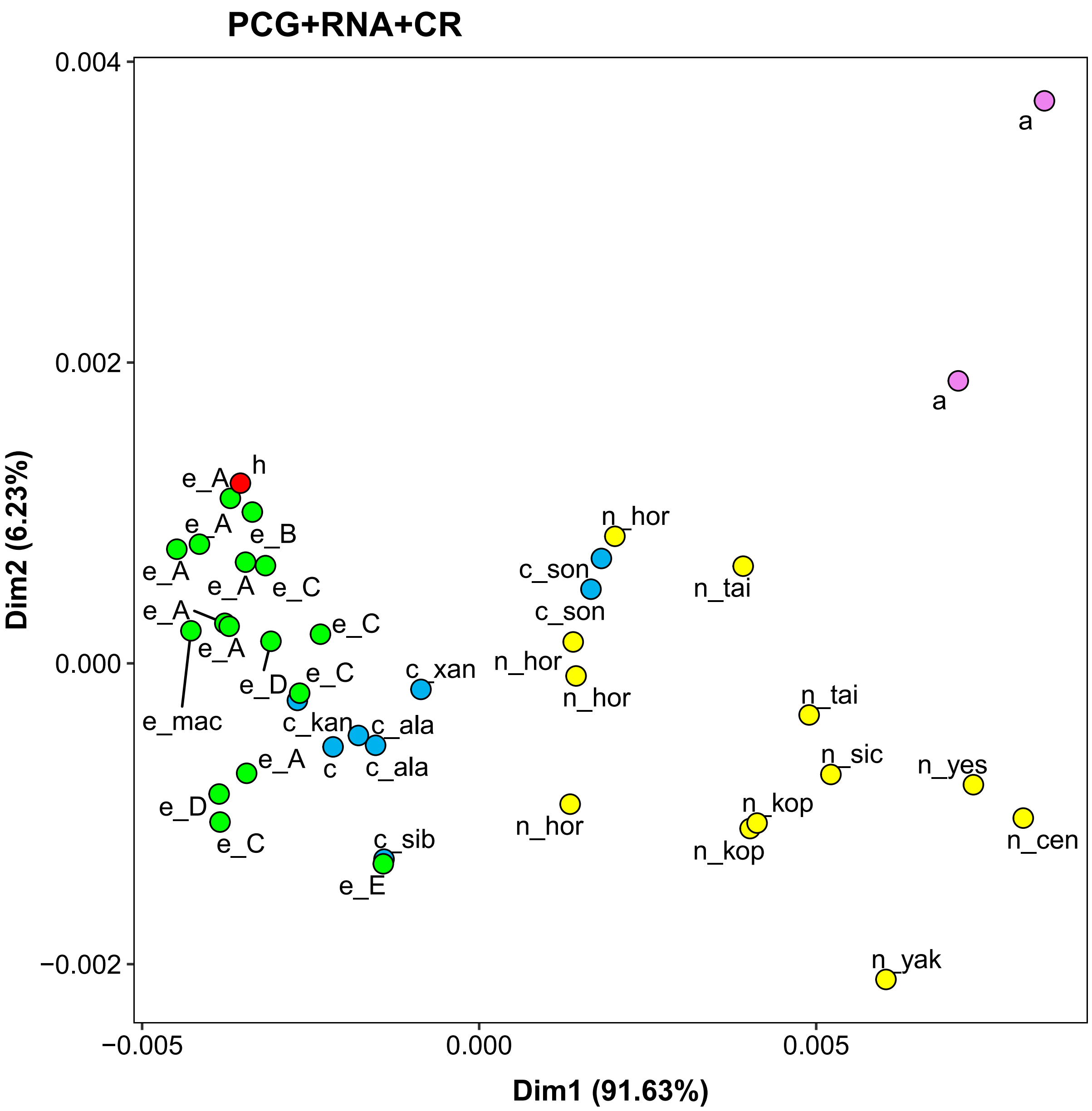
PCG+RNA+CR
Dim2 (6.23%)
Dim1 (91.63%)
0.004
0.002
0.000
−0.002
−0.005
0.000
0.005
a
a
h
e_A
e_A
e_B
e_A
e_A
e_C
e_A
e_C
e_A
e_D
e_C
e_mac
c_kan
c_ala
c
c_ala
e_A
e_D
e_C
c_sib
e_E
c_xan
c_son
c_son
n_hor
n_tai
n_hor
n_hor
n_tai
n_sic
n_yes
n_kop
n_kop
n_hor
n_cen
n_yak

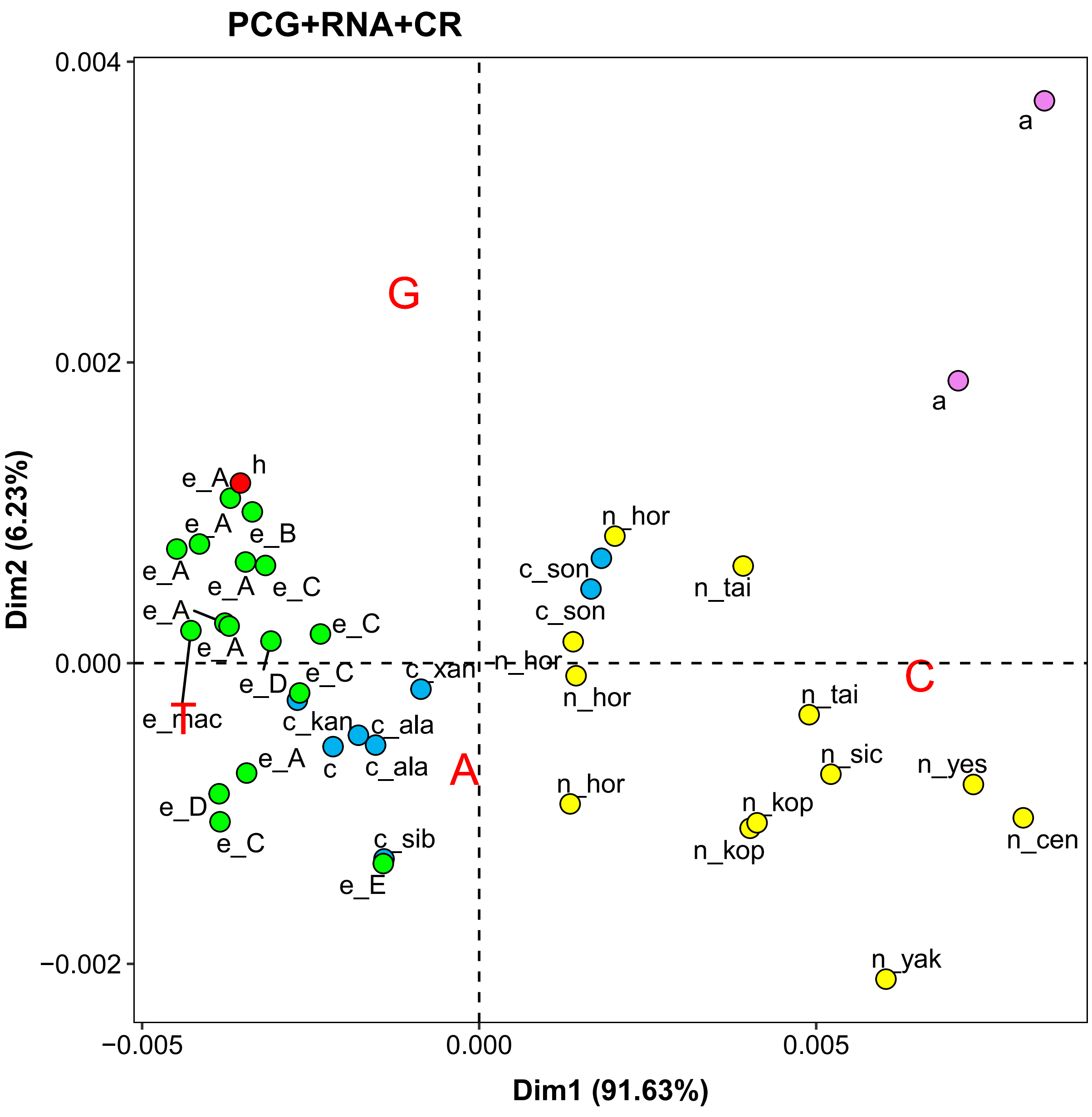

PCG+RNA+CR
Dim2 (6.23%)
Dim1 (91.63%)
0.004
0.002
0.000
−0.002
−0.005
0.000
0.005
G
A
C
T
a
a
h
e_A
e_A
e_A
e_A
e_A
e_A
e_A
e_B
e_C
e_C
e_C
e_C
e_D
e_D
e_E
e_mac
c_son
c_son
c_xan
c_kan
c_ala
c_ala
c
c_sib
n_hor
n_hor
n_hor
n_hor
n_tai
n_tai
n_sic
n_yes
n_kop
n_kop
n_cen
n_yak

ATP6

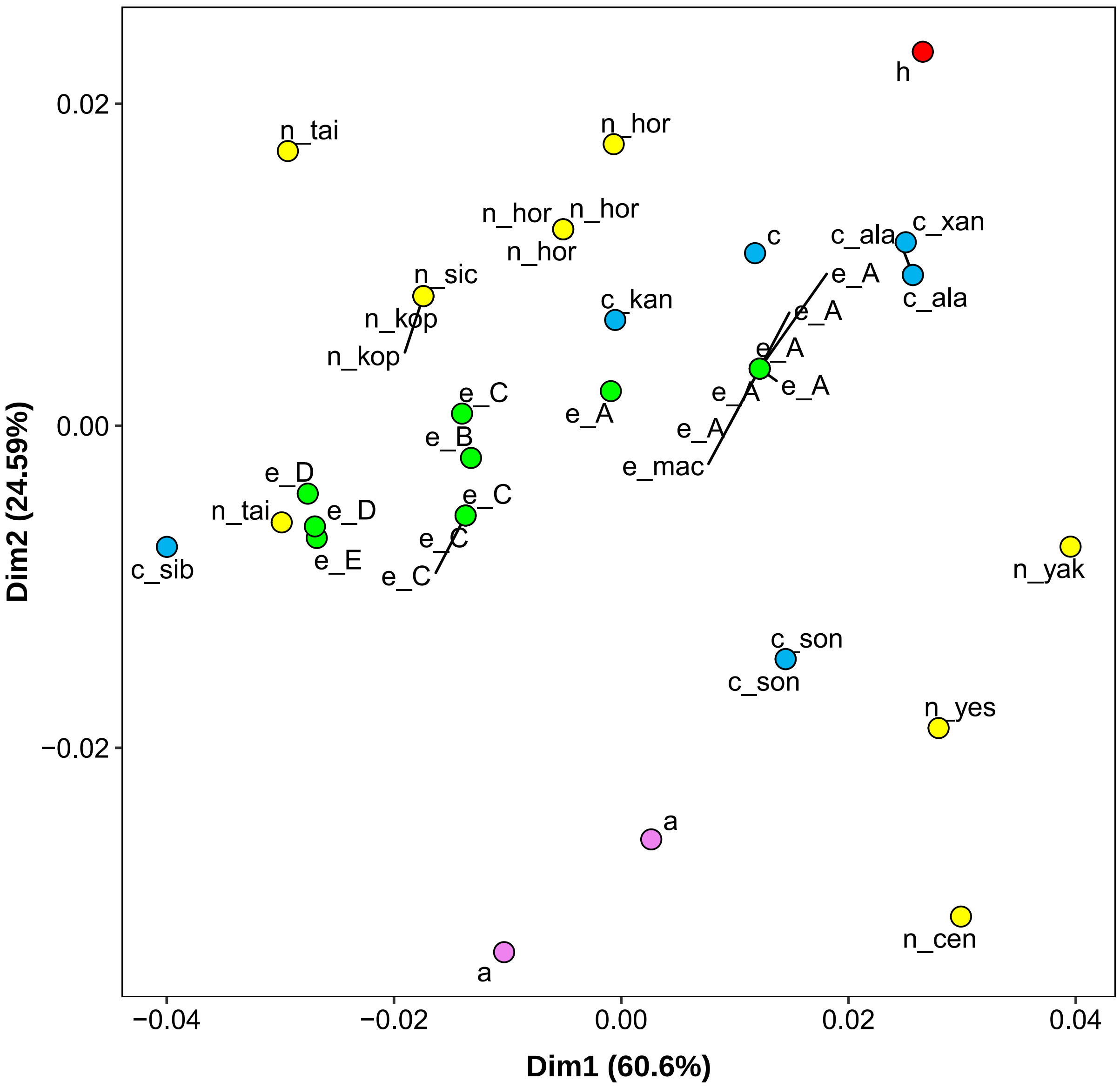

Dim1 (60.6%)
Dim2 (24.59%)
0.02
0.00
−0.02
−0.04
0.04
h
n_tai
n_hor
n_hor
n_hor
n_hor
c
c_ala
c_xan
c_ala
n_sic
n_kop
n_kop
c_kan
e_A
e_A
e_A
e_A
e_A
e_A
e_A
e_mac
e_C
e_B
e_D
e_D
e_E
n_tai
c_sib
e_C
e_C
e_C
n_yak
c_son
c_son
n_yes
a
a
n_cen

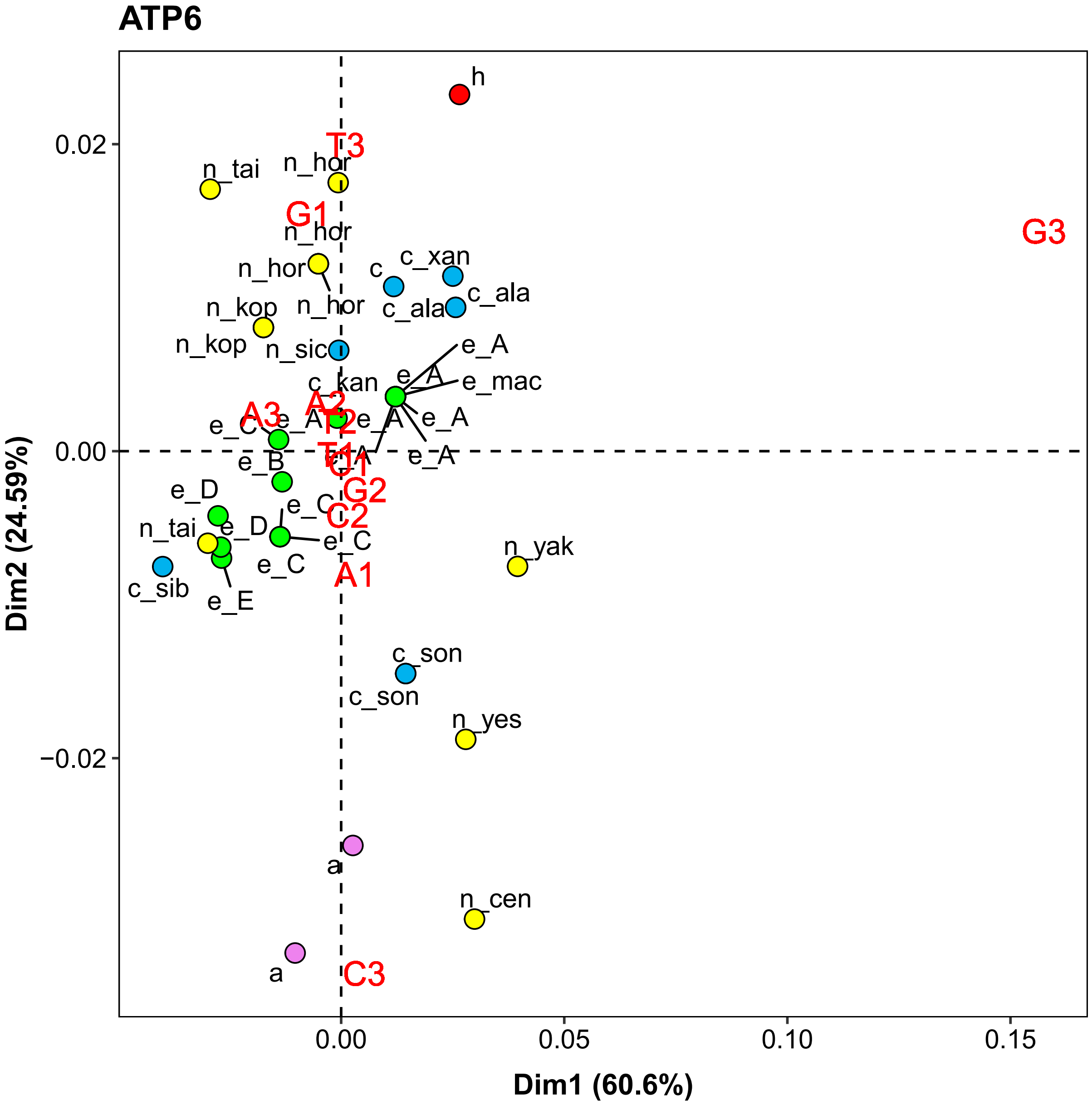

ATP6
Dim1 (60.6%)
Dim2 (24.59%)
0.02
0.00
−0.02
0.00
0.05
0.10
0.15
h
T3
n_hor
n_tai
G1
n_hor
G3
n_hor
c
c_xan
n_hor
c_ala
c_ala
n_kop
n_kop
n_sic
e_A
e_A
e_mac
c_kan
A2
A3
e_A
T2
e_A
e_A
e_C
T1
e_A
e_A
e_B
C1
G2
e_D
e_C
C2
n_tai
e_D
e_C
n_yak
e_C
A1
c_sib
e_E
c_son
c_son
n_yes
a
n_cen
a
C3

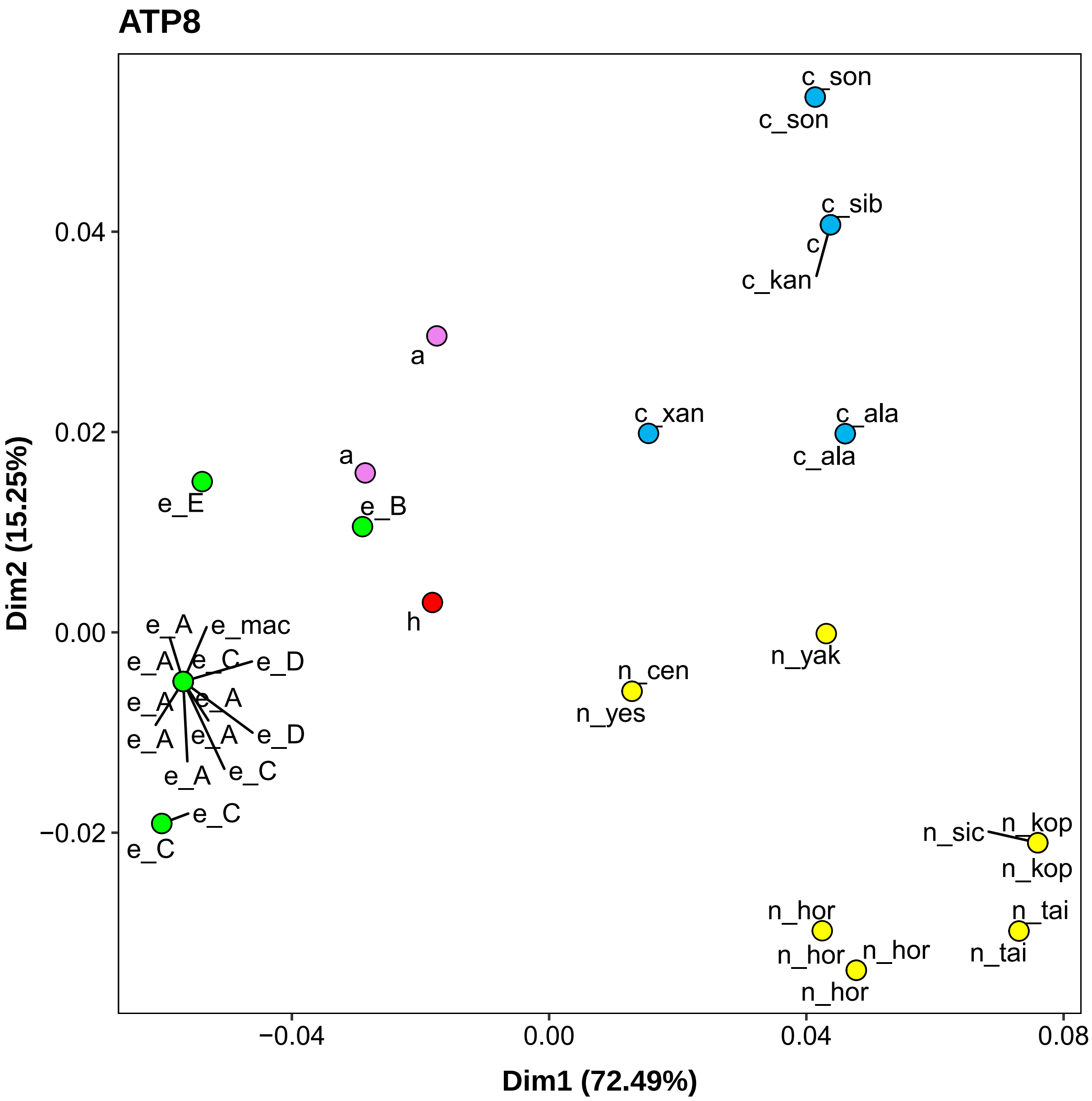
ATP8
Dim1 (72.49%)
Dim2 (15.25%)
−0.04
0.00
0.04
0.08
−0.02
0.00
0.02
0.04
c_son
c_son
c_sib
c
c_kan
a
c_xan
c_ala
c_ala
a
e_E
e_B
h
e_A
e_mac
e_A
e_C
e_D
e_A
e_A
e_A
e_A
e_D
e_A
e_C
e_C
e_C
n_yak
n_cen
n_yes
n_sic
n_kop
n_kop
n_hor
n_tai
n_hor
n_hor
n_tai
n_hor

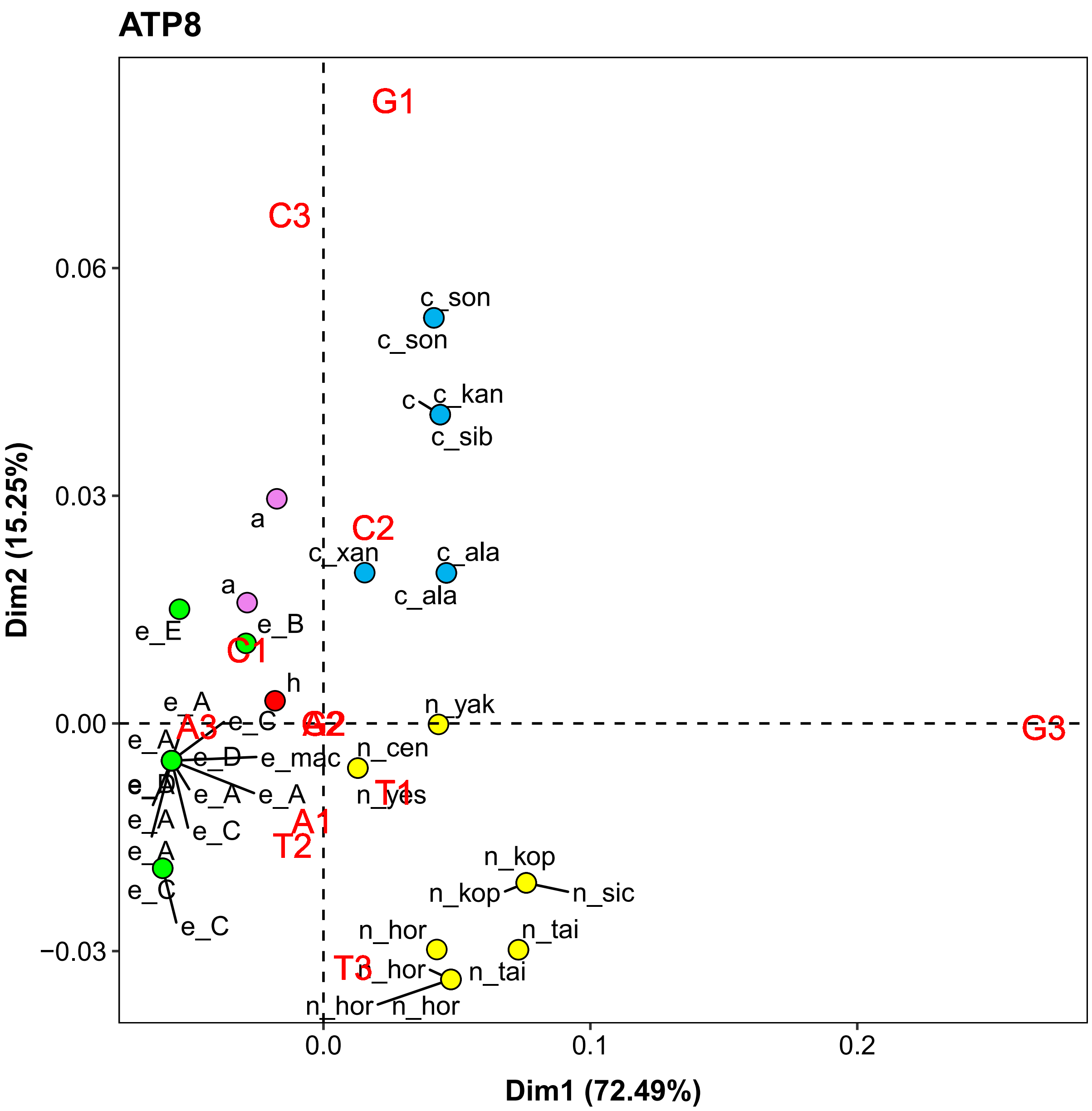
ATP8
Dim1 (72.49%)
Dim2 (15.25%)
0.06
0.03
0.00
−0.03
0.0
0.1
0.2
G1
C3
C2
C1
A3
G2
G3
T1
A1
T2
T3
c_son
c_son
c
c_kan
c_sib
c_xan
c_ala
c_ala
a
a
e_E
e_B
h
e_A
e_C
e_A
e_D
e_mac
e_A
e_A
e_A
e_C
e_A
e_C
e_C
n_yak
n_cen
n_yes
n_kop
n_kop
n_sic
n_hor
n_tai
n_hor
n_tai
n_hor
n_hor

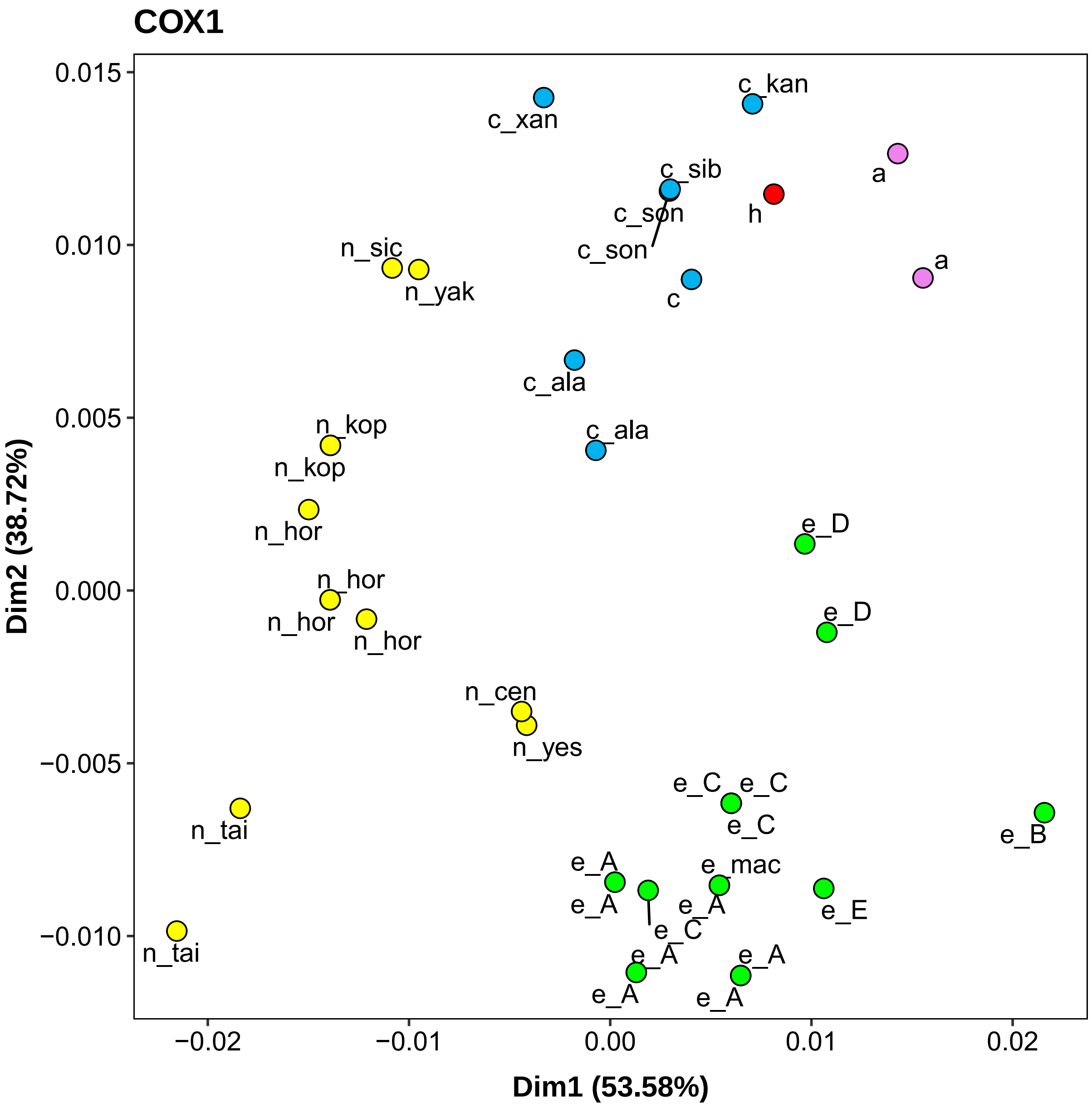
COX1
Dim1 (53.58%)
Dim2 (38.72%)
0.015
0.010
0.005
0.000
−0.005
−0.010
−0.02
−0.01
0.00
0.01
0.02
c_xan
c_kan
c_sib
c_son
c_son
c
c_ala
c_ala
h
a
a
n_sic
n_yak
n_kop
n_kop
n_hor
n_hor
n_hor
n_hor
n_cen
n_yes
n_tai
n_tai
e_D
e_D
e_C
e_C
e_C
e_B
e_A
e_A
e_mac
e_A
e_E
e_C
e_A
e_A
e_A
e_A

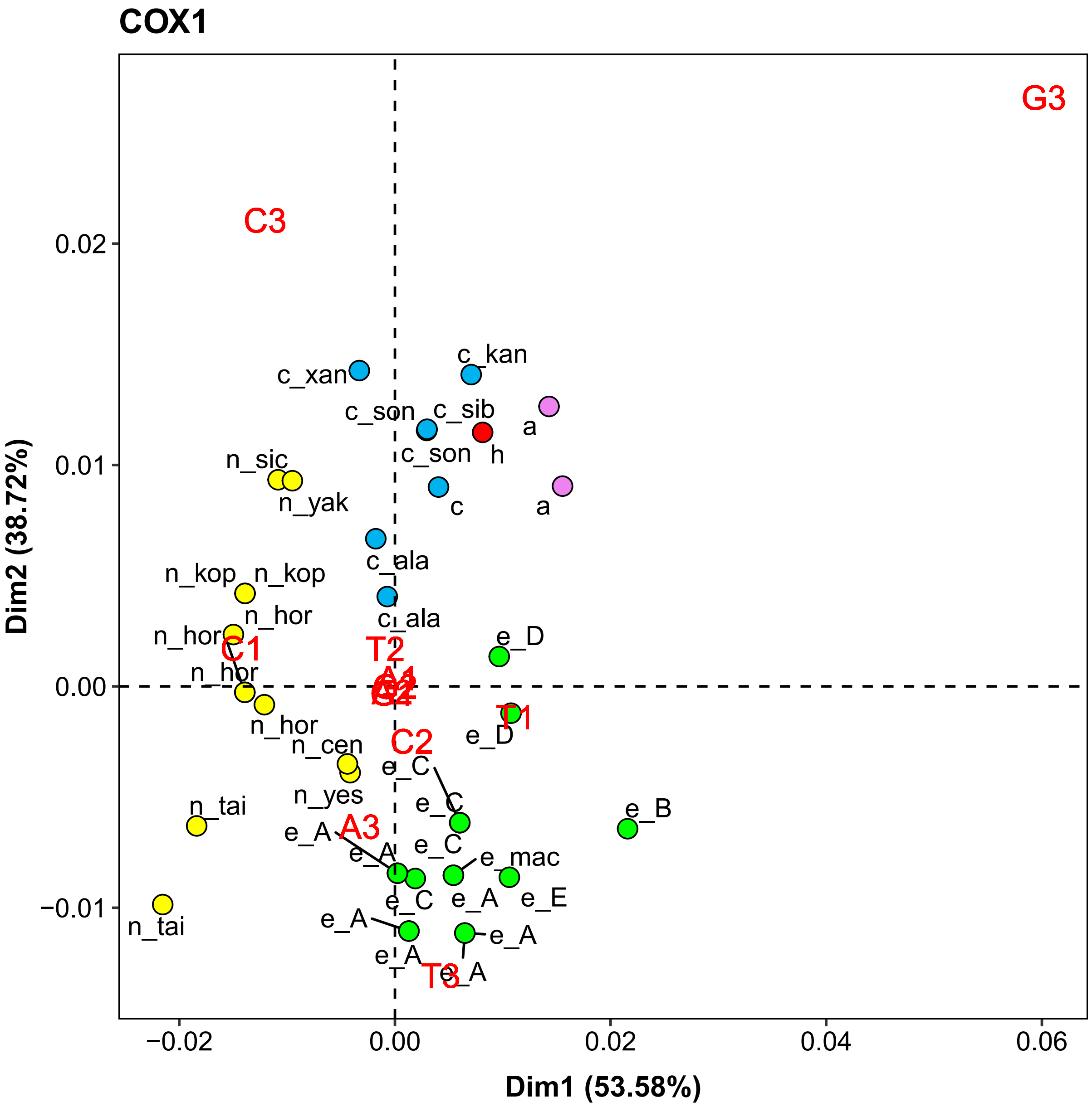

COX1
Dim1 (53.58%)
Dim2 (38.72%)
−0.02
0.00
0.02
0.04
0.06
−0.01
0.00
0.01
0.02
G3
C3
C1
T2
C2
T1
A3
T3
c_xan
c_kan
c_son
c_sib
c_son
c
c_ala
c_ala
a
a
h
n_sic
n_yak
n_kop
n_kop
n_hor
n_hor
n_hor
n_hor
n_cen
n_yes
n_tai
n_tai
e_D
e_D
e_C
e_C
e_C
e_C
e_B
e_mac
e_E
e_A
e_A
e_A
e_A
e_A
e_A
e_A

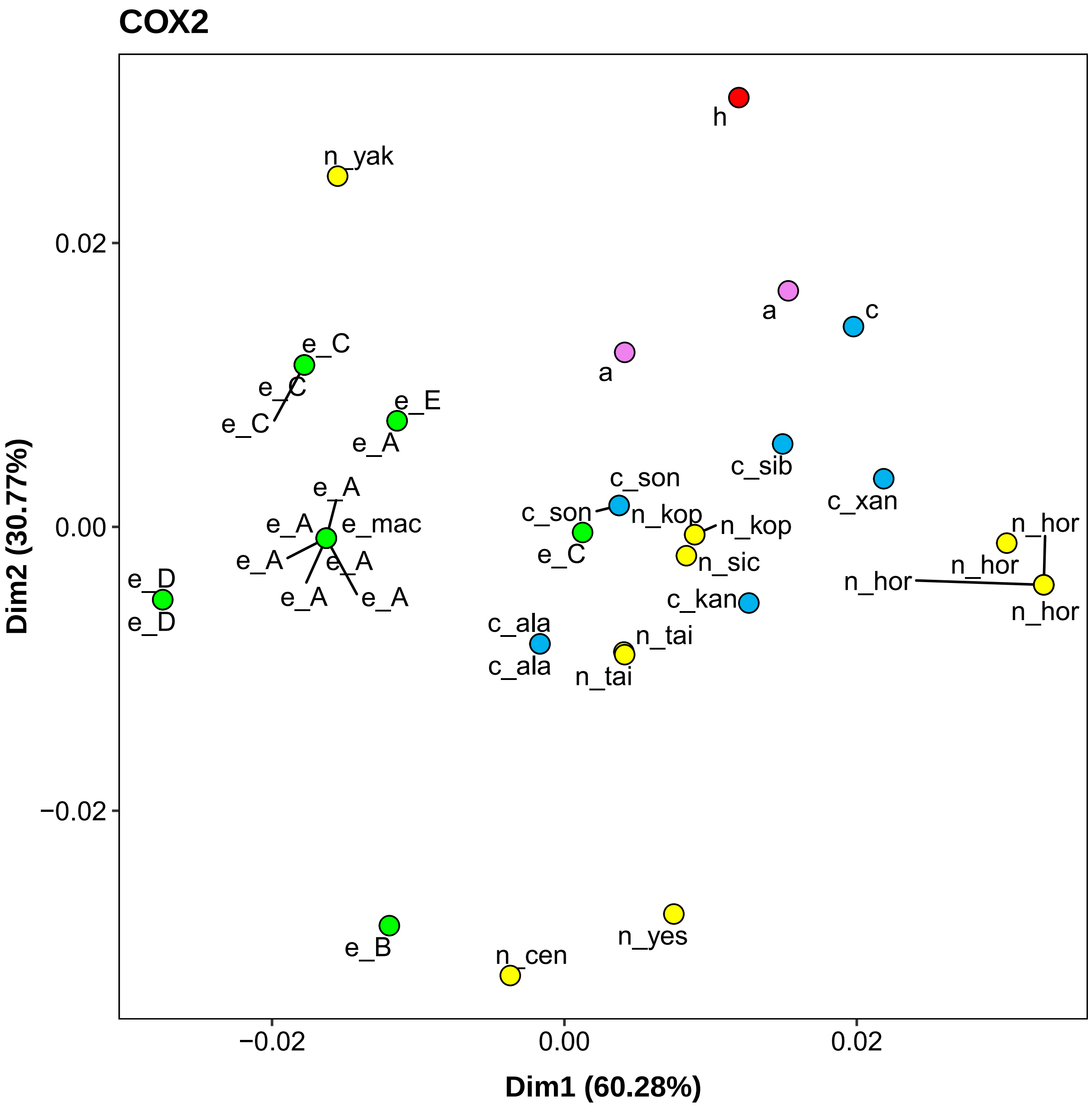
COX2
Dim1 (60.28%)
Dim2 (30.77%)
0.02
0.00
−0.02
−0.02
0.00
0.02
h
n_yak
a
c
a
e_C
e_C
e_C
e_E
e_A
c_sib
c_xan
c_son
e_A
c_son
n_kop
n_kop
e_A
e_mac
e_C
n_sic
n_hor
e_A
e_A
n_hor
e_D
n_hor
e_A
e_A
c_kan
n_hor
e_D
c_ala
n_tai
c_ala
n_tai
n_yes
e_B
n_cen

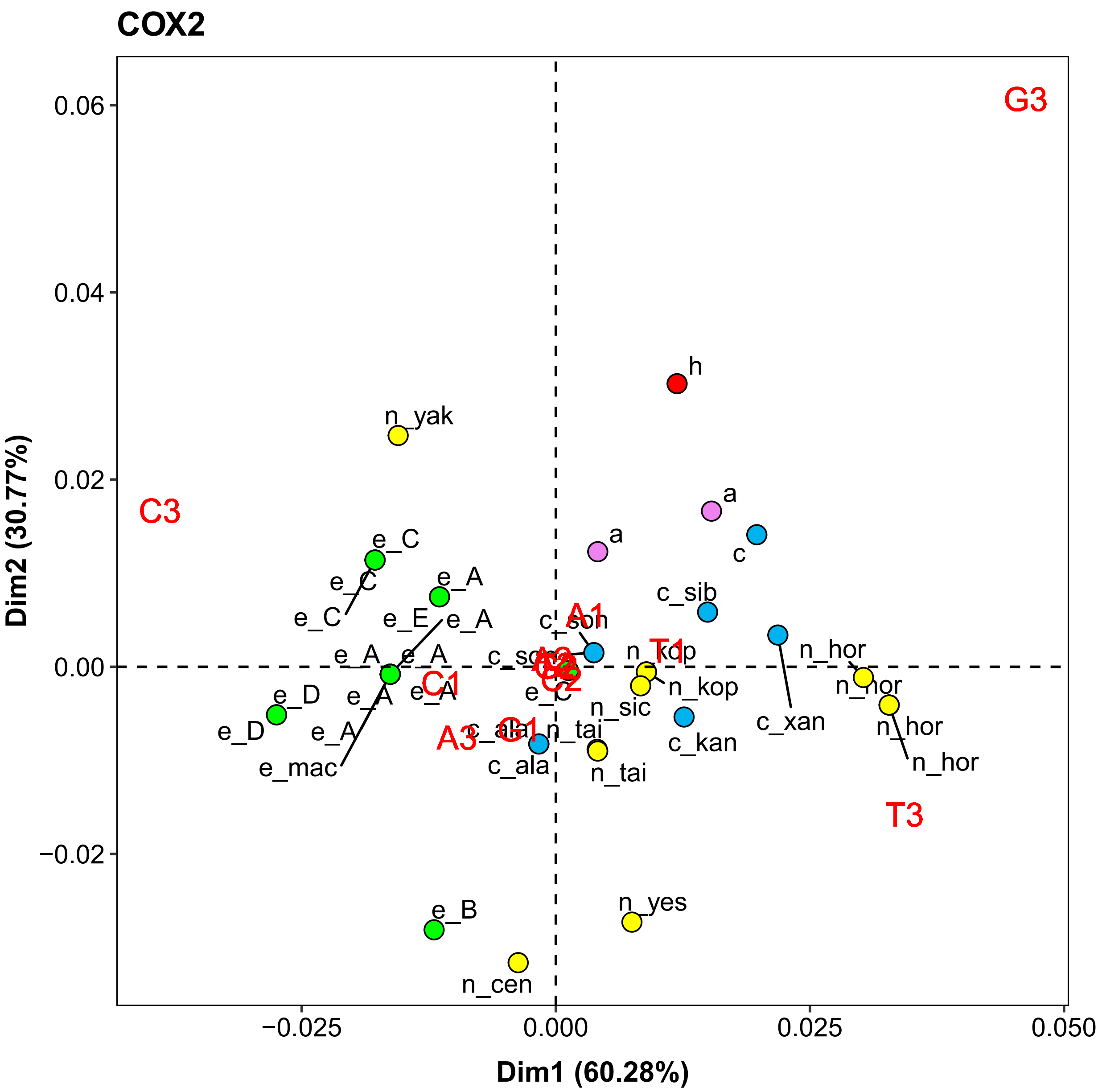

COX2
G3
C3
h
n_yak
a
a
c
e_C
e_C
e_C
e_A
e_A
e_E
c_sib
A1
c_son
T1
n_kop
n_kop
n_hor
n_hor
n_hor
n_hor
e_A
e_A
c_son
C1
e_A
e_A
e_D
e_D
e_A
e_C
C2
n_sic
c_kan
c_xan
A3
c_ala
G1
n_tai
n_tai
c_ala
e_mac
T3
e_B
n_yes
n_cen
Dim2 (30.77%)
Dim1 (60.28%)
0.06
0.04
0.02
0.00
−0.02
−0.025
0.000
0.025
0.050

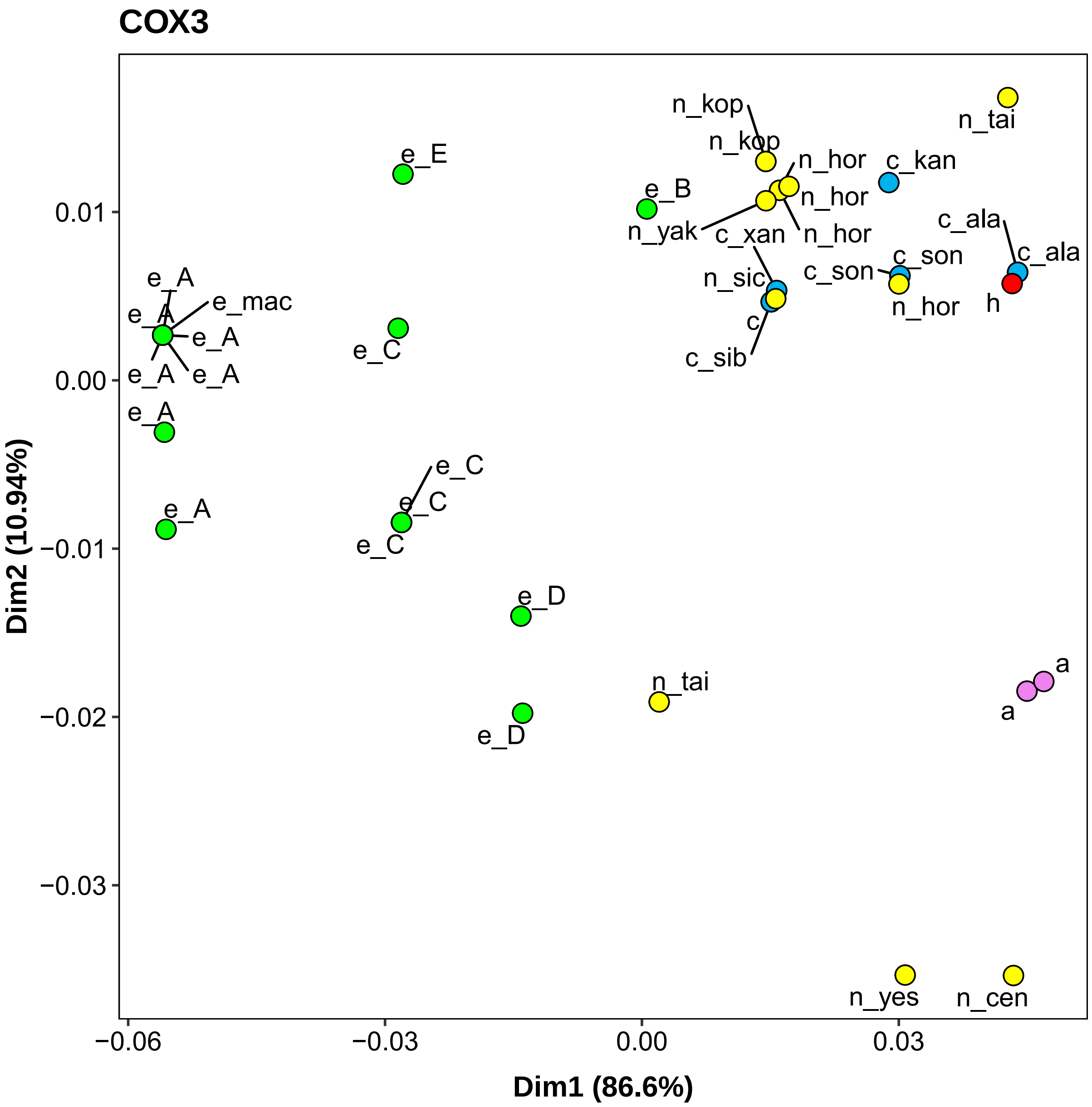
COX3
Dim1 (86.6%)
Dim2 (10.94%)
−0.06
−0.03
0.00
0.03
0.01
0.00
−0.01
−0.02
−0.03
e_E
e_B
e_A
e_mac
e_C
e_D
n_kop
n_hor
n_yak
n_tai
n_sic
n_yes
n_cen
c_kan
c_ala
c_son
c_xan
c_sib
c
h
a

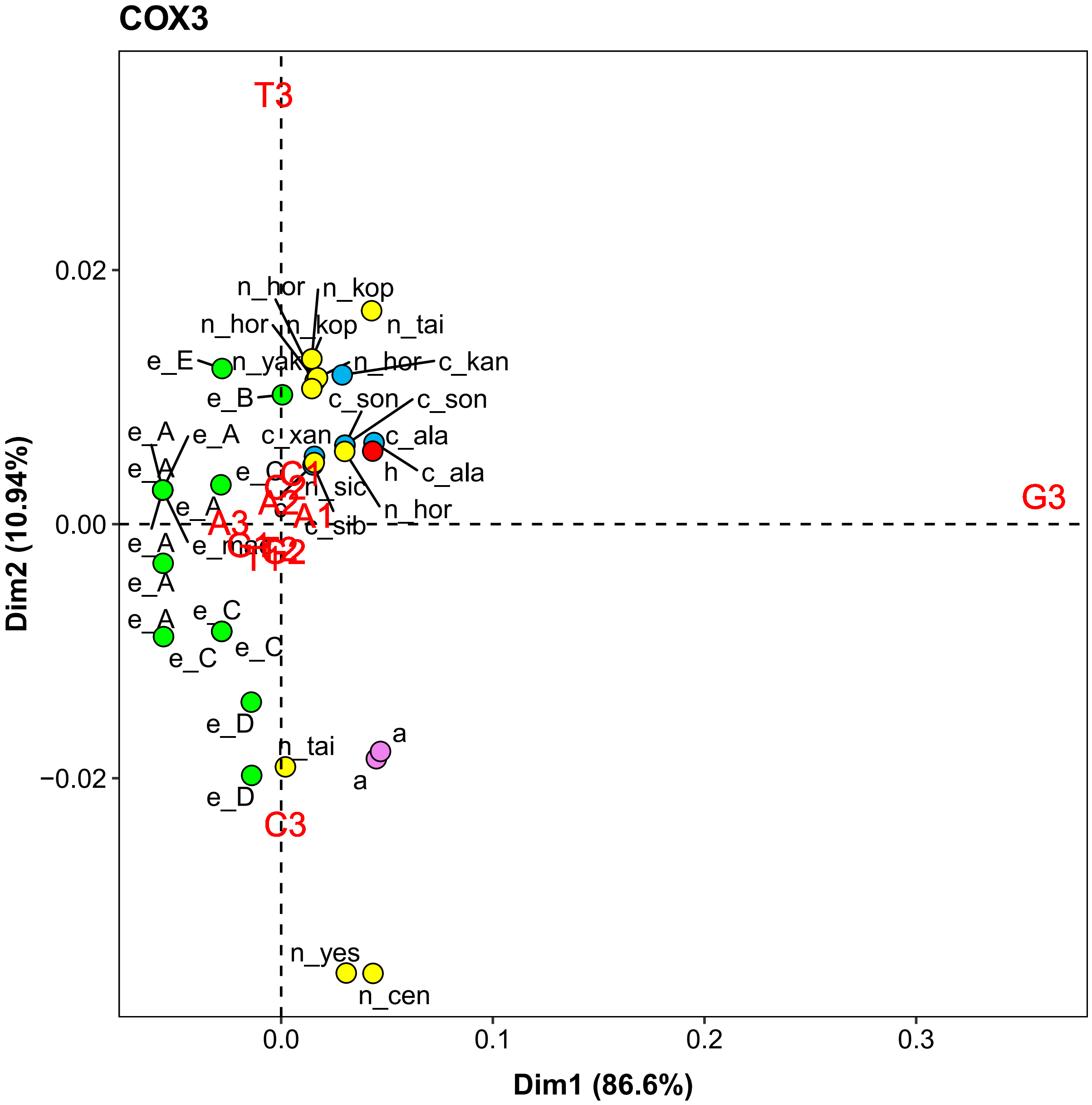
COX3
Dim1 (86.6%)
Dim2 (10.94%)
0.02
0.00
−0.02
0.0
0.1
0.2
0.3
T3
G3
C3
n_hor
n_kop
n_tai
e_E
n_yak
c_kan
e_B
c_son
c_xan
c_ala
h
n_hor
e_A
e_C
n_sic
c_sib
e_D
n_tai
a
n_yes
n_cen

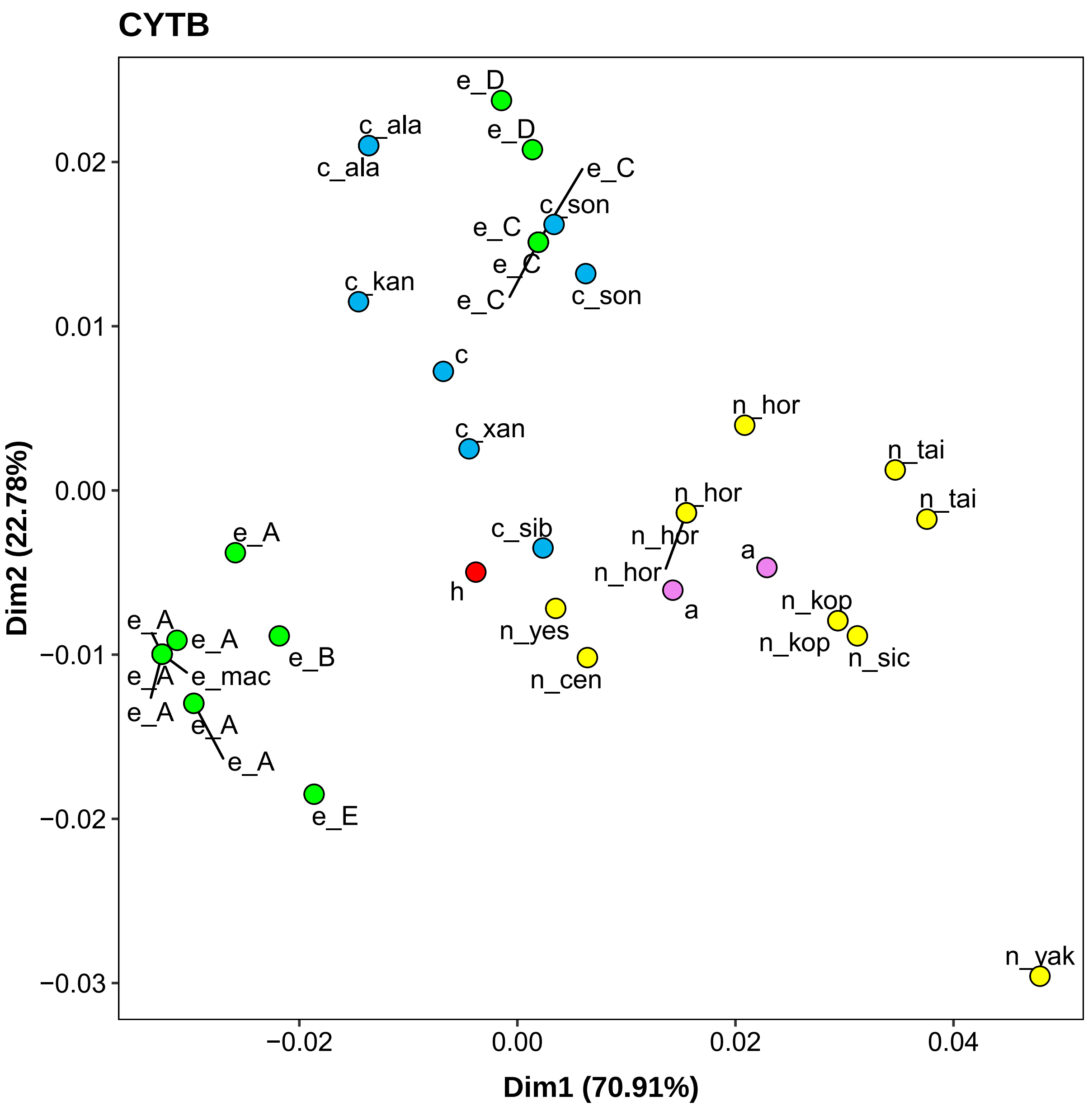

CYTB
Dim1 (70.91%)
Dim2 (22.78%)
−0.02
0.00
0.02
0.04
0.02
0.01
0.00
−0.01
−0.02
−0.03
e_D
e_D
c_ala
c_ala
e_C
c_son
e_C
e_C
e_C
c_son
c_kan
c
c_xan
n_hor
n_tai
n_tai
n_hor
n_hor
n_hor
e_A
c_sib
a
a
h
n_yes
n_kop
n_kop
n_sic
n_cen
e_A
e_A
e_mac
e_B
e_A
e_A
e_A
e_A
e_E
n_yak

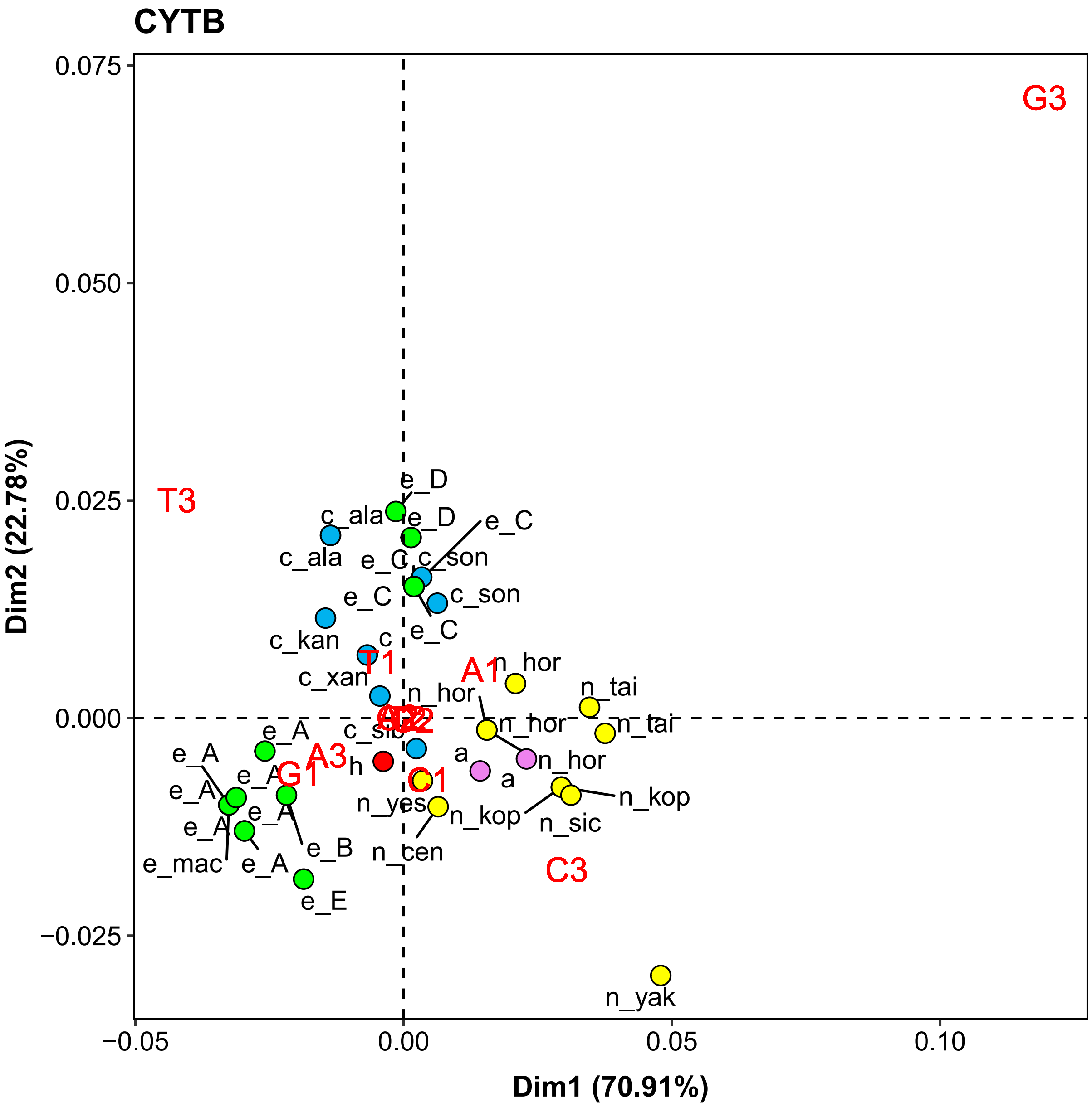
CYTB
Dim2 (22.78%)
Dim1 (70.91%)
0.075
0.050
0.025
0.000
−0.025
−0.05
0.00
0.05
0.10
G3
T3
T1
A1
A3
G1
C1
C3
e_D
e_D
e_C
c_ala
c_ala
e_C
c_son
c_son
e_C
e_C
c_kan
c
c_xan
n_hor
n_hor
n_hor
n_hor
n_tai
n_tai
c_sib
e_A
e_A
e_A
e_A
e_A
e_A
e_A
e_mac
e_B
e_E
h
a
a
n_yes
n_kop
n_kop
n_sic
n_cen
n_yak

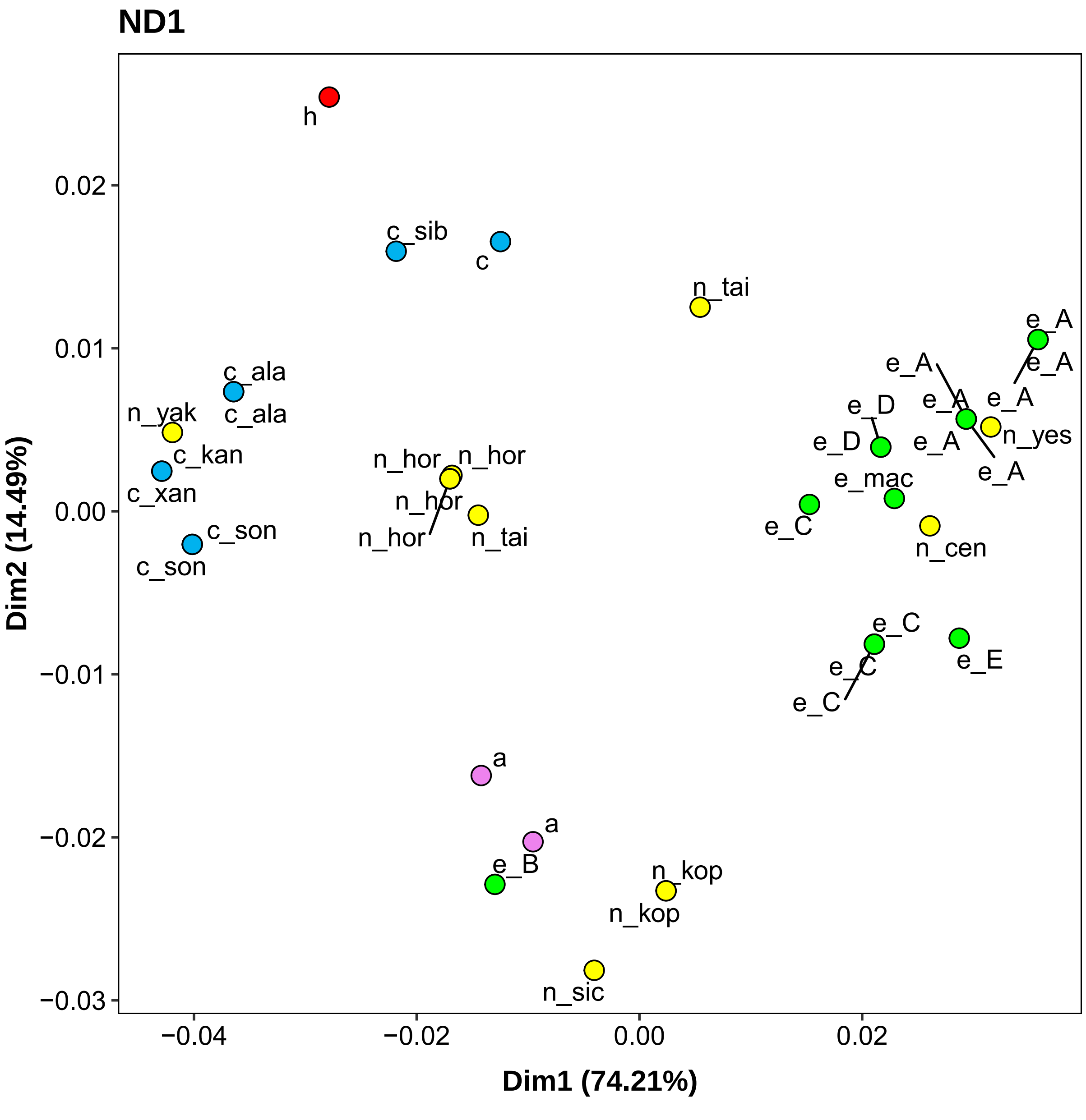

ND1
Dim1 (74.21%)
Dim2 (14.49%)
−0.04
−0.02
0.00
0.02
−0.03
−0.02
−0.01
0.00
0.01
0.02
h
c_sib
c
n_tai
c_ala
c_ala
n_yak
c_kan
c_xan
c_son
c_son
n_hor
n_hor
n_hor
n_hor
n_tai
e_A
e_A
e_A
e_A
e_A
e_A
e_A
e_D
e_D
n_yes
e_mac
e_C
n_cen
e_C
e_C
e_C
e_E
a
a
e_B
n_kop
n_kop
n_sic

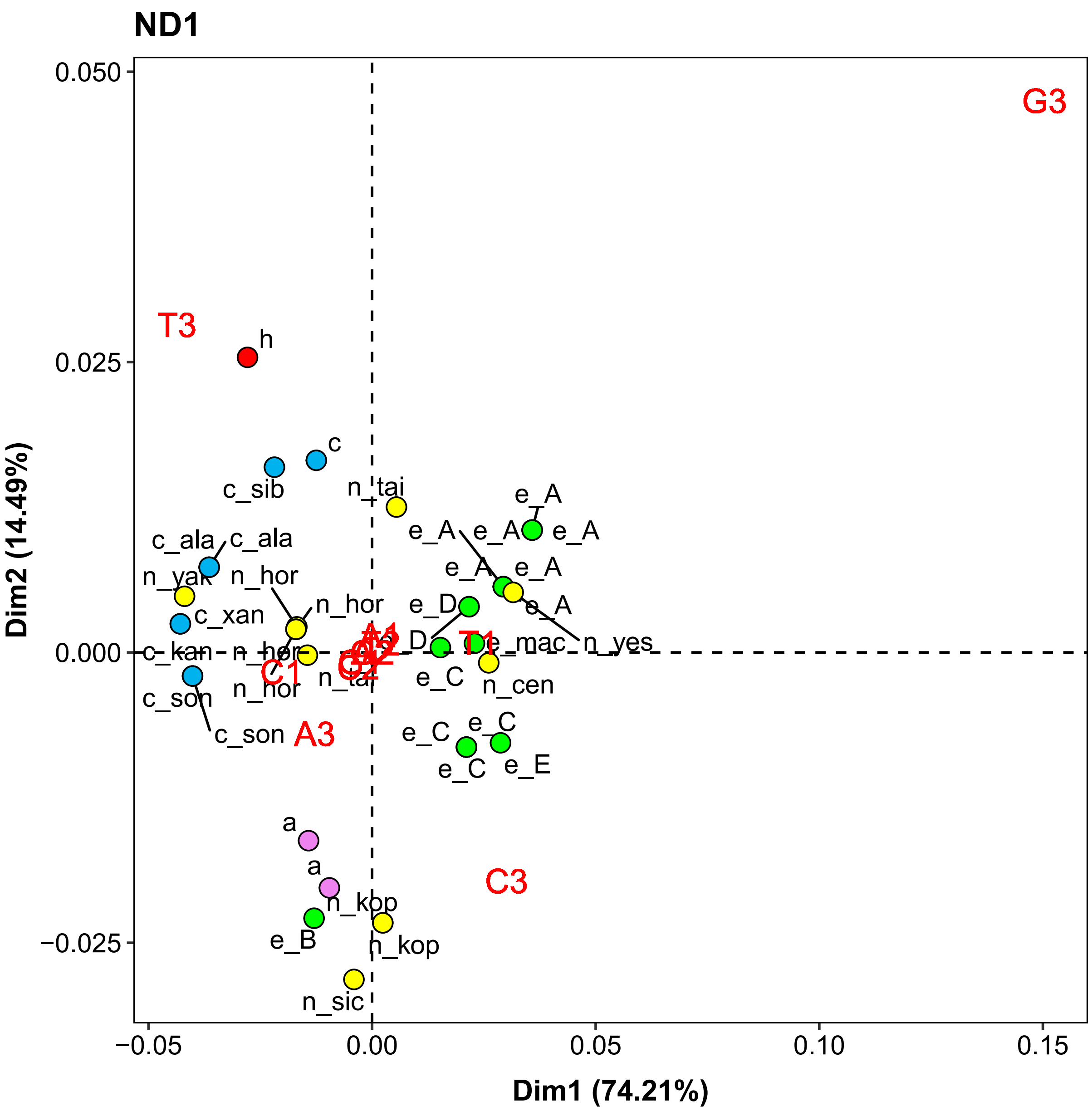
ND1
Dim1 (74.21%)
Dim2 (14.49%)
0.050
0.025
0.000
−0.025
−0.05
0.00
0.05
0.10
0.15
G3
T3
T1
C1
A3
C3
h
c
c_sib
n_tai
e_A
c_ala
n_yak
n_hor
e_D
c_xan
c_kan
e_mac
n_yes
n_cen
e_C
c_son
e_E
a
n_kop
e_B
n_sic

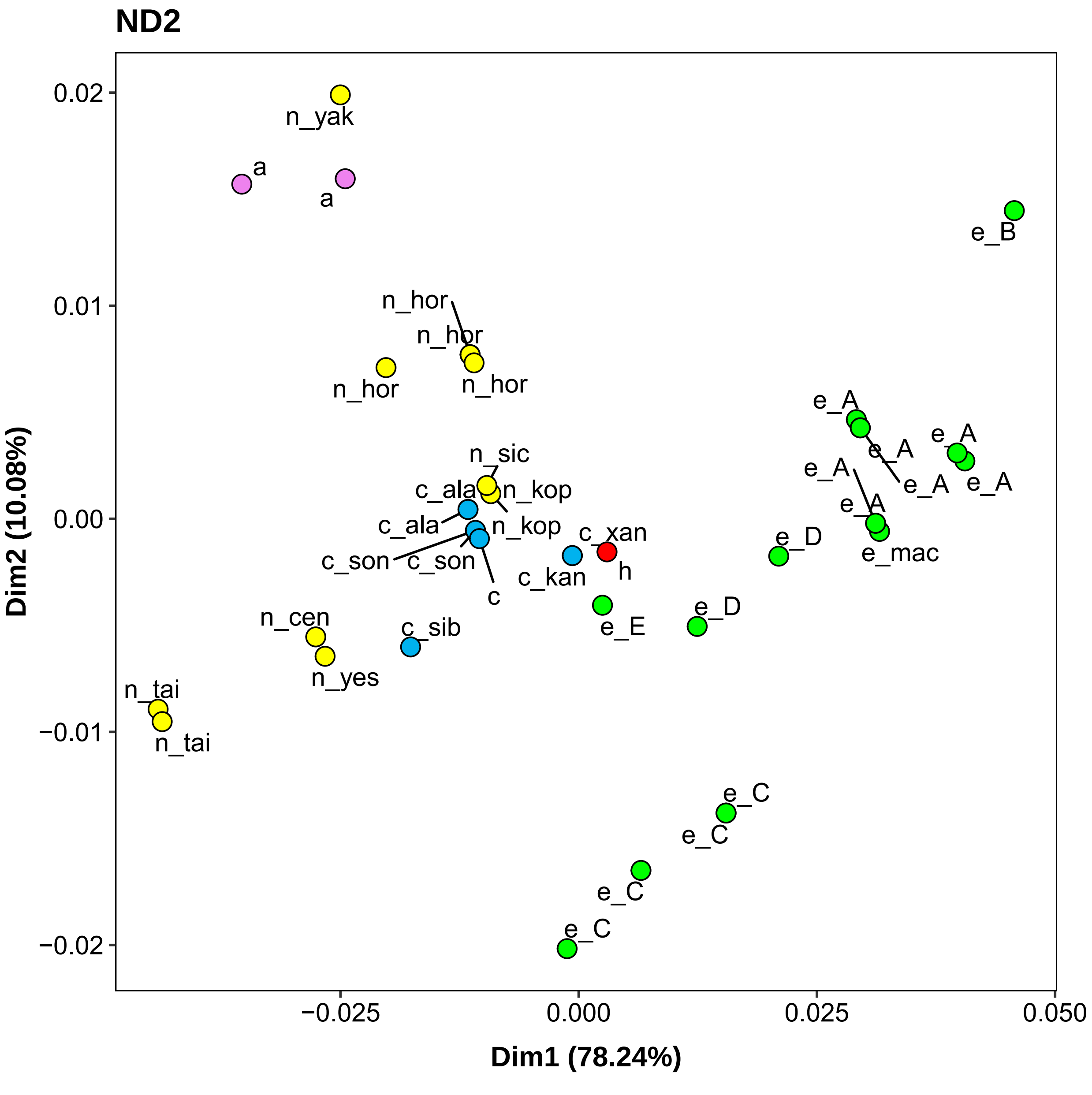
ND2
Dim2 (10.08%)
Dim1 (78.24%)
0.02
0.01
0.00
−0.01
−0.02
−0.025
0.000
0.025
0.050
n_yak
a
a
e_B
n_hor
n_hor
n_hor
n_hor
n_sic
c_ala
n_kop
c_ala
n_kop
c_son
c_son
c
c_xan
h
c_kan
e_E
e_D
e_D
e_A
e_A
e_A
e_A
e_A
e_A
e_mac
n_cen
n_yes
c_sib
n_tai
n_tai
e_C
e_C
e_C
e_C

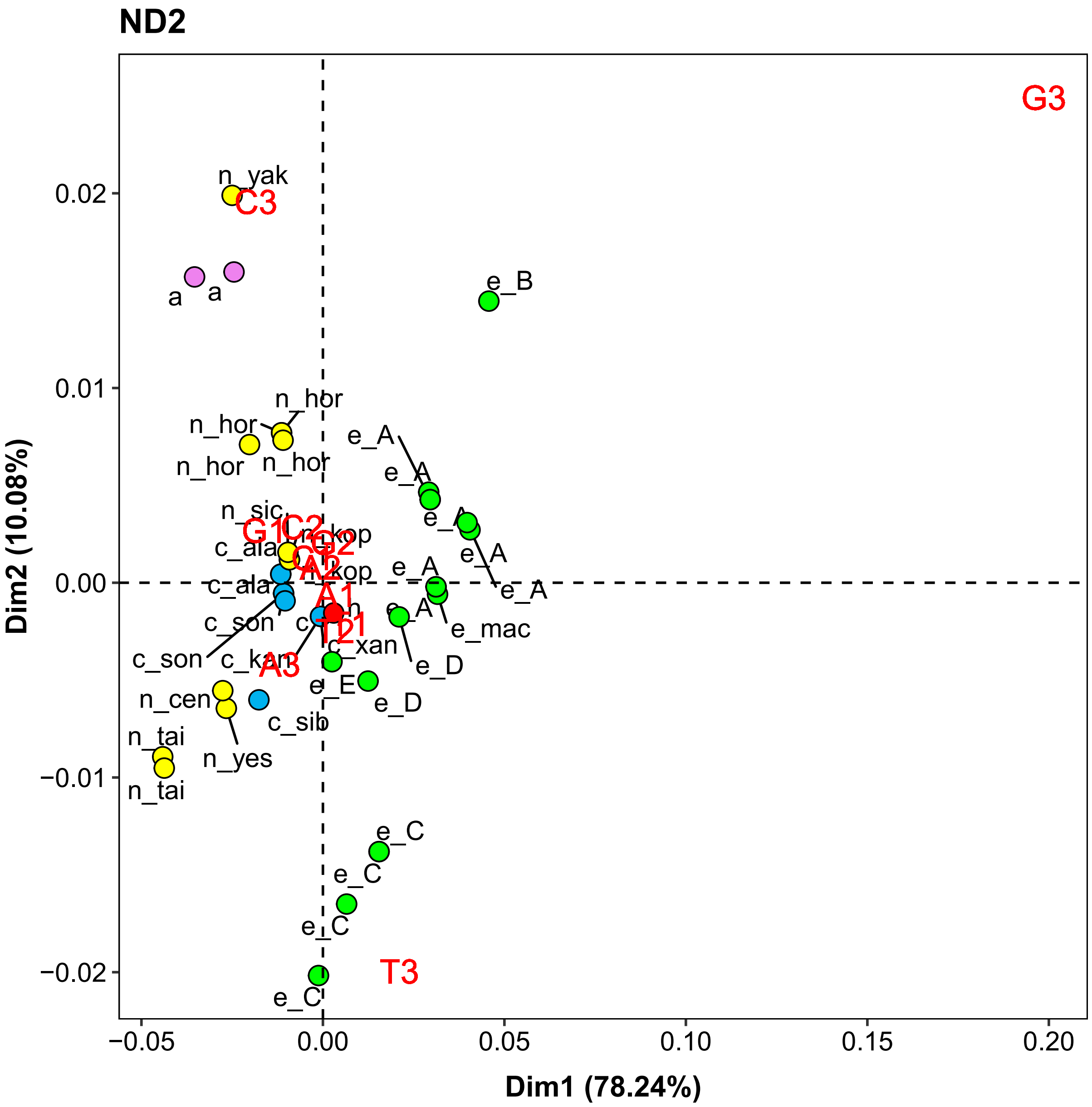
ND2
G3
n_yak
C3
a
a
e_B
n_hor
n_hor
n_hor
n_hor
e_A
e_A
e_A
e_A
e_A
e_A
e_A
e_mac
e_D
e_D
e_E
n_sic
G1C2
n_kop
c_ala
G2
A2
n_kop
c_ala
A1
c_son
c_son
T2
c_xan
c_kar
A3
n_cen
c_sib
n_tai
n_yes
n_tai
e_C
e_C
e_C
e_C
T3
0.02
0.01
0.00
−0.01
−0.02
−0.05
0.00
0.05
0.10
0.15
0.20
Dim1 (78.24%)
Dim2 (10.08%)

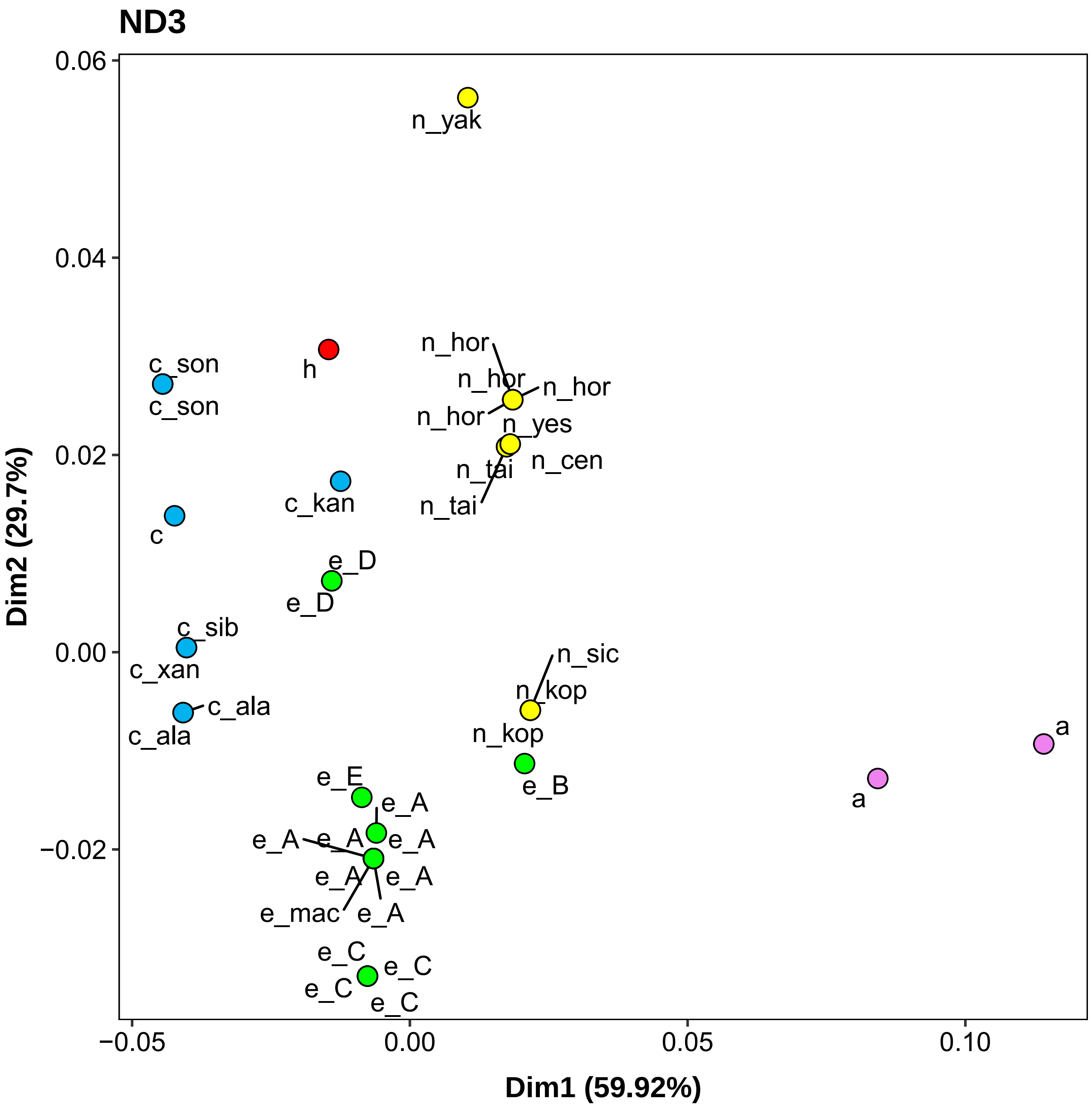

ND3
0.06
0.04
0.02
0.00
−0.02
−0.05
0.00
0.05
0.10
Dim1 (59.92%)
Dim2 (29.7%)
n_yak
n_hor
n_hor
n_hor
n_hor
n_yes
n_tai
n_cen
n_tai
c_son
c_son
h
c_kan
c
e_D
e_D
c_sib
c_xan
c_ala
c_ala
n_sic
n_kop
n_kop
e_B
a
a
e_E
e_A
e_A
e_A
e_A
e_A
e_A
e_A
e_mac
e_C
e_C
e_C
e_C

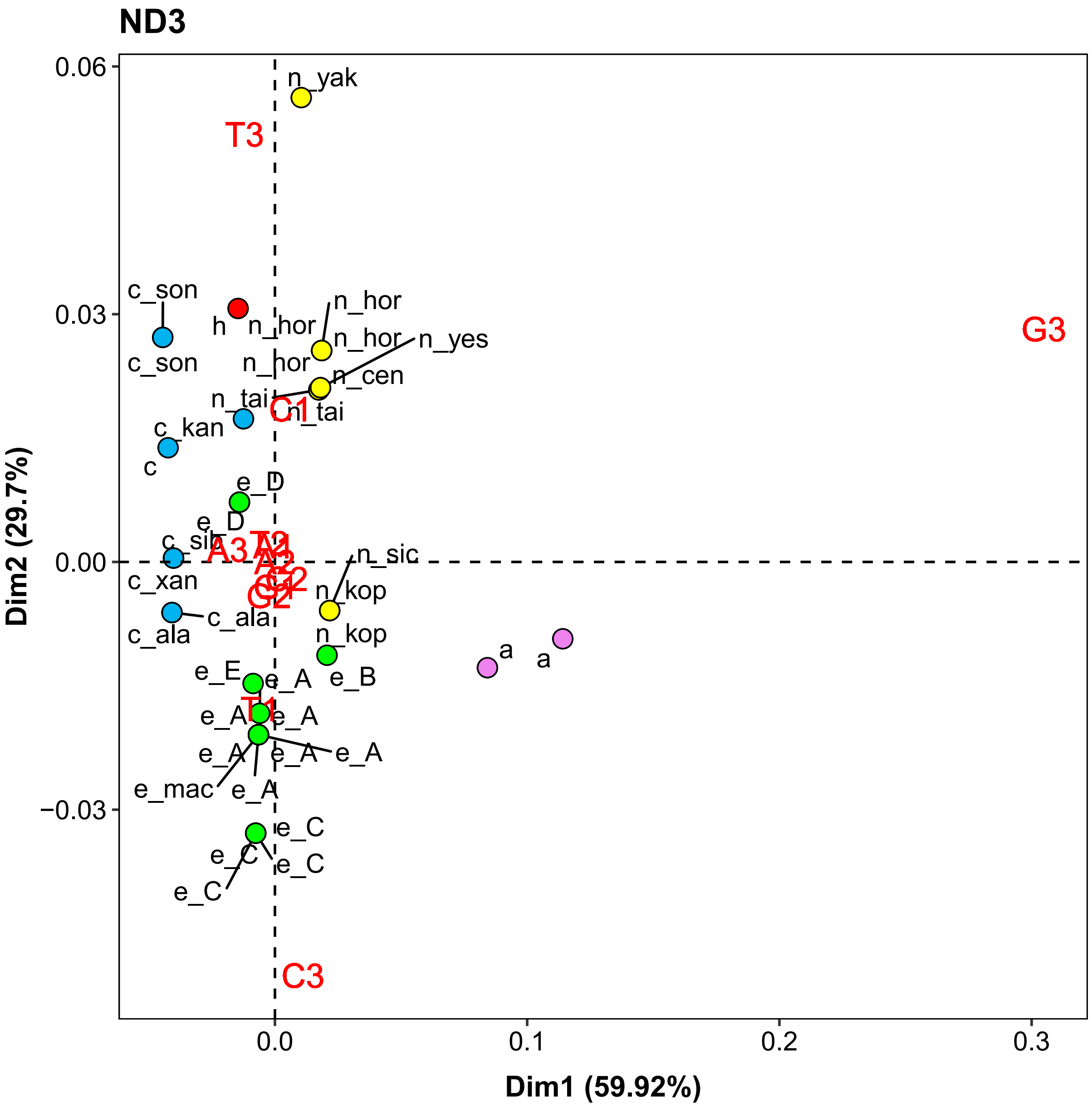

ND3
Dim1 (59.92%)
Dim2 (29.7%)
0.06
0.03
0.00
−0.03
0.0
0.1
0.2
0.3
n_yak
T3
G3
c_son
h
n_hor
n_yes
n_cen
n_tai
C1
c_kan
c
e_D
c_sih
A3
n_sic
c_xan
n_kop
c_ala
a
e_E
e_A
e_B
T1
e_mac
e_C
C3

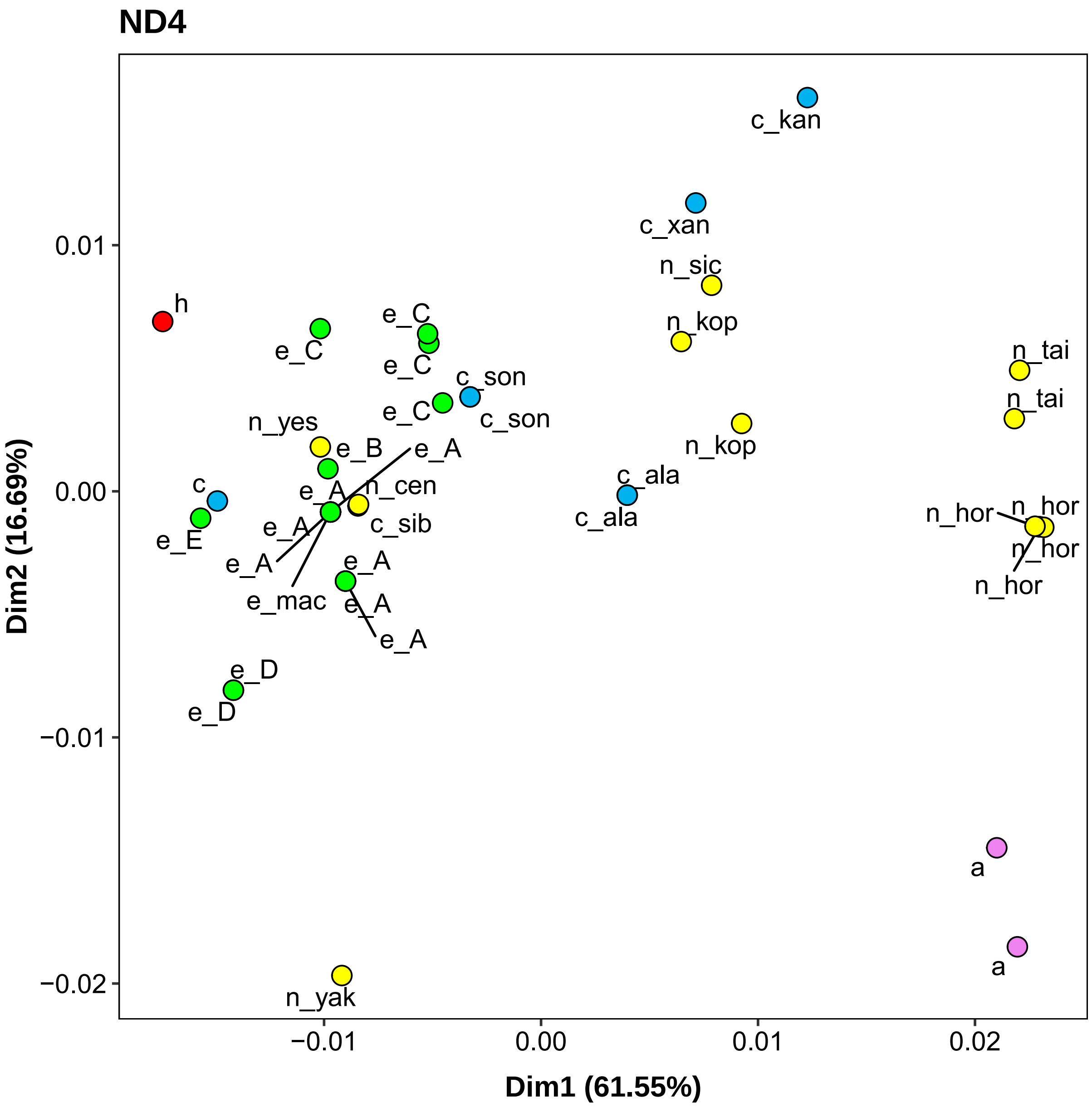

ND4
Dim1 (61.55%)
Dim2 (16.69%)
0.01
0.00
−0.01
−0.02
−0.01
0.00
0.01
0.02
c_kan
c_xan
n_sic
n_kop
n_tai
n_tai
h
e_C
e_C
e_C
c_son
c_son
e_C
n_yes
e_B
e_A
n_kop
c_ala
c_ala
c
e_A
n_cen
c_sib
e_E
e_A
e_A
e_mac
e_A
e_A
e_A
n_hor
n_hor
n_hor
n_hor
e_D
e_D
a
a
n_yak

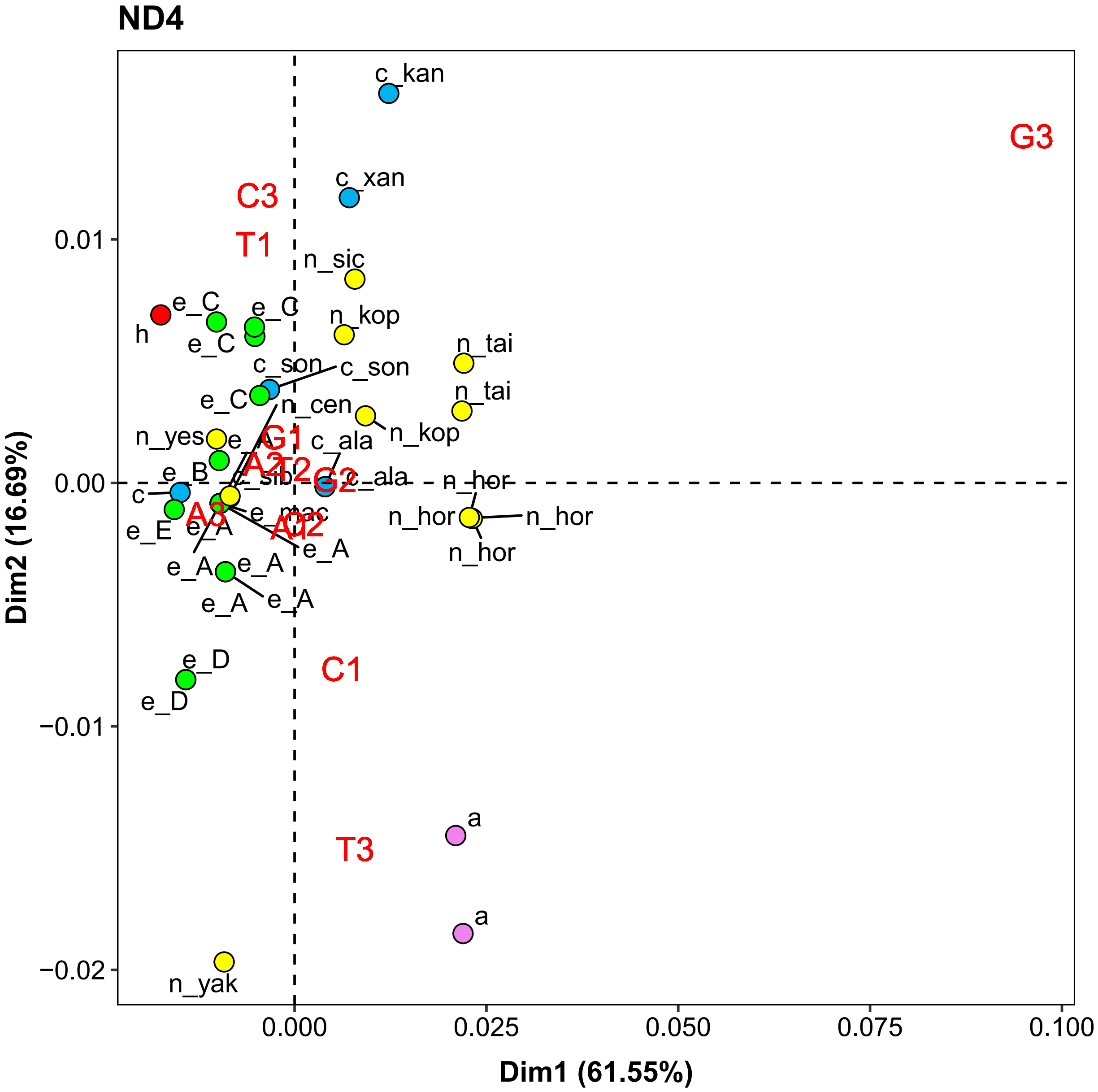
ND4
Dim1 (61.55%)
Dim2 (16.69%)
0.000
0.025
0.050
0.075
0.100
0.01
0.00
−0.01
−0.02
G3
C3
T1
G1
A2
T2
G2
A3
A1
C2
C1
T3
c_kan
c_xan
n_sic
e_C
e_C
e_C
e_C
h
n_kop
n_tai
n_tai
c_son
c_son
n_cen
n_kop
n_yes
c_ala
c_ala
e_B
c
e_E
e_A
e_A
e_A
e_A
e_A
e_A
e_mac
n_hor
n_hor
n_hor
n_hor
e_D
e_D
a
a
n_yak

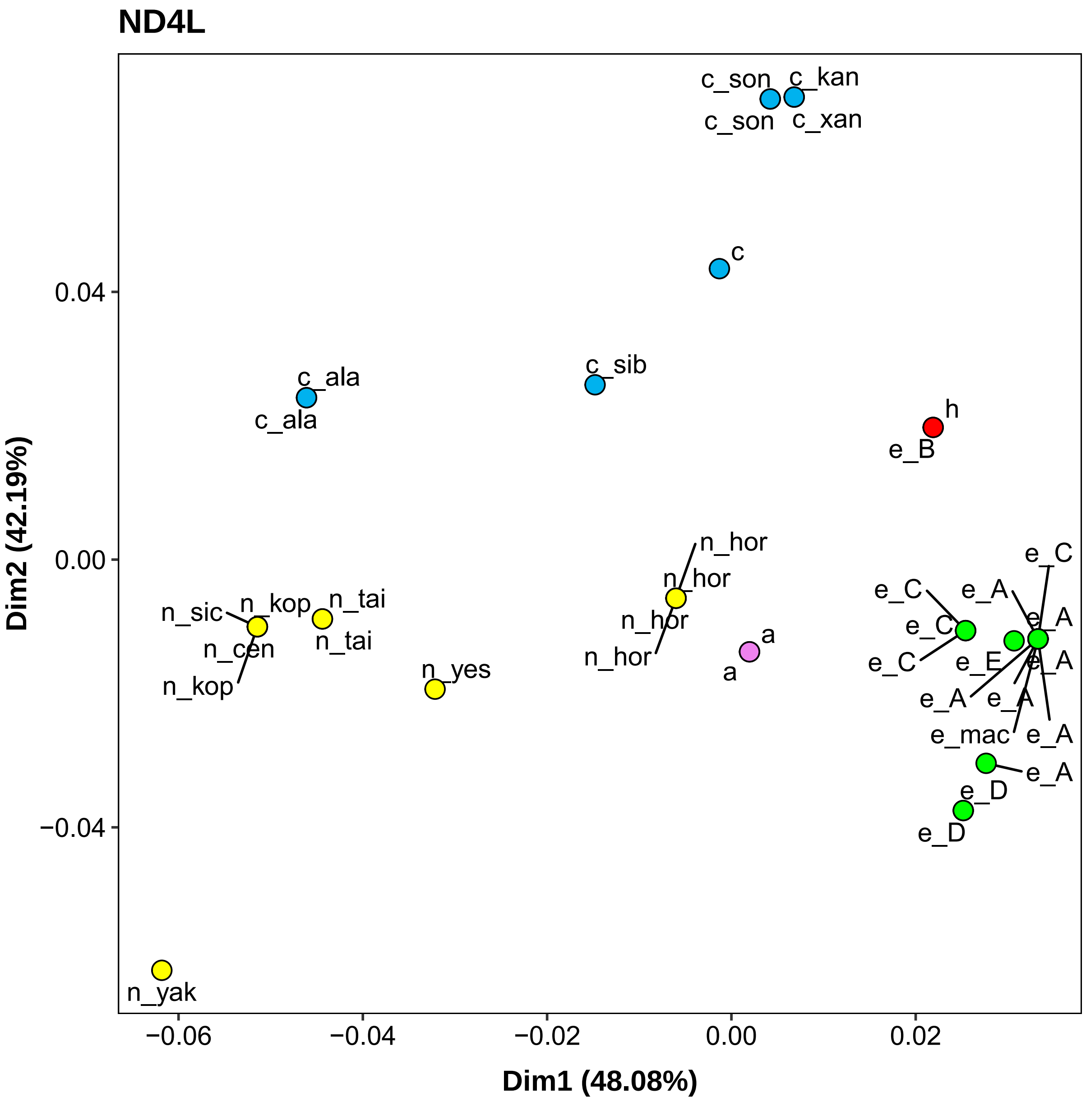

ND4L
c_son
c_kan
c_son
c_xan
c
c_sib
c_ala
c_ala
h
e_B
n_hor
n_hor
n_hor
n_hor
n_sic
n_kop
n_tai
n_cen
n_tai
n_kop
n_yes
a
a
e_C
e_C
e_A
e_A
e_C
e_C
e_E
e_A
e_A
e_A
e_mac
e_A
e_A
e_D
e_D
n_yak
0.04
0.00
−0.04
−0.06
−0.04
−0.02
0.00
0.02
Dim1 (48.08%)
Dim2 (42.19%)

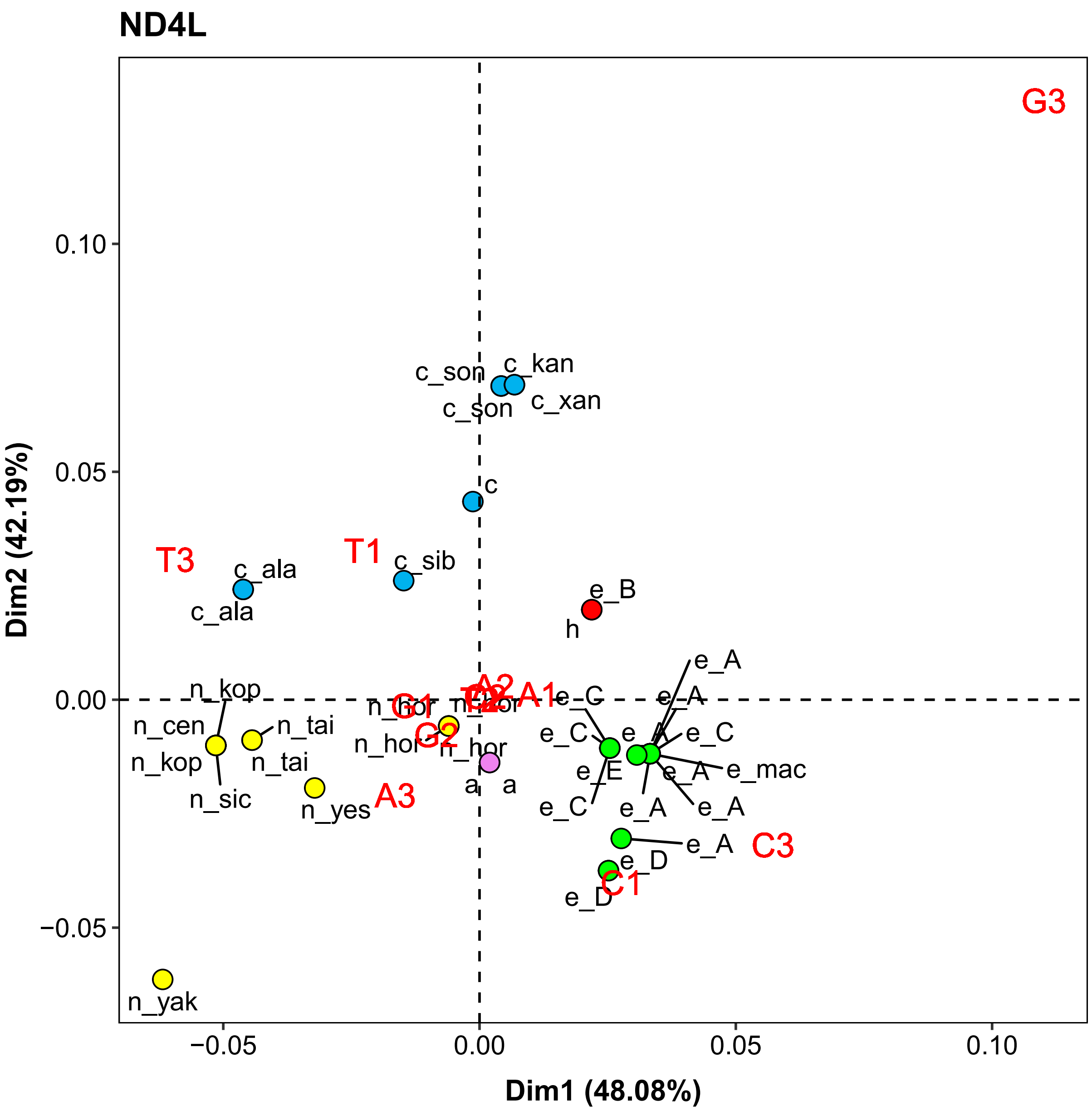
ND4L
Dim1 (48.08%)
Dim2 (42.19%)
0.10
0.05
0.00
−0.05
−0.05
0.00
0.05
0.10
G3
c_son
c_kan
c_son
c_xan
c
T3
T1
c_sib
c_ala
c_ala
e_B
h
e_A
n_kop
A2
A1
e_C
e_A
n_hor
G1
n_hor
n_cen
n_tai
G2
e_C
e_C
n_hor
n_hor
n_kop
n_tai
e_E
e_A
e_mac
a
a
n_sic
A3
e_C
e_A
e_A
n_yes
e_A
C3
e_D
C1
e_D
n_yak

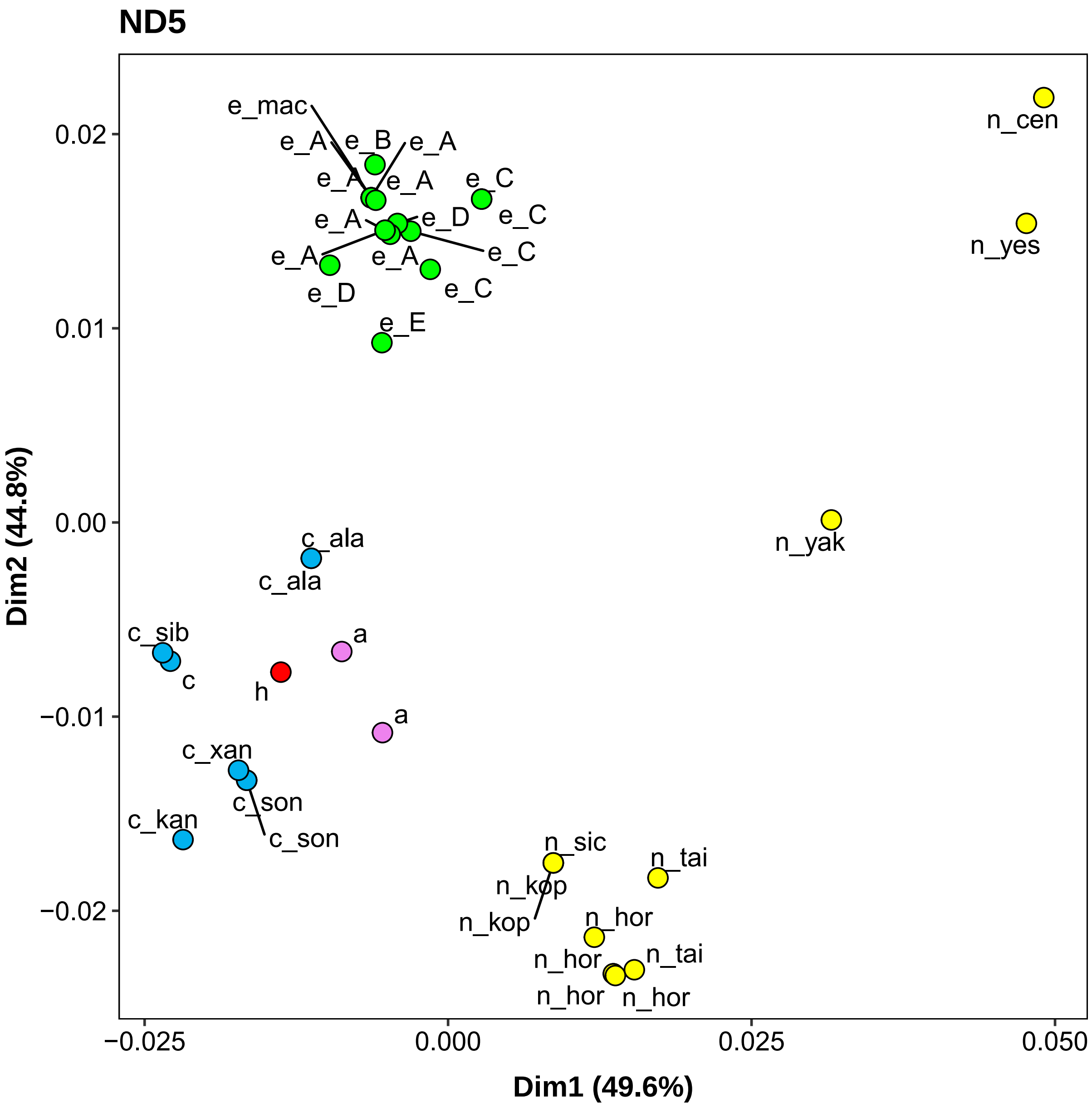

ND5
Dim1 (49.6%)
Dim2 (44.8%)
0.02
0.01
0.00
−0.01
−0.02
−0.025
0.000
0.025
0.050
e_mac
e_A
e_B
e_A
e_A
e_A
e_C
e_A
e_D
e_C
e_A
e_A
e_C
e_C
e_D
e_E
n_cen
n_yes
n_yak
c_ala
c_ala
c_sib
c
h
a
a
c_xan
c_son
c_kan
c_son
n_sic
n_tai
n_kop
n_kop
n_hor
n_hor
n_tai
n_hor
n_hor

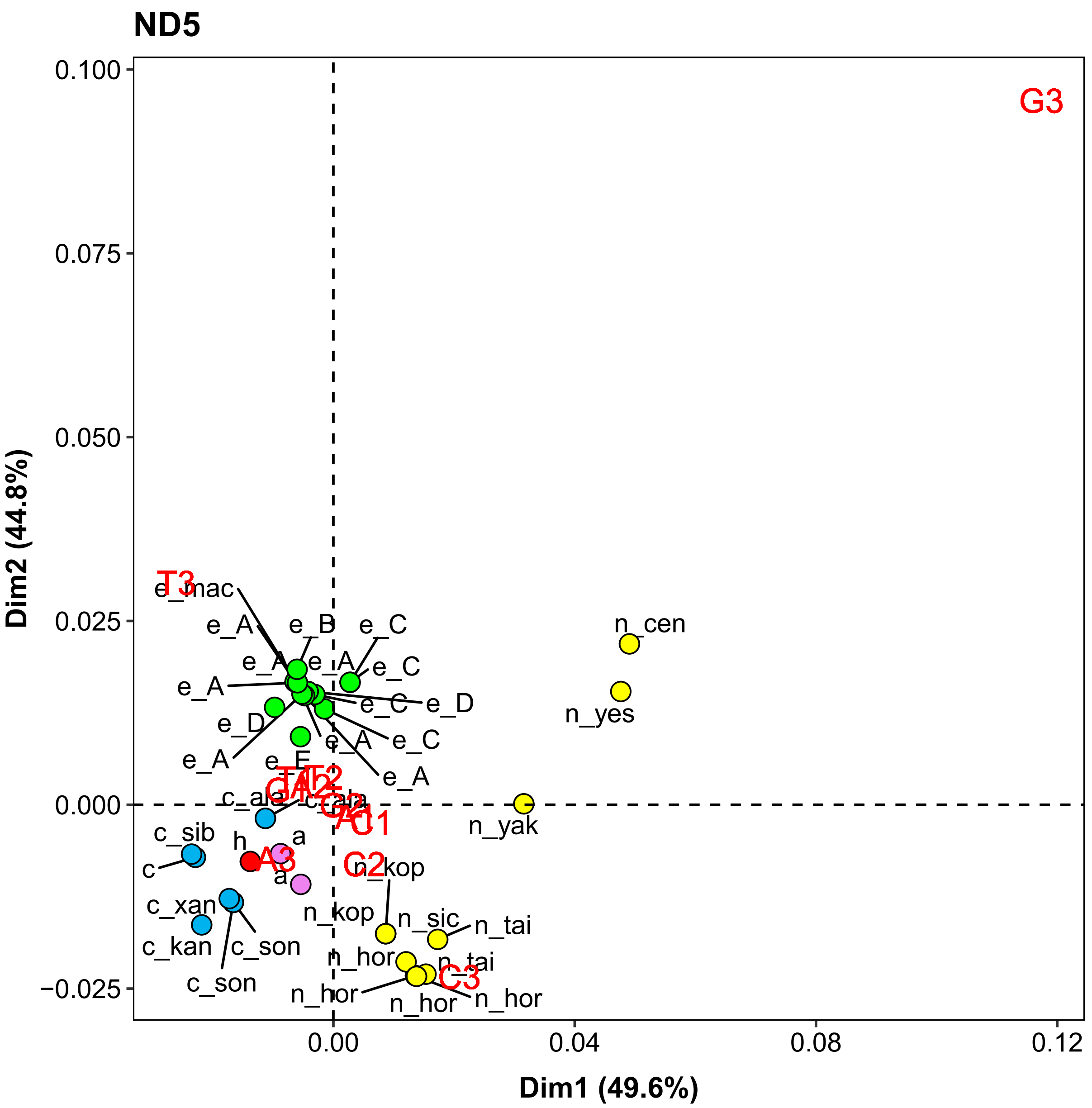
ND5
Dim1 (49.6%)
Dim2 (44.8%)
0.100
0.075
0.050
0.025
0.000
−0.025
0.00
0.04
0.08
0.12
G3
T3
e_mac
e_A
e_B
e_C
e_A
e_A
e_C
e_A
e_C
e_D
e_D
e_C
e_A
e_A
e_E
e_A
n_cen
n_yes
n_yak
c_ale
c_sib
c
h
a
a
A3
C2
n_kop
n_kop
n_sic
n_tai
n_hor
n_tai
C3
n_hor
n_hor
n_hor
c_xan
c_kan
c_son
c_son

ND6

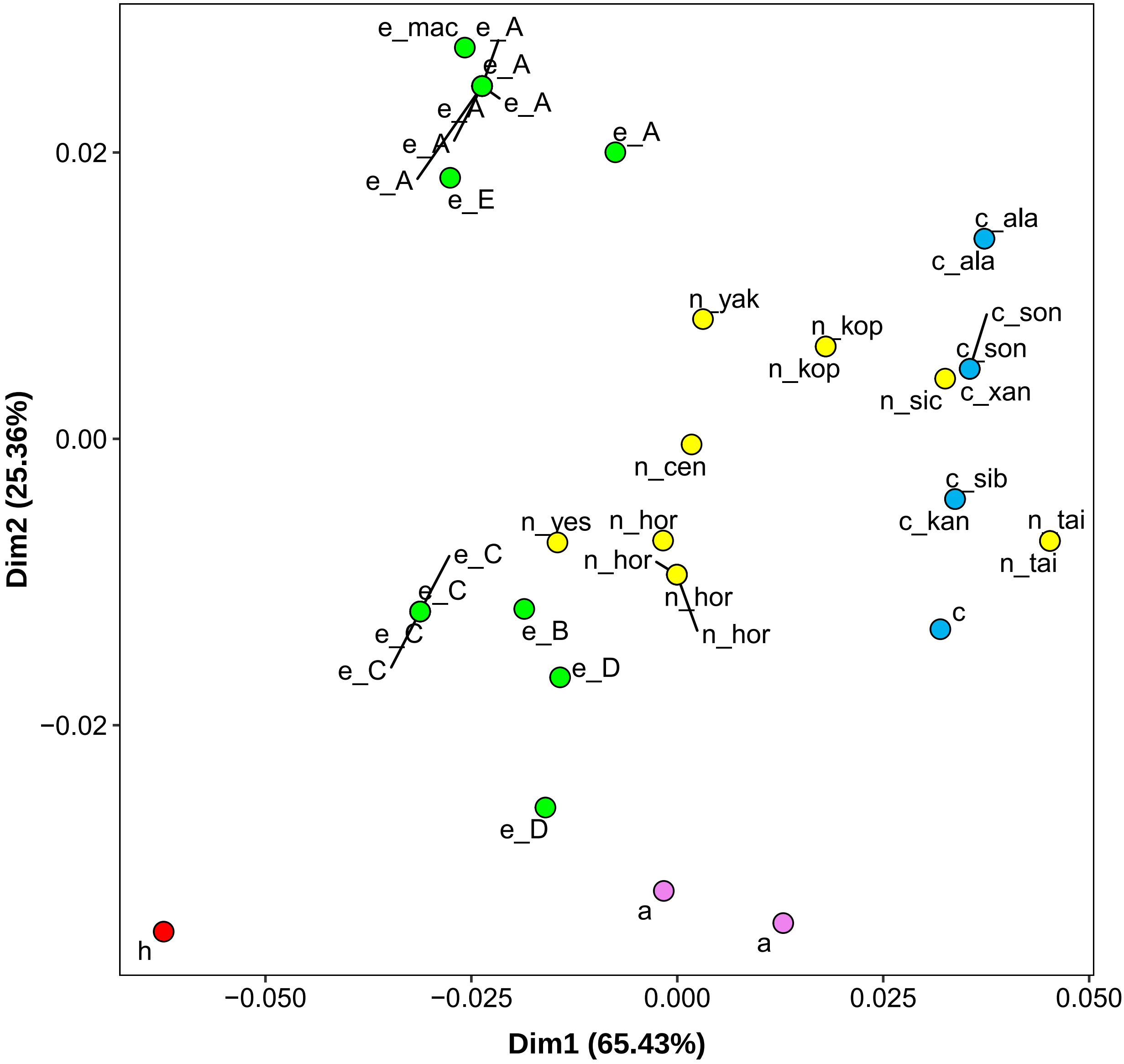

e_mac
e_A
e_A
e_A
e_A
e_A
e_A
e_A
e_E
c_ala
c_ala
n_yak
n_kop
n_kop
c_son
c_son
c_xan
n_sic
n_cen
c_sib
c_kan
n_tai
n_tai
n_yes
n_hor
n_hor
n_hor
n_hor
e_C
e_C
e_C
e_C
e_B
e_D
e_D
c
a
a
h
0.02
0.00
−0.02
−0.050
−0.025
0.000
0.025
0.050
Dim2 (25.36%)
Dim1 (65.43%)

ND6

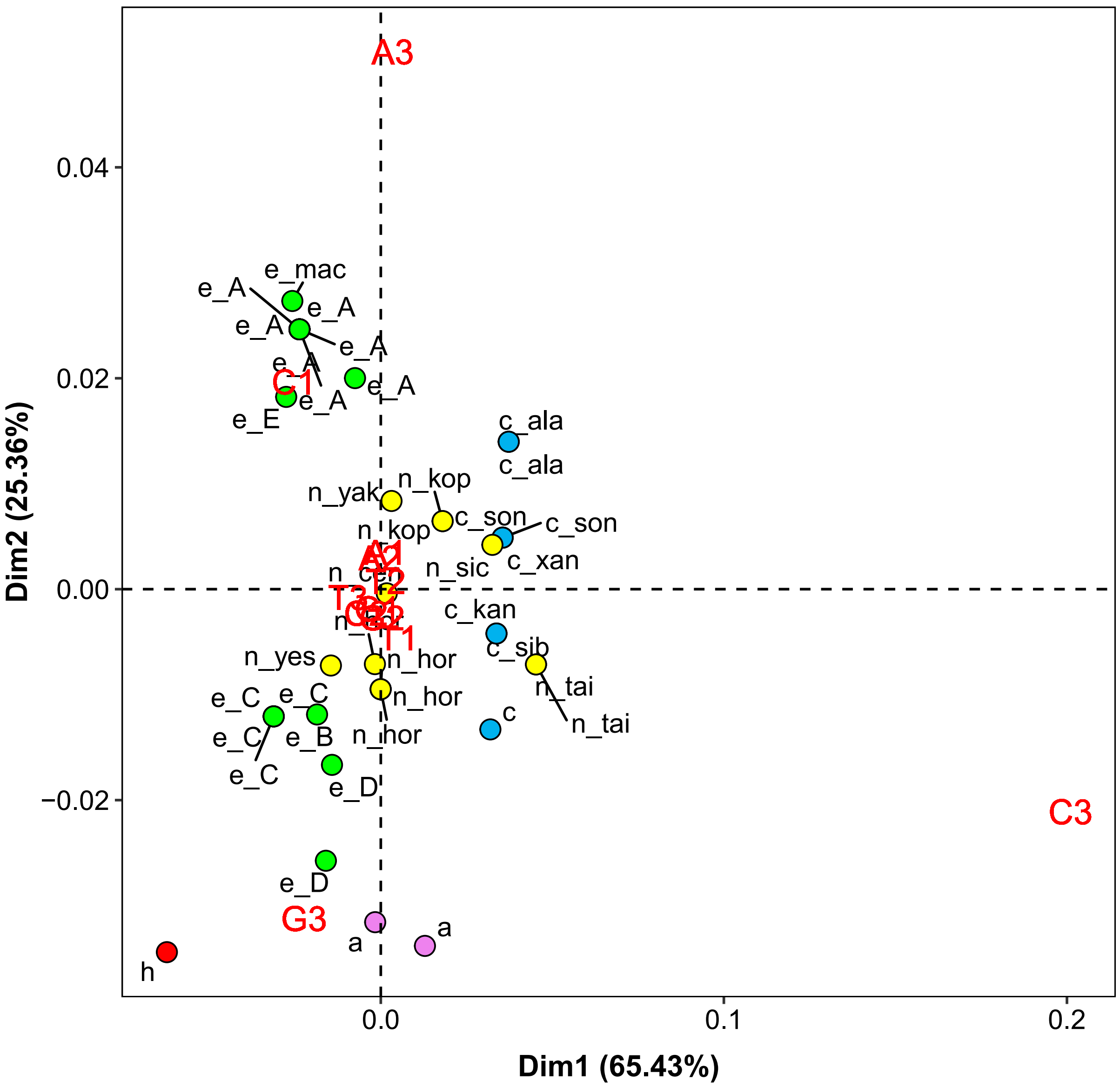
A3
C1
C3
G3
e_mac
e_A
e_E
e_C
e_B
e_D
c_ala
c_son
c_xan
c_kan
c_sib
c
n_yak
n_kop
n_sic
n_yes
n_hor
n_tai
a
h
0.04
0.02
0.00
−0.02
0.0
0.1
0.2
Dim1 (65.43%)
Dim2 (25.36%)

12S_RNA

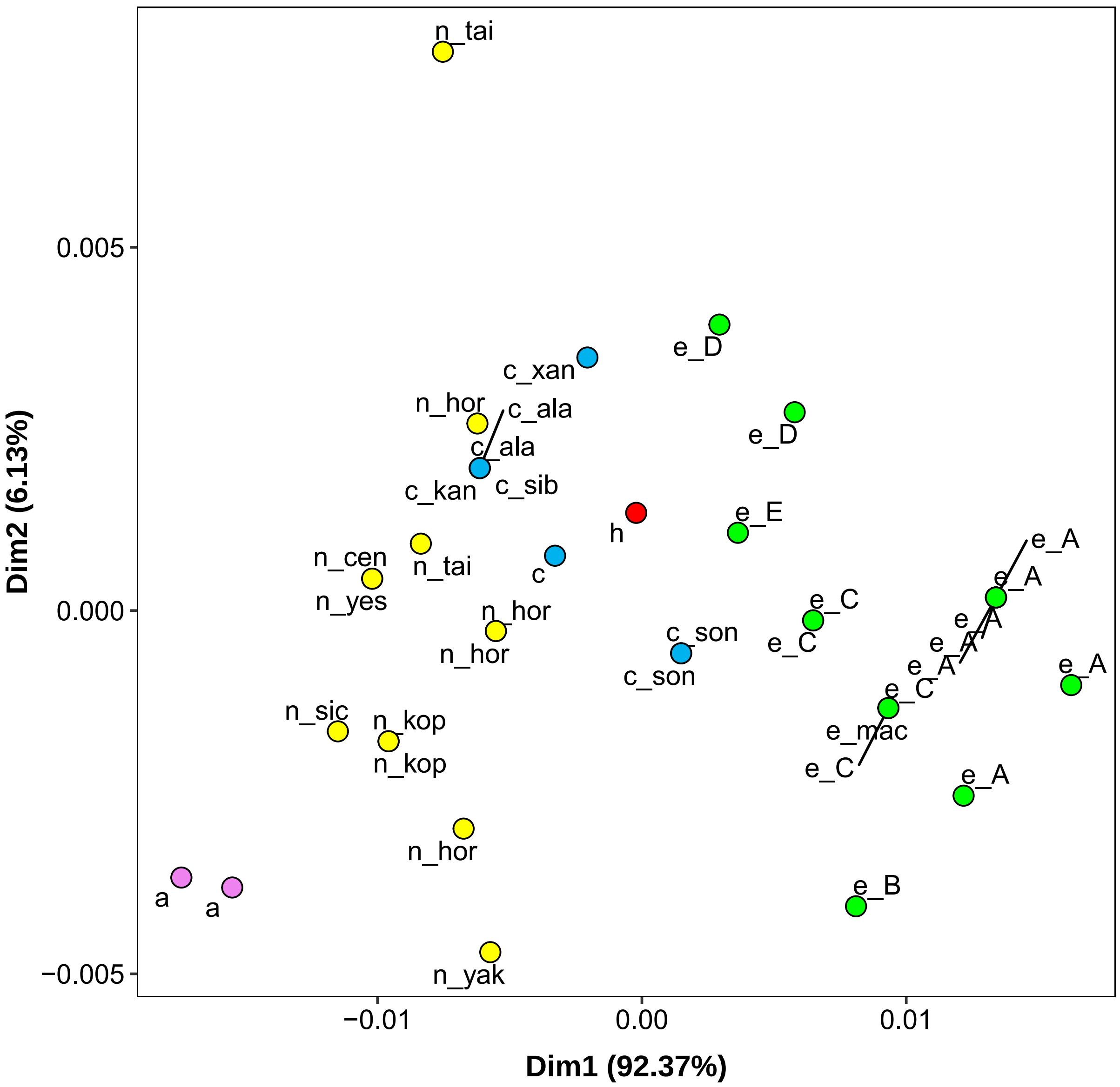

Dim2 (6.13%)
Dim1 (92.37%)
0.005
0.000
−0.005
−0.01
0.00
0.01
n_tai
e_D
c_xan
n_hor
c_ala
e_D
c_ala
c_kan
c_sib
h
e_E
n_cen
n_tai
c
e_A
e_A
n_yes
n_hor
e_C
c_son
e_A
n_hor
e_C
e_A
c_son
e_A
e_A
e_C
n_sic
n_kop
e_mac
n_kop
e_C
e_A
n_hor
a
a
e_B
n_yak

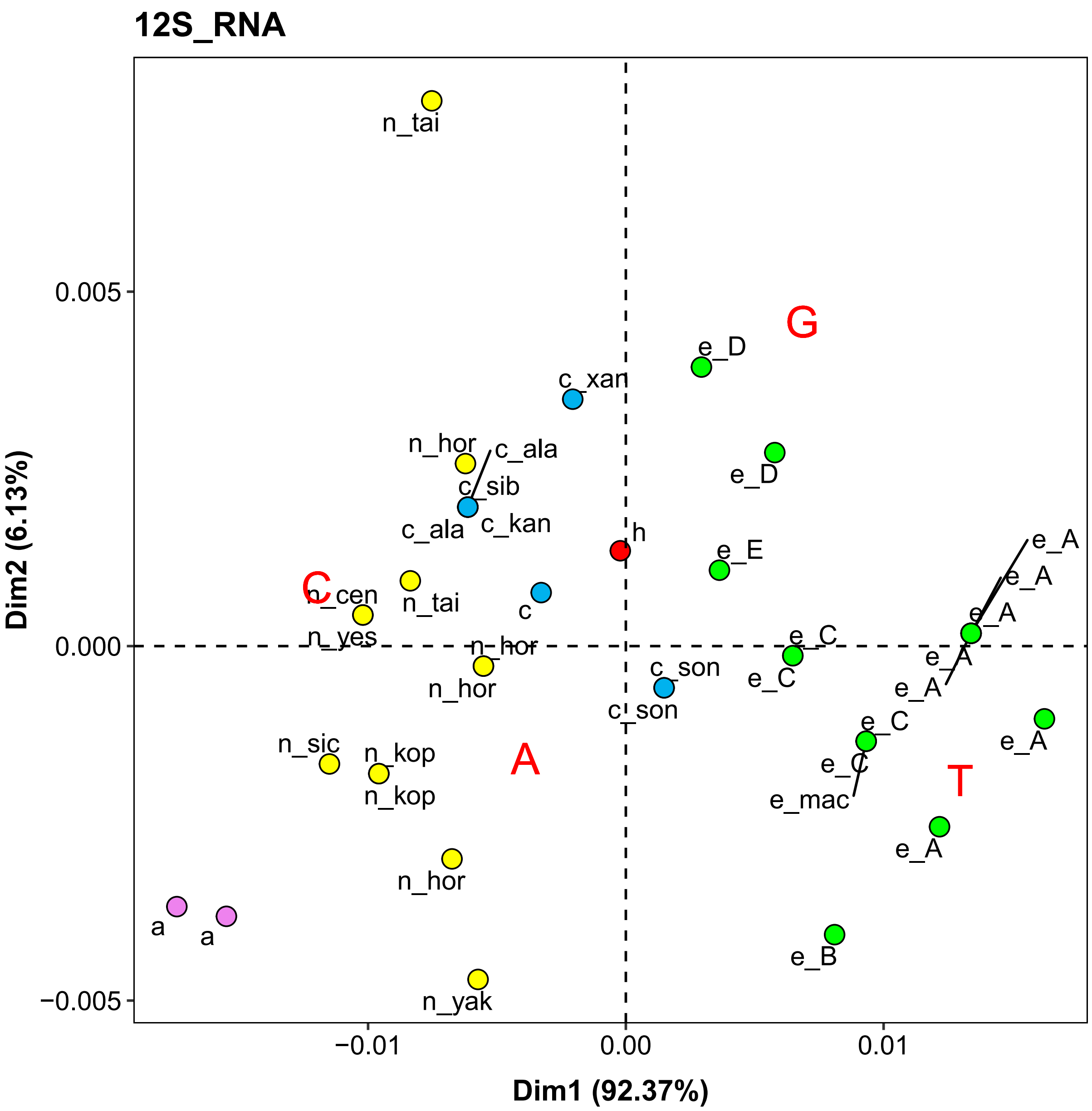
12S_RNA
Dim1 (92.37%)
Dim2 (6.13%)
0.005
0.000
−0.005
−0.01
0.00
0.01
G
C
A
T
n_tai
e_D
c_xan
n_hor
c_ala
e_D
c_sib
c_ala
c_kan
h
e_E
e_A
e_A
e_A
n_cen
n_tai
c
n_yes
n_hor
e_C
e_A
e_C
c_son
n_hor
e_A
c_son
e_C
e_A
n_sic
n_kop
e_C
n_kop
e_mac
e_A
n_hor
a
a
e_B
n_yak

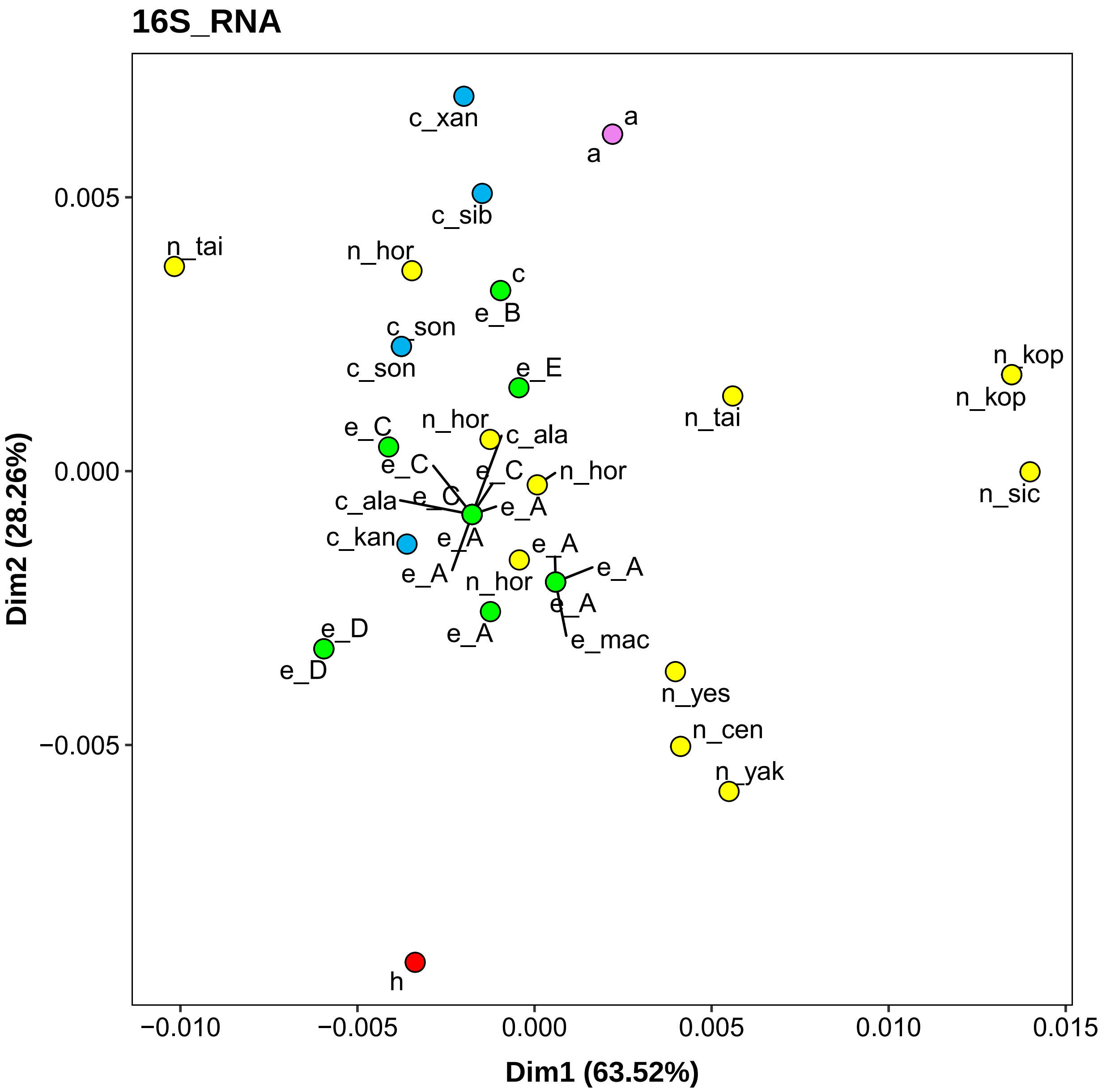
16S_RNA
Dim2 (28.26%)
Dim1 (63.52%)
0.005
0.000
−0.005
−0.010
−0.005
0.000
0.005
0.010
0.015
c_xan
a
a
c_sib
n_tai
n_hor
c
e_B
c_son
c_son
e_E
n_kop
n_kop
n_tai
e_C
n_hor
c_ala
e_C
e_C
n_hor
c_ala
e_C
e_A
n_sic
c_kan
e_A
e_A
e_A
e_A
n_hor
e_A
e_D
e_A
e_mac
e_D
n_yes
n_cen
n_yak
h

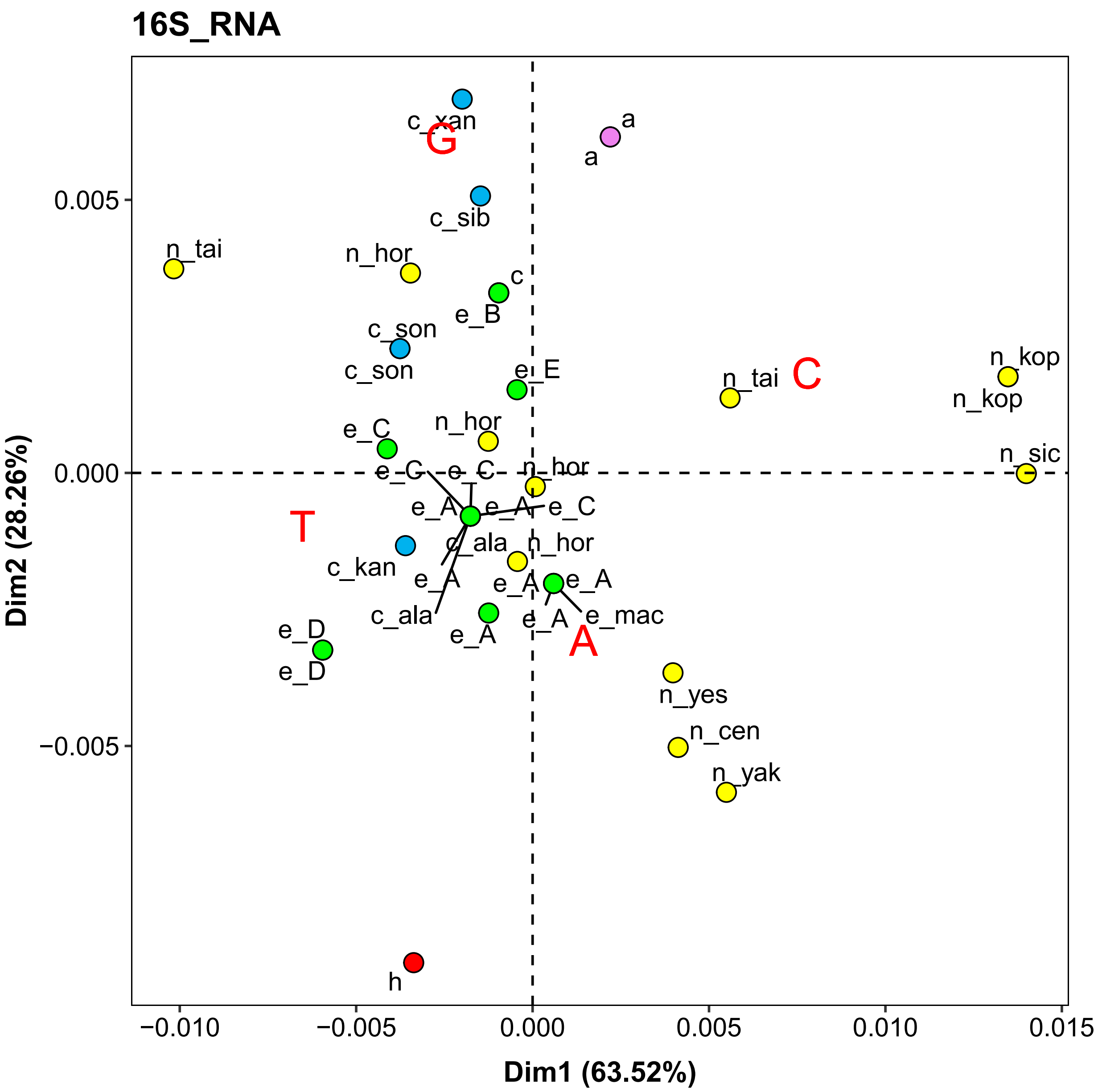
16S_RNA
Dim1 (63.52%)
Dim2 (28.26%)
0.005
0.000
−0.005
−0.010
0.010
0.015
G
C
T
A
c_xan
a
a
c_sib
n_tai
n_hor
c
e_B
c_son
c_son
e_E
n_tai
n_kop
n_kop
e_C
n_hor
n_sic
e_C
e_C
n_hor
e_A
e_A
e_C
c_ala
n_hor
c_kan
e_A
e_A
e_A
c_ala
e_mac
e_A
e_A
e_D
e_D
n_yes
n_cen
n_yak
h

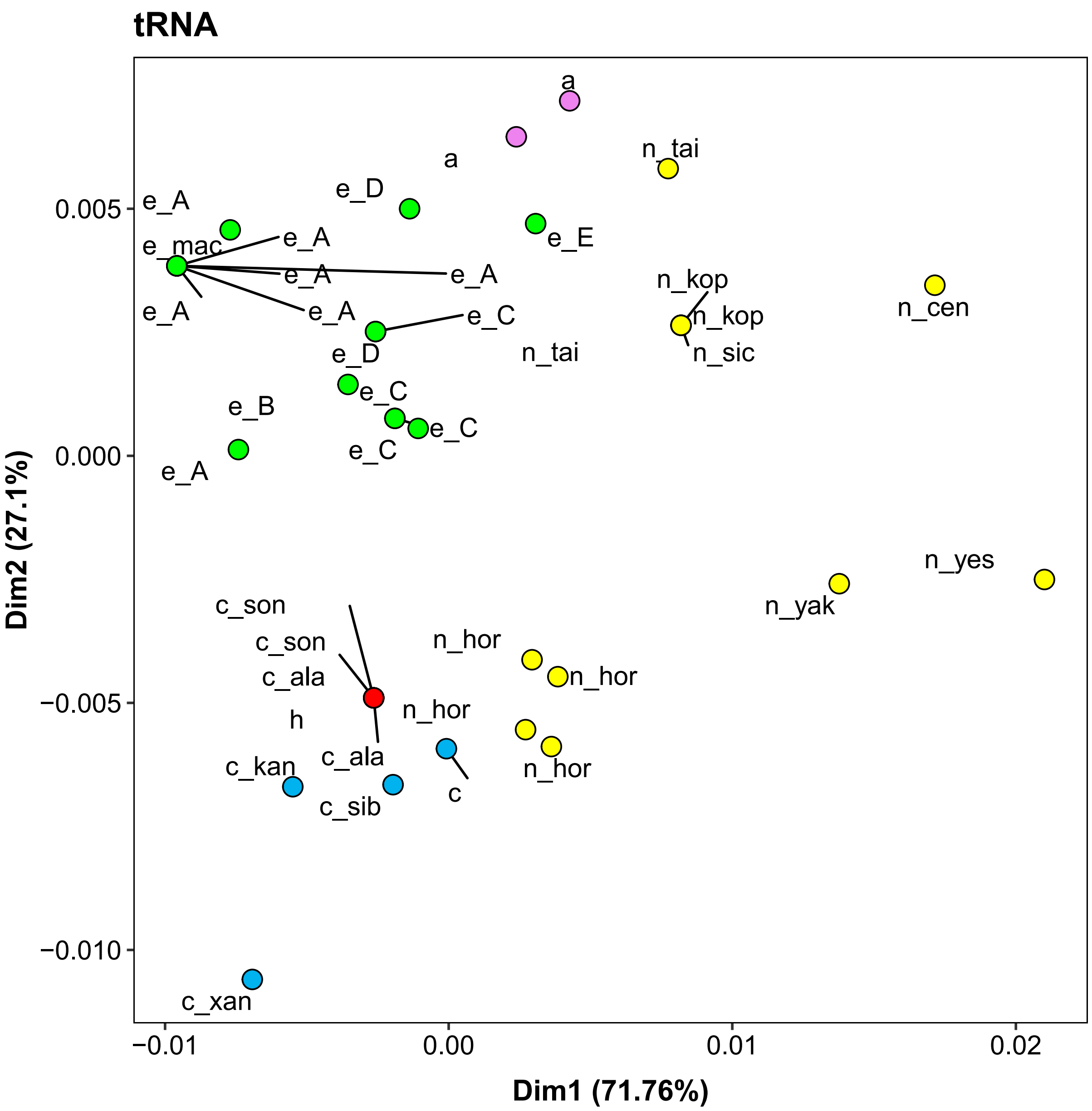

tRNA
a
a
n_tai
e_D
e_A
e_A
e_mac
e_A
e_A
e_A
e_A
e_E
n_kop
n_kop
n_cen
e_C
n_tai
n_sic
e_D
e_C
e_B
e_C
e_C
e_A
n_yes
n_yak
c_son
c_son
n_hor
c_ala
n_hor
h
n_hor
c_ala
c_kan
c
n_hor
c_sib
c_xan
0.005
0.000
−0.005
−0.010
−0.01
0.00
0.01
0.02
Dim1 (71.76%)
Dim2 (27.1%)

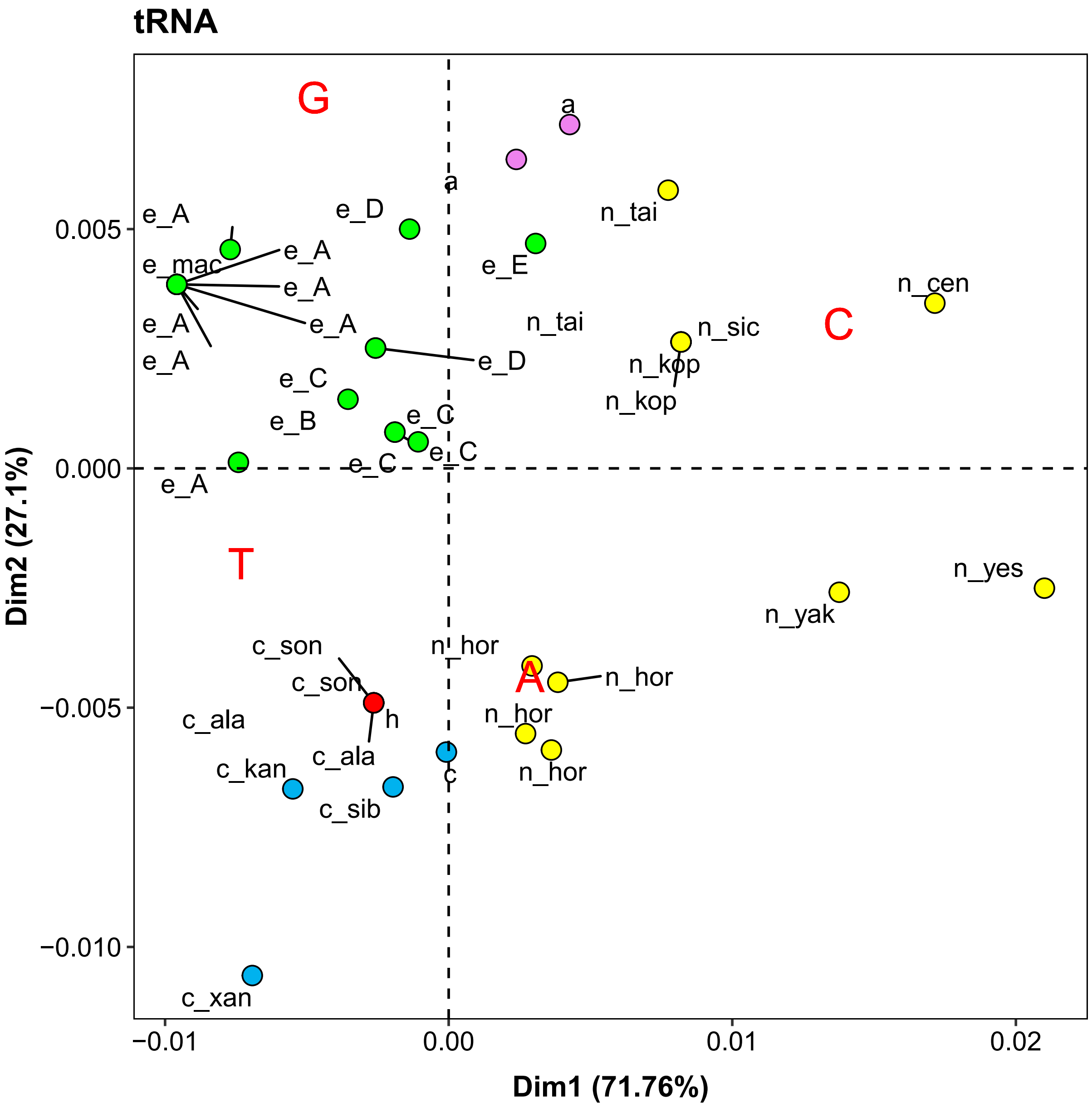
tRNA
G
C
T
A
a
a
n_tai
n_tai
e_D
e_D
e_E
e_A
e_A
e_A
e_A
e_A
e_A
e_A
e_mac
e_C
e_C
e_C
e_C
e_B
n_cen
n_sic
n_kop
n_kop
n_yes
n_yak
n_hor
n_hor
n_hor
n_hor
c_son
c_son
h
c_ala
c_ala
c_kan
c_sib
c
c_xan
0.005
0.000
−0.005
−0.010
−0.01
0.00
0.01
0.02
Dim1 (71.76%)
Dim2 (27.1%)

CR

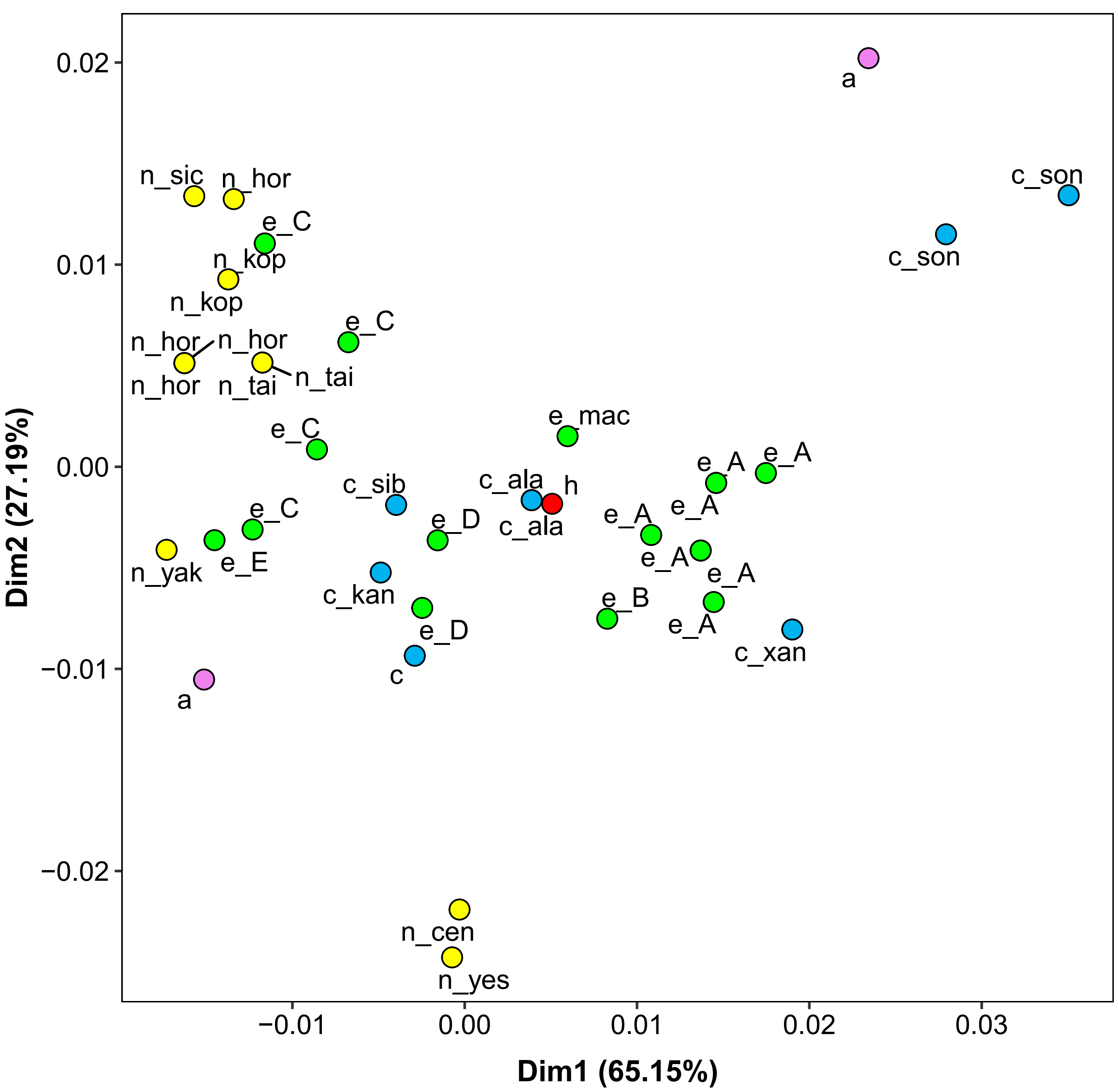

Dim1 (65.15%)
Dim2 (27.19%)
−0.01
0.00
0.01
0.02
0.03
0.02
0.01
0.00
−0.01
−0.02
a
c_son
c_son
n_sic
n_hor
e_C
n_kop
n_kop
e_C
n_hor
n_hor
n_hor
n_tai
n_tai
e_mac
e_C
e_A
e_A
c_sib
c_ala
h
c_ala
e_A
e_A
e_C
e_D
e_A
n_yak
e_E
e_A
c_kan
e_B
e_D
e_A
c_xan
c
a
n_cen
n_yes

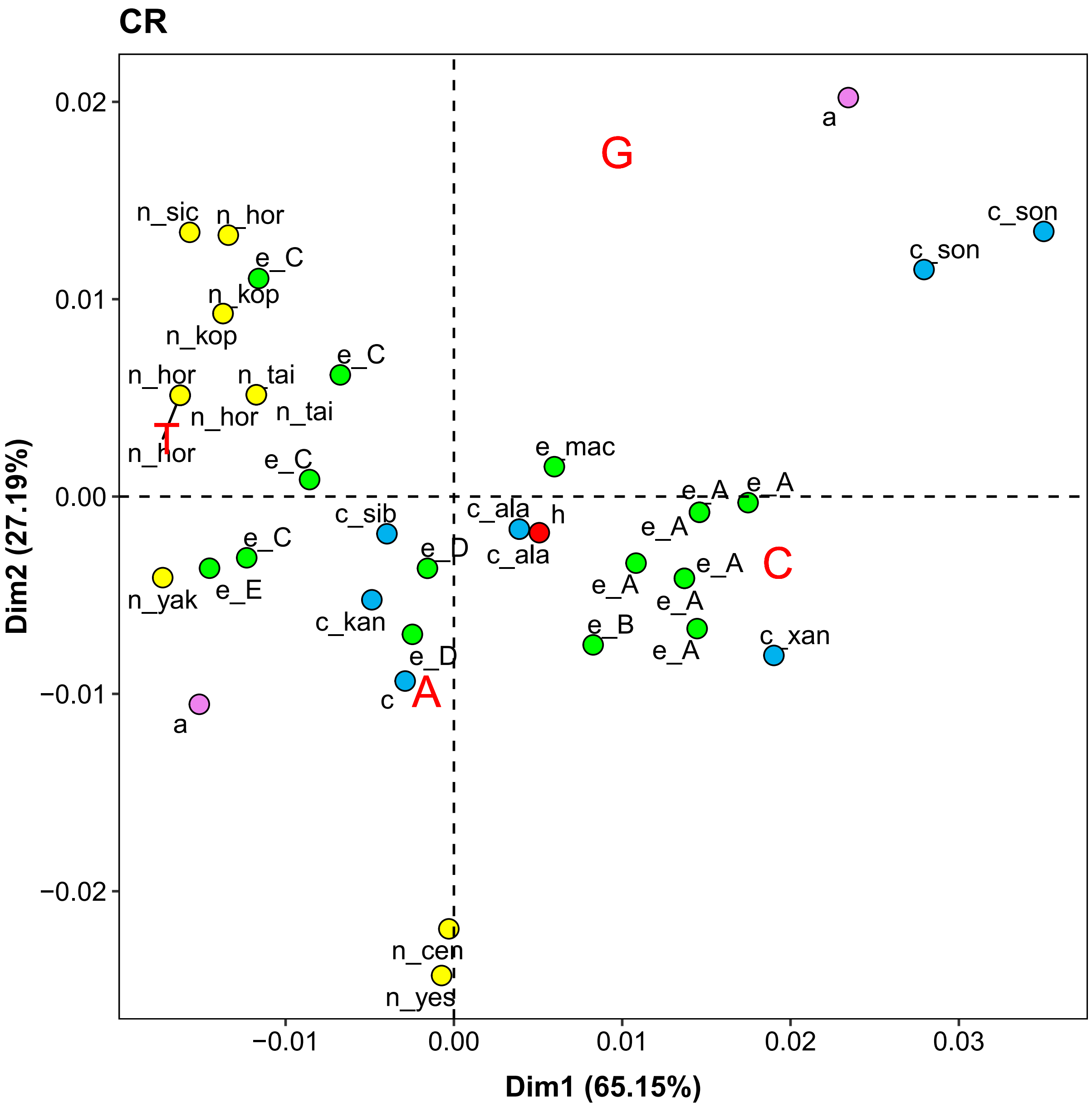
CR
Dim1 (65.15%)
Dim2 (27.19%)
0.02
0.01
0.00
−0.01
−0.02
0.00
0.01
0.02
0.03
G
T
C
A
n_sic
n_hor
e_C
n_kop
n_kop
e_C
n_hor
n_tai
n_hor
n_tai
n_hor
e_C
e_mac
e_A
e_A
c_sib
c_ala
h
e_A
e_C
e_D
c_ala
e_A
e_A
e_E
n_yak
e_A
e_A
c_kan
e_B
c_xan
e_D
e_A
c
a
a
c_son
c_son
n_cen
n_yes

Figure S2. Comparison of p-distances for all pairs of *Cervus* species with the distances obtained from the MrBayes phylogenetic tree for 13 protein-coding genes (PCG), RNA genes and control region (CR). The species pairs were arranged according to the tree distance. Ca - *C. albirostris*; Cc - *C. canadensis*; Ce - *C. elaphus*; Ch - *C. hanglu*; Cn - *C. nippon.*

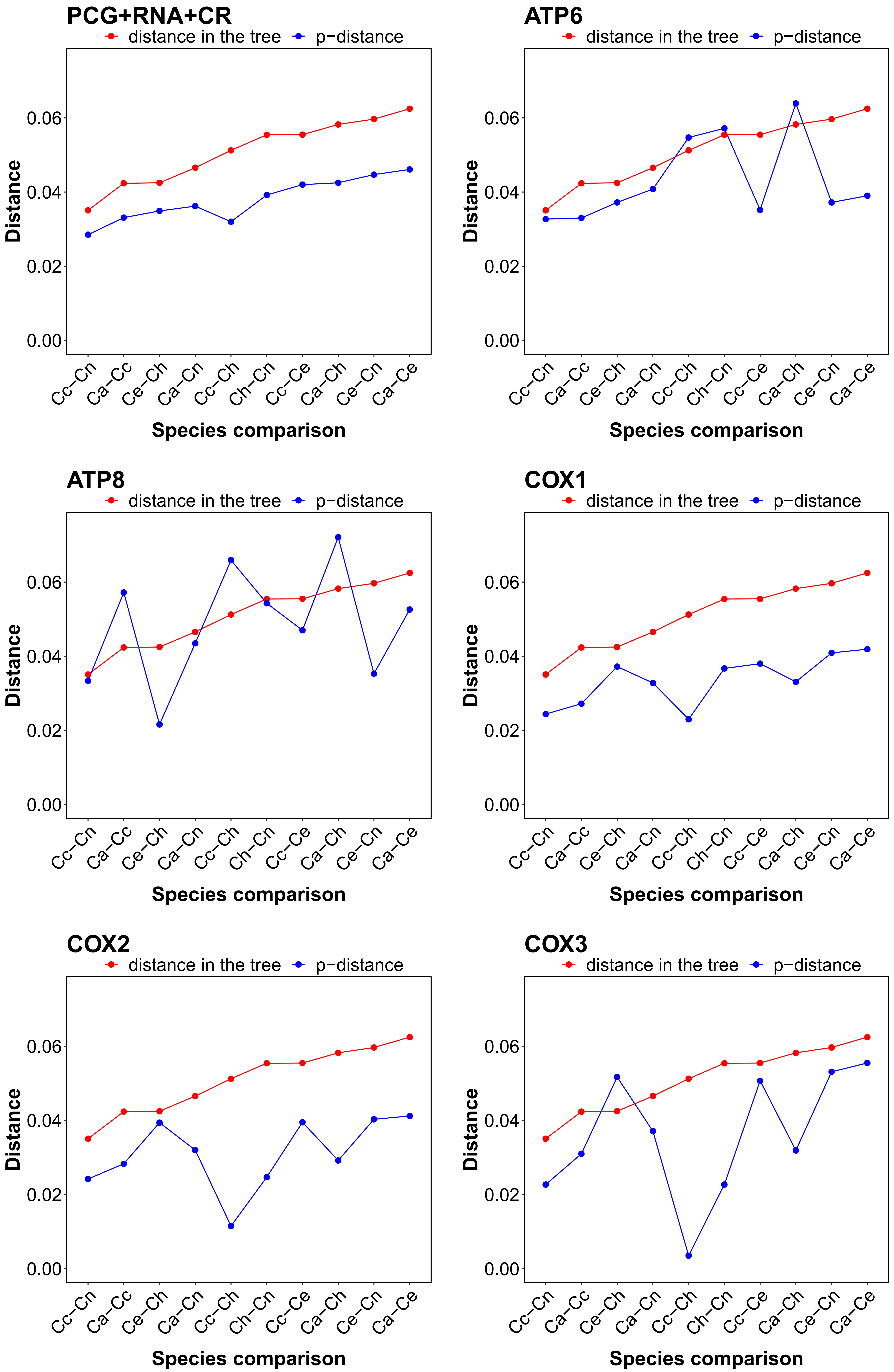
PCG+RNA+CR
ATP6
ATP8
COX1
COX2
COX3
distance in the tree
p−distance
Distance
0.00
0.02
0.04
0.06
Cc−Cn
Ca−Cc
Ce−Ch
Ca−Cn
Cc−Ch
Ch−Cn
Cc−Ce
Ca−Ch
Ce−Cn
Ca−Ce
Species comparison

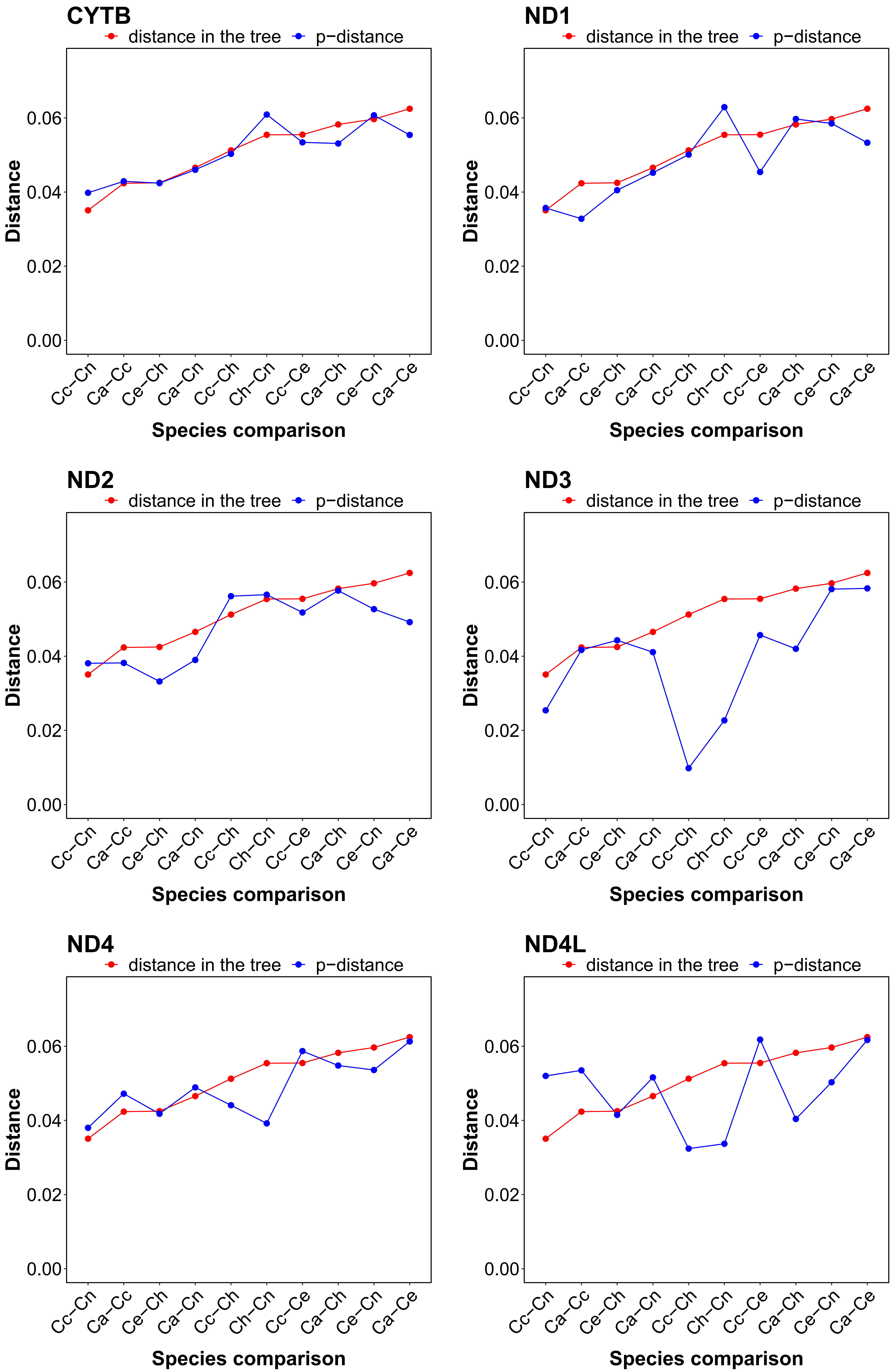
CYTB
ND1
ND2
ND3
ND4
ND4L
distance in the tree
p−distance
Distance
Species comparison
0.00
0.02
0.04
0.06
Cc−Cn
Ca−Cc
Ce−Ch
Ca−Cn
Cc−Ch
Ch−Cn
Cc−Ce
Ca−Ch
Ce−Cn
Ca−Ce

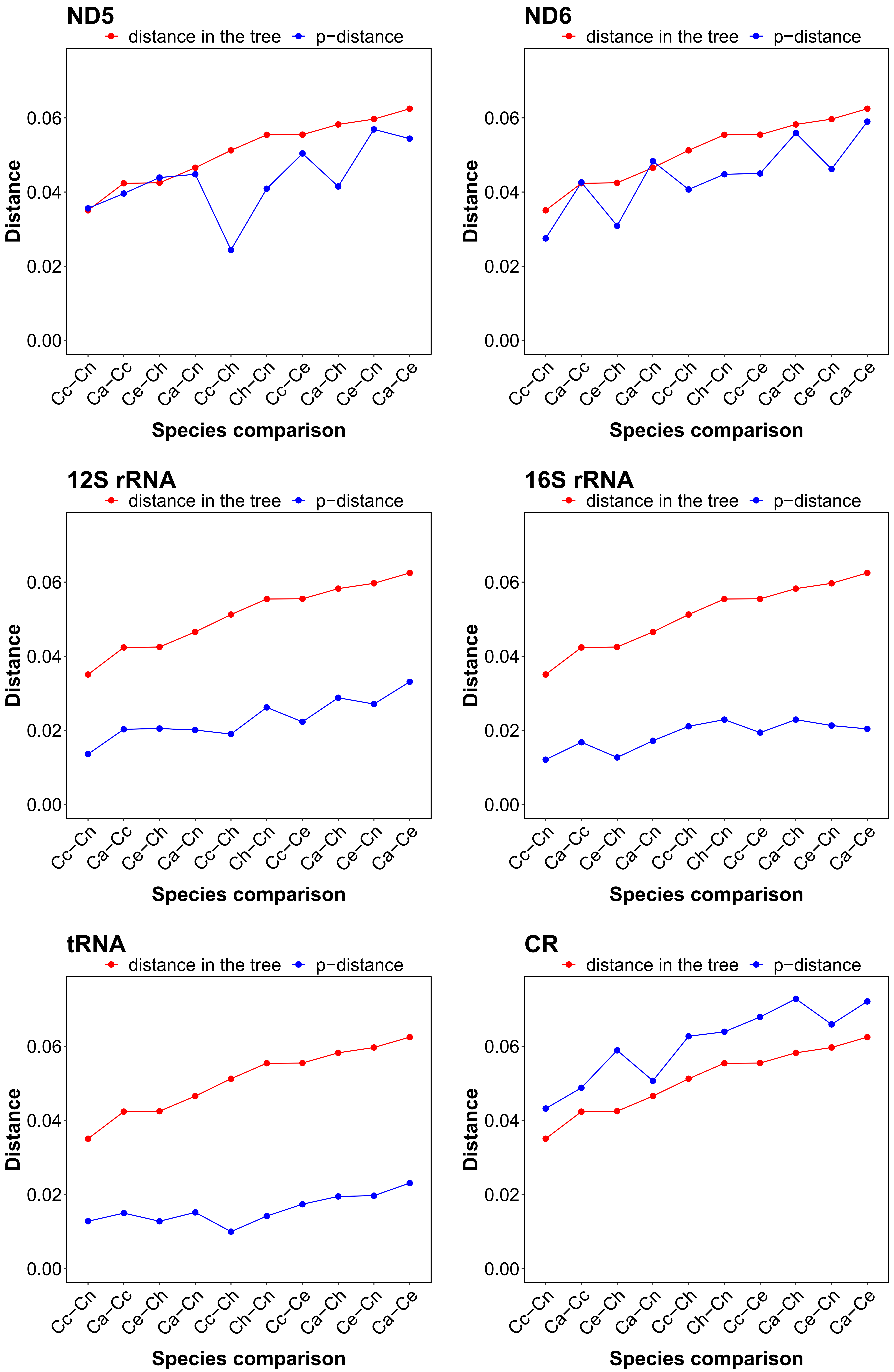
ND5
distance in the tree
p−distance
Distance
0.00
0.02
0.04
0.06
Cc−Cn
Ca−Cc
Ce−Ch
Ca−Cn
Cc−Ch
Ch−Cn
Cc−Ce
Ca−Ch
Ce−Cn
Ca−Ce
Species comparison
ND6
distance in the tree
p−distance
Distance
0.00
0.02
0.04
0.06
Cc−Cn
Ca−Cc
Ce−Ch
Ca−Cn
Cc−Ch
Ch−Cn
Cc−Ce
Ca−Ch
Ce−Cn
Ca−Ce
Species comparison
12S rRNA
distance in the tree
p−distance
Distance
0.00
0.02
0.04
0.06
Cc−Cn
Ca−Cc
Ce−Ch
Ca−Cn
Cc−Ch
Ch−Cn
Cc−Ce
Ca−Ch
Ce−Cn
Ca−Ce
Species comparison
16S rRNA
distance in the tree
p−distance
Distance
0.00
0.02
0.04
0.06
Cc−Cn
Ca−Cc
Ce−Ch
Ca−Cn
Cc−Ch
Ch−Cn
Cc−Ce
Ca−Ch
Ce−Cn
Ca−Ce
Species comparison
tRNA
distance in the tree
p−distance
Distance
0.00
0.02
0.04
0.06
Cc−Cn
Ca−Cc
Ce−Ch
Ca−Cn
Cc−Ch
Ch−Cn
Cc−Ce
Ca−Ch
Ce−Cn
Ca−Ce
Species comparison
CR
distance in the tree
p−distance
Distance
0.00
0.02
0.04
0.06
Cc−Cn
Ca−Cc
Ce−Ch
Ca−Cn
Cc−Ch
Ch−Cn
Cc−Ce
Ca−Ch
Ce−Cn
Ca−Ce
Species comparison

Figure S3. Relationships between the distances in the MrBayes phylogenetic tree and p-distances for all pairs of *Cervus* species and 13 protein-coding genes (PCG), RNA genes and control region (CR). A regression line (red line) with 95% confidence interval (grey area) was also presented. Ca - *C. albirostris*; Cc - *C. canadensis*; Ce - *C. elaphus*; Ch - *C. hanglu*; Cn - *C. nippon.*

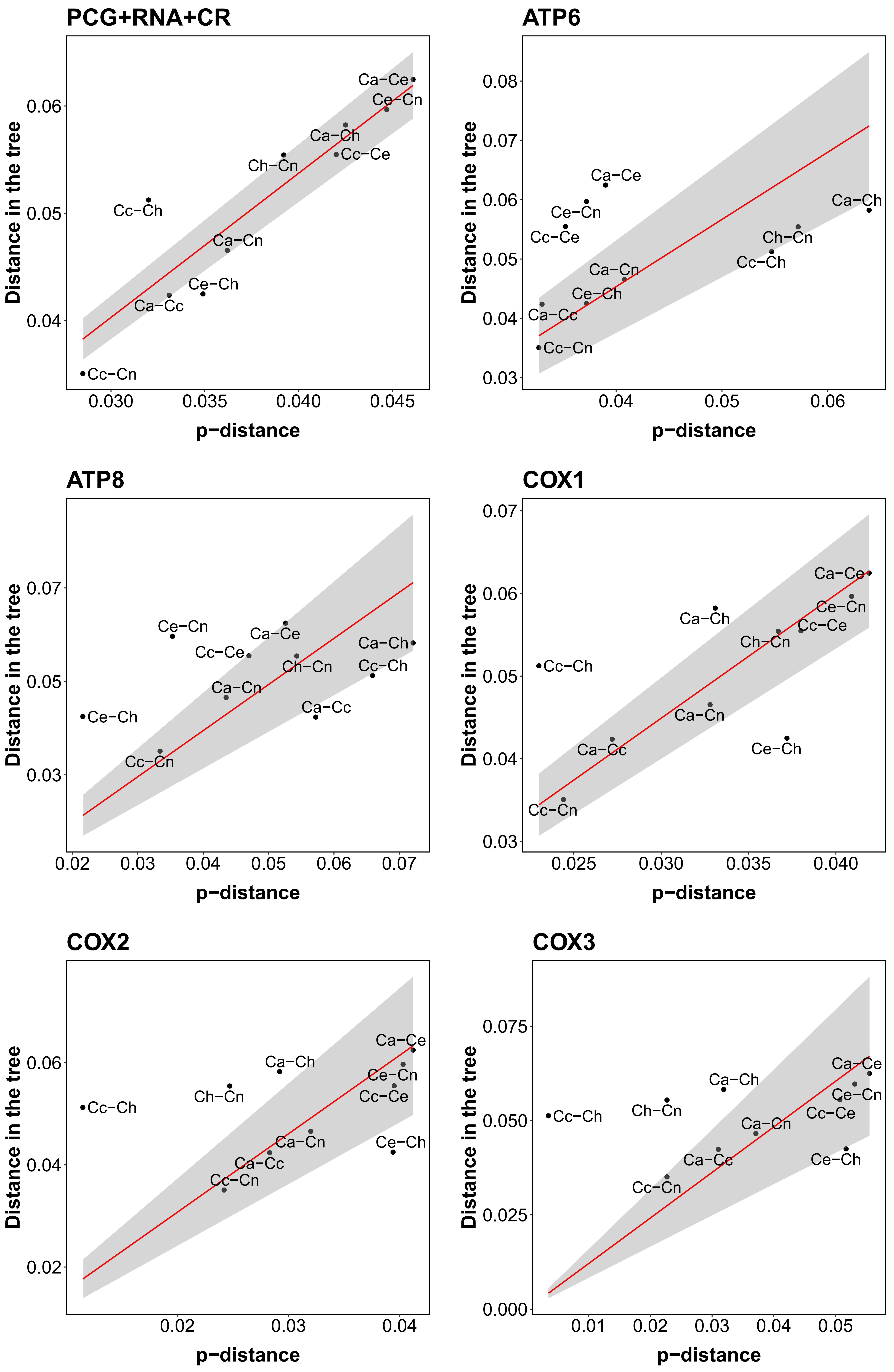

PCG+RNA+CR
ATP6
ATP8
COX1
COX2
COX3
Distance in the tree
p−distance
Ca−Ce
Ce−Cn
Ca−Ch
Ch−Cn
Cc−Ce
Cc−Ch
Ca−Cn
Ce−Ch
Ca−Cc
Cc−Cn

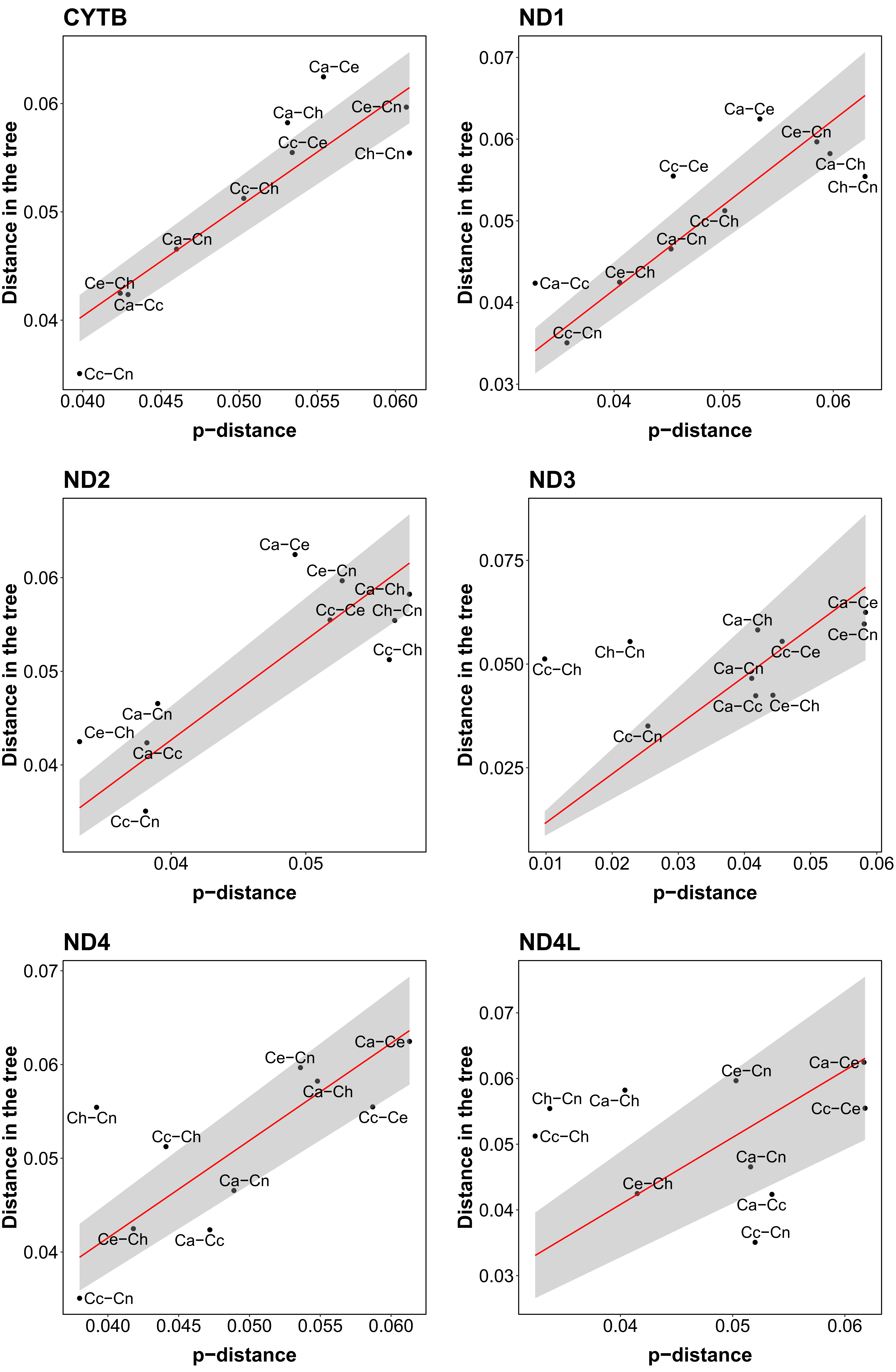
CYTB
ND1
ND2
ND3
ND4
ND4L
Distance in the tree
p−distance
Ca−Ce
Ca−Ch
Ca−Cn
Ca−Cc
Cc−Ce
Cc−Ch
Cc−Cn
Ce−Cn
Ce−Ch
Ch−Cn
0.030
0.035
0.040
0.045
0.050
0.055
0.060
0.065
0.07
0.075
0.025
0.01
0.02
0.03
0.04
0.05
0.06

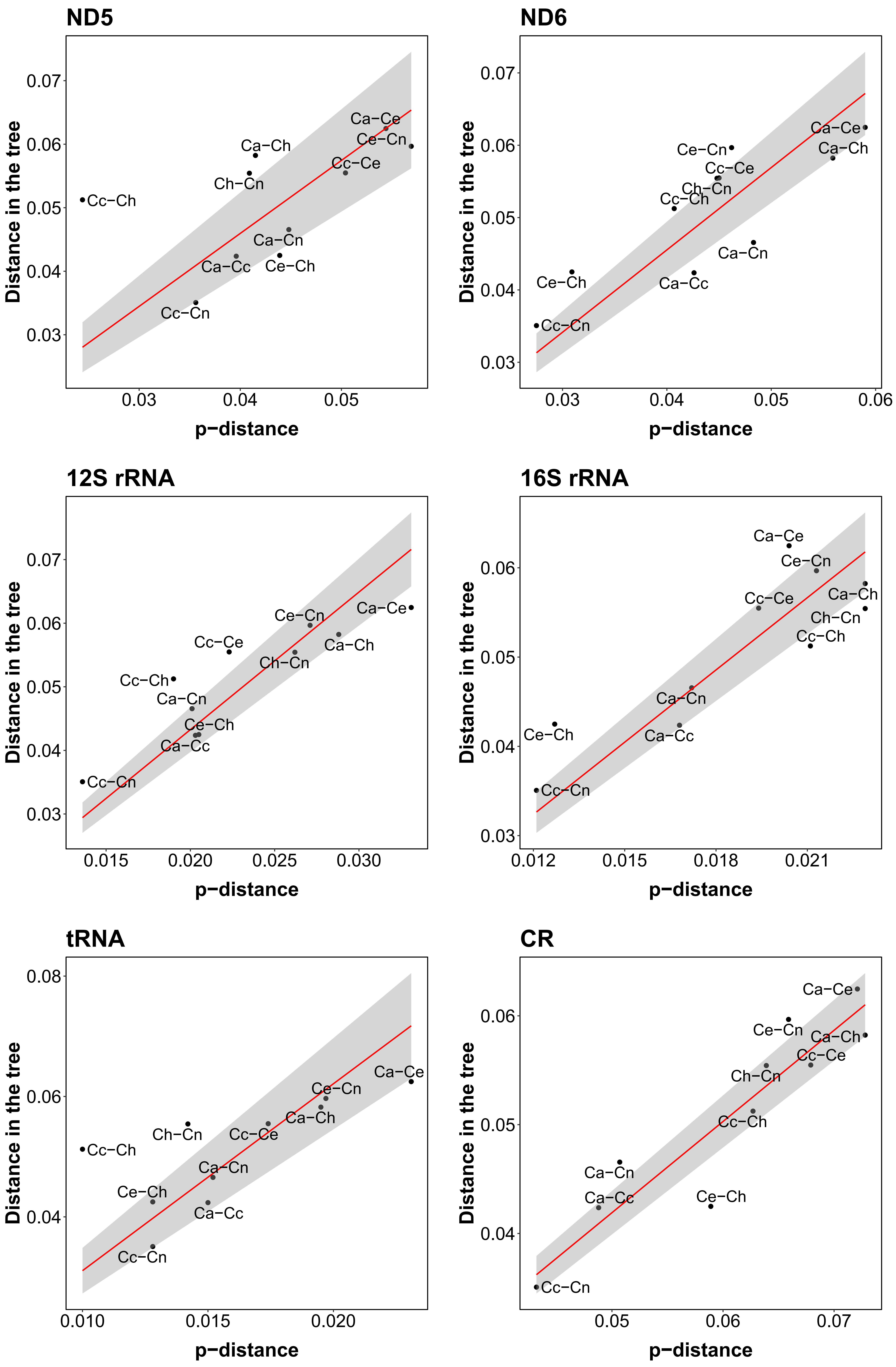
ND5
ND6
12S rRNA
16S rRNA
tRNA
CR
Distance in the tree
p−distance
Ca−Ce
Ce−Cn
Ca−Ch
Cc−Ce
Ch−Cn
Cc−Ch
Ca−Cn
Ca−Cc
Ce−Ch
Cc−Cn

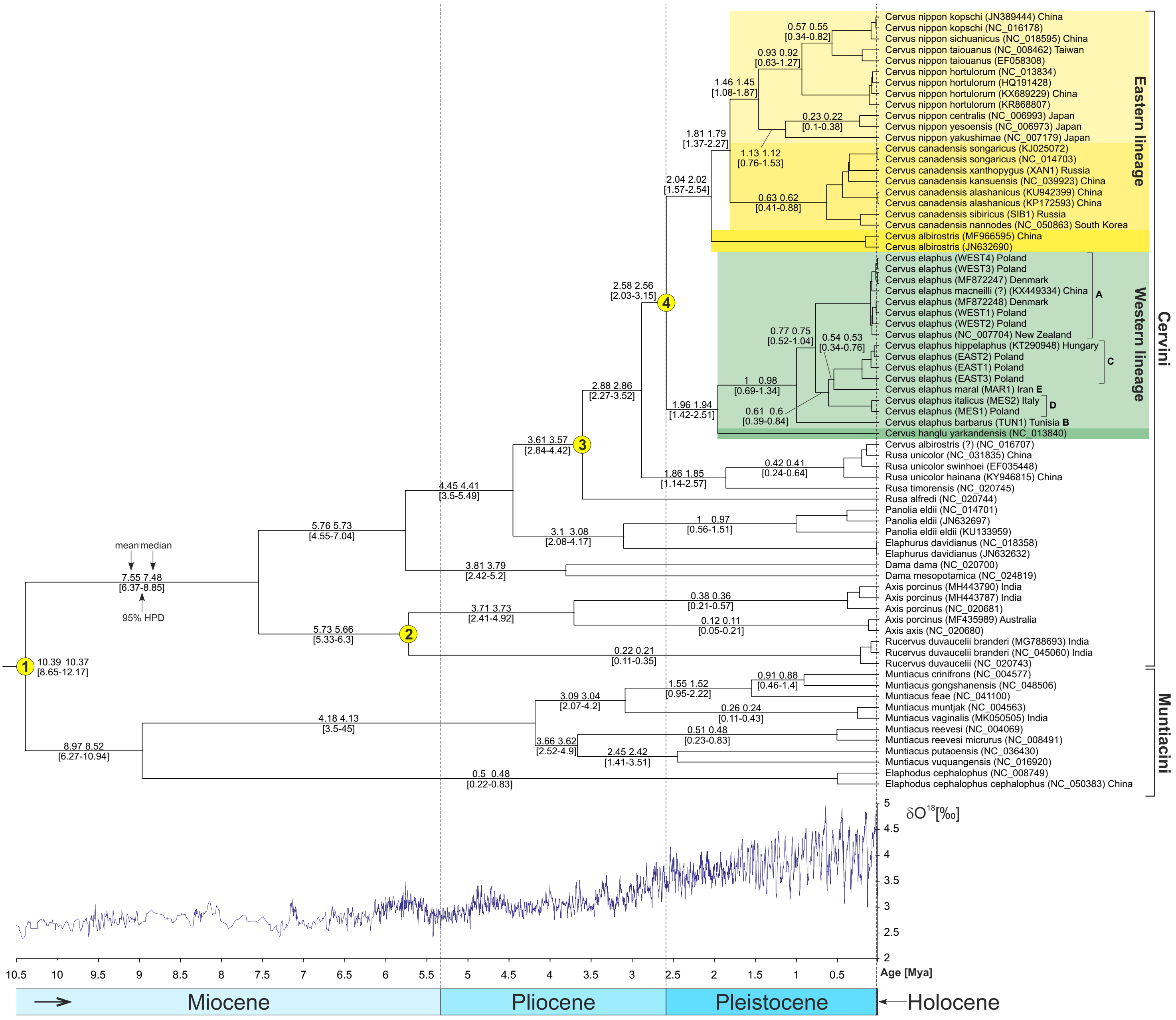

Figure S4. The chronogram for Muntiacini and Cervini with the oxygen isotopic curve (Zachos *et al.,* 2001). Mean and median as well as 95% Highest Posterior Density (HPD) of individual node ages are shown at selected branches. Letters (A-E) next to *Cervus elaphus* clades refer to intraspecific phylogeographic haplogroups. Calibration points were marked by yellow circles: 1 - the split of *Cervinae* into Cervini and Muntiacini based on previous molecular estimations (Świsłocka *et al.* 2020) and the oldest representative of Muntiacini (Dong 2007); 2 - the split of *Axis* and *Rucervus* based on the oldest fossils of *Axis* (Deng 2006; Vislobokova 2011); 3 - the divergence of *Rusa* and *Cervus* clade assumed according to the oldest *Rusa* (Vislobokova 2012); 4 - the separation of two *Cervus* clades: *C. elaphus* + *C. hanglu* and *C. canadensis* + *C. nippon* + *C. albirostris* according to the fossil *C. nestii* (Bellucci *et al.* 2014), probably belonging to the lineage of *C. elaphus* + *C. hanglu* (Croitor 2018; Croitor 2020).

Figure S5. Correspondence analysis plots obtained for the nucleotide composition of selected genes showing a similar composition in *C. hanglu* and the Eastern lineage deer. *Cervus* taxa: a - *C. albirostris*; c - *C. canadensis*; c_ala - *C. canadensis alashanicus*; c_kan - *C. canadensis kansuensis*; c_sib - *C. canadensis sibiricus*; c_son - *C. canadensis songaricus*; c_xan - *C. canadensis xanthopygus*; e_A - *C. elaphus* haplogroup A; e_B - *C. elaphus* haplogroup B; e_C - *C. elaphus* haplogroup C; e_D - *C. elaphus* haplogroup D; e_E - *C. elaphus* haplogroup E; e_mac - *C. elaphus macneilli*; h - *C. hanglu yarkandensis*; n_cen - *C. nippon centralis*; n_hor - *C. nippon hortulorum*; n_kop - *C. nippon kopschi*; n_sic - *C. nippon sichuanicus*; n_tai - *C. nippon taiouanus*; n_yak - *C. nippon yakushimae*; n_yes - *C. nippon yesoensis*; Anc - *Cervus* common ancestor .

C. albirostris

C. canadensis

C. elaphus

C. hanglu

C. nippon

Cervus ancestor

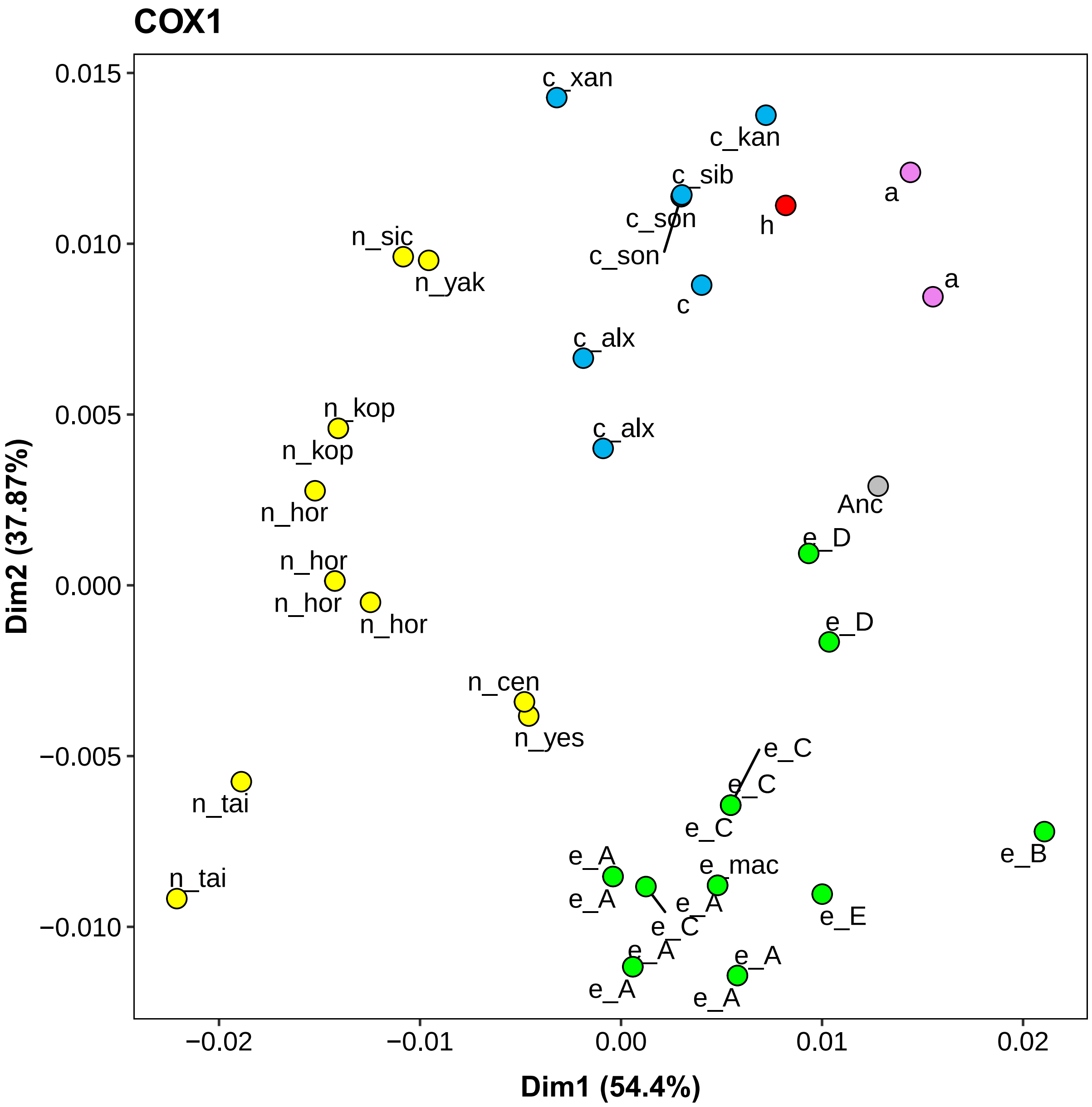
COX1
c_xan
c_kan
c_sib
a
h
c_son
c_son
n_sic
n_yak
c
a
c_alx
n_kop
n_kop
c_alx
n_hor
Anc
e_D
n_hor
n_hor
n_hor
e_D
n_cen
n_yes
e_C
e_C
n_tai
e_C
e_B
e_A
e_mac
n_tai
e_A
e_A
e_E
e_C
e_A
e_A
e_A
e_A
0.015
0.010
0.005
0.000
−0.005
−0.010
−0.02
−0.01
0.00
0.01
0.02
Dim1 (54.4%)
Dim2 (37.87%)

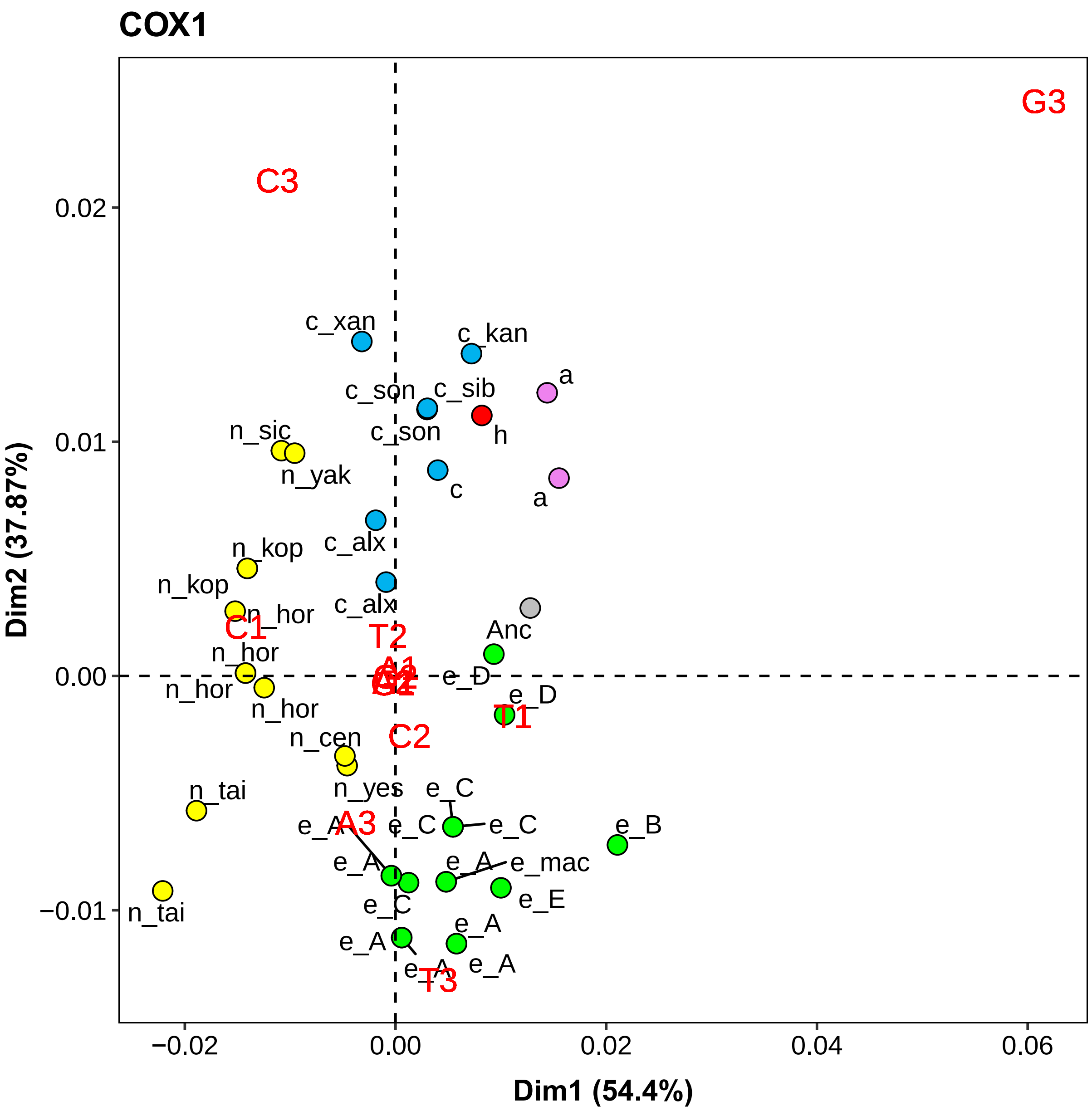

COX1
G3
C3
c_xan
c_kan
a
c_son
c_sib
h
n_sic
c_son
n_yak
c
a
c_alx
n_kop
n_kop
n_hor
c_alx
C1
T2
Anc
n_hor
n_hor
e_D
e_D
T1
n_hor
n_cen
C2
n_yes
n_tai
e_C
A3
e_A
e_C
e_C
e_B
e_A
e_A
e_mac
e_E
n_tai
e_C
e_A
e_A
e_A
e_A
T3
0.02
0.01
0.00
−0.01
−0.02
0.00
0.02
0.04
0.06
Dim1 (54.4%)
Dim2 (37.87%)

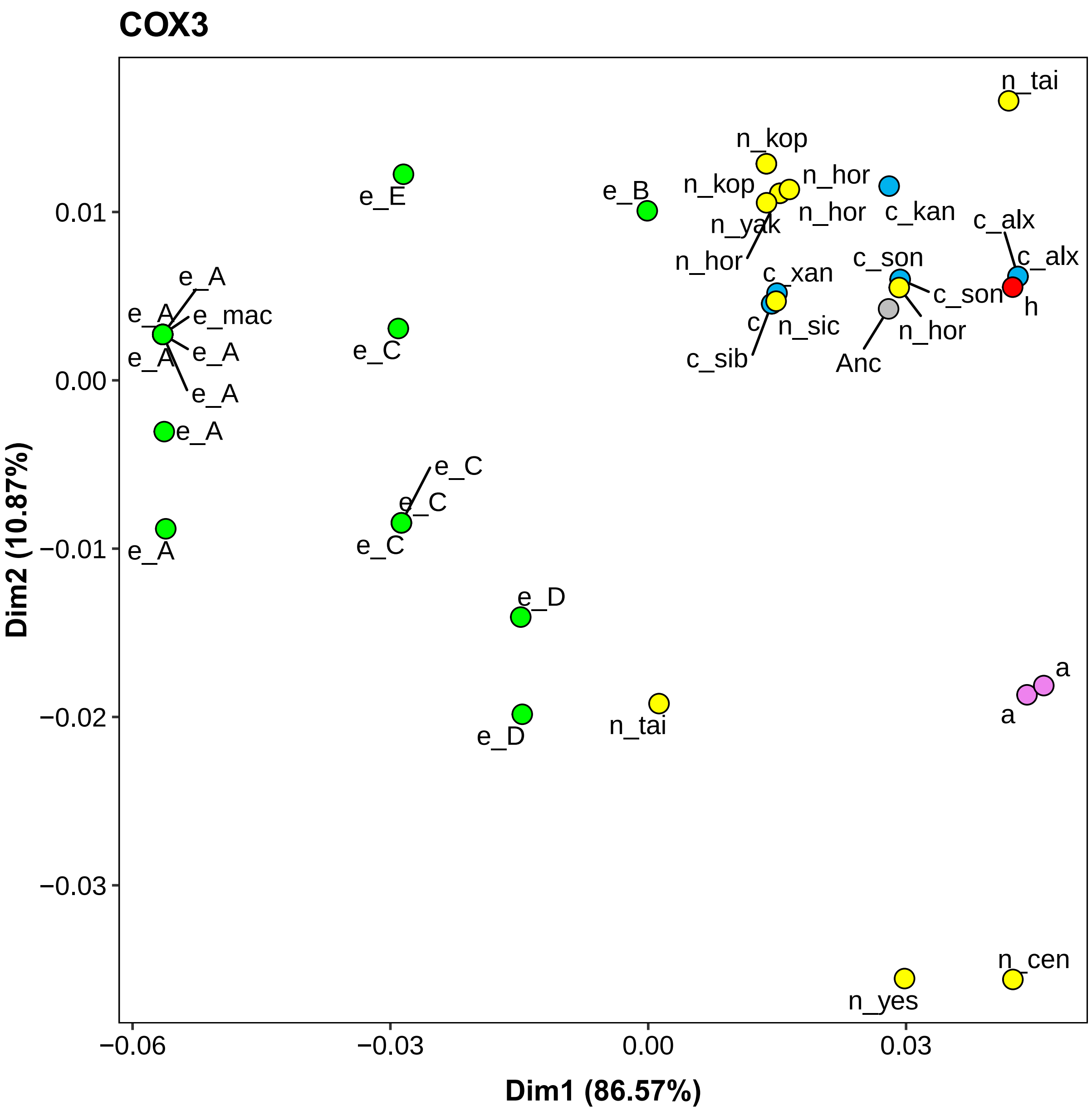

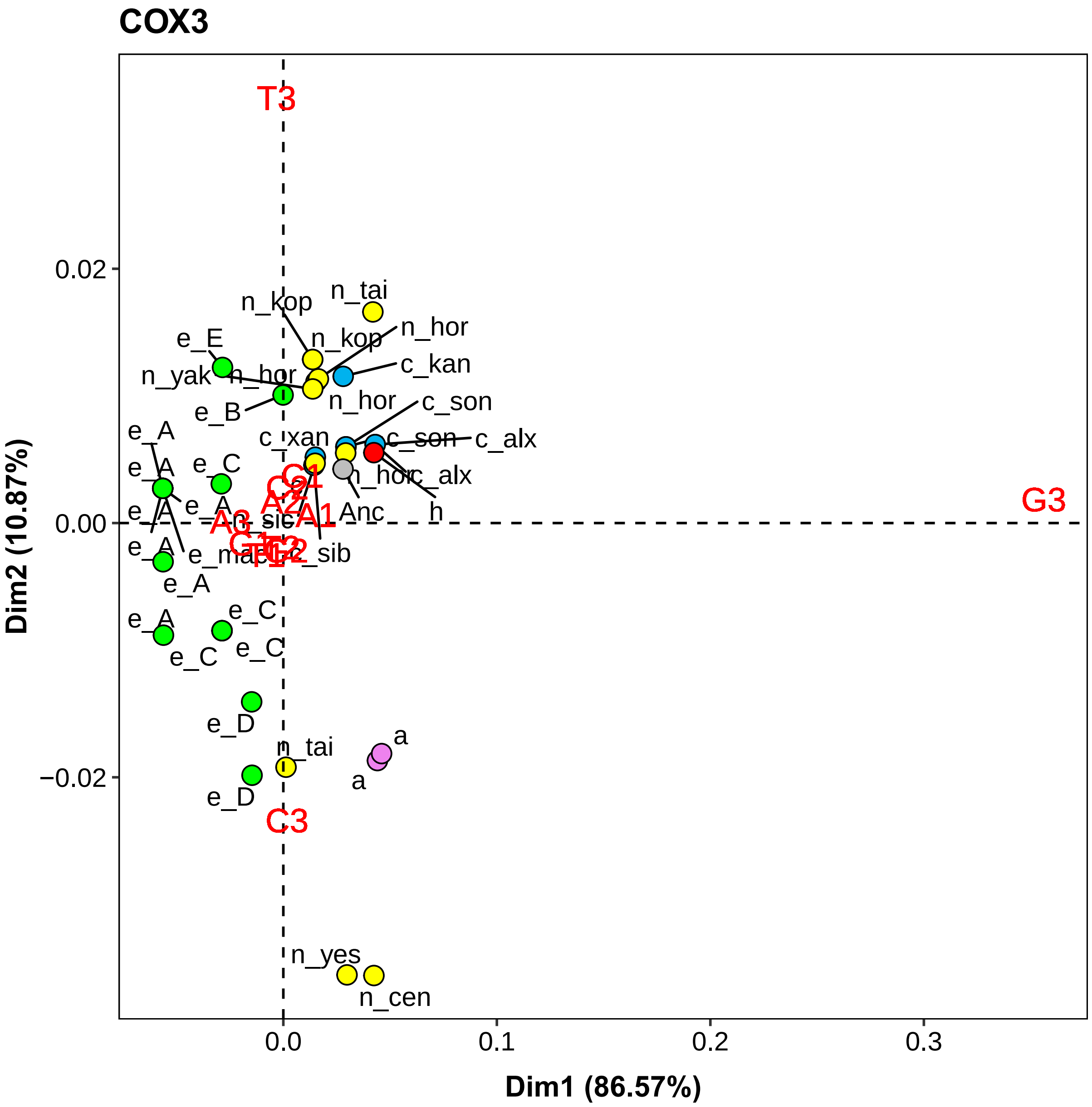
COX3
Dim1 (86.57%)
Dim2 (10.87%)
0.0
0.1
0.2
0.3
0.02
0.00
−0.02
T3
G3
C3
A1
A2
A3
C1
G1
C2
T1
n_tai
n_kop
n_hor
c_kan
e_E
n_yak
e_B
c_son
c_xan
c_alx
Anc
h
e_A
e_C
e_ma
n_sic
c_sib
e_D
a
n_yes
n_cen

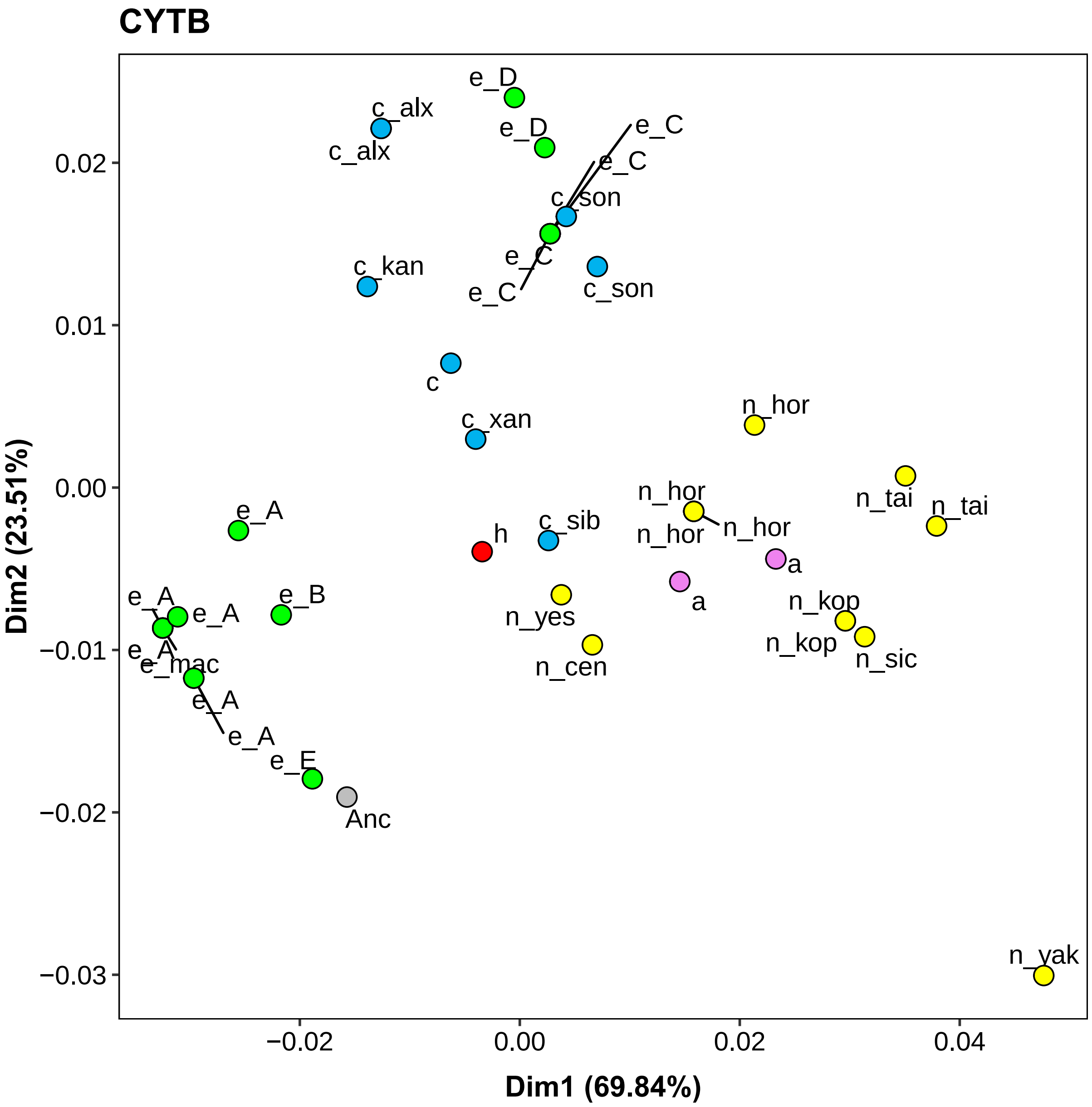
CYTB
Dim1 (69.84%)
Dim2 (23.51%)
−0.02
0.00
0.02
0.04
0.02
0.01
0.00
−0.01
−0.02
−0.03
e_D
e_D
c_alx
c_alx
e_C
e_C
e_C
e_C
c_son
c_son
c_kan
c
c_xan
n_hor
n_hor
n_hor
n_hor
n_tai
n_tai
e_A
h
c_sib
a
a
e_A
e_A
e_B
n_yes
n_kop
n_kop
n_sic
e_A
e_mac
n_cen
e_A
e_A
e_E
Anc
n_yak

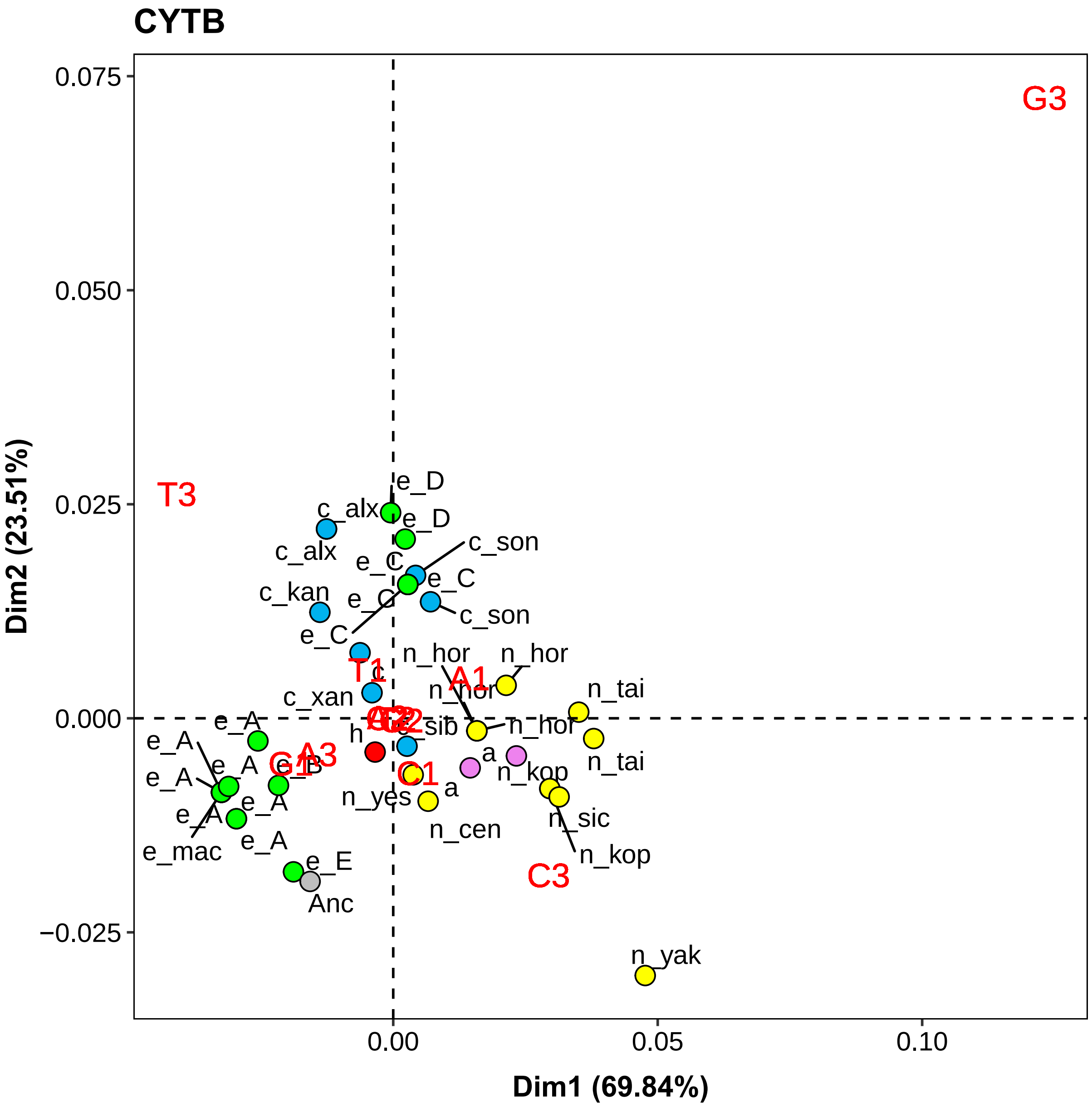
CYTB
Dim2 (23.51%)
Dim1 (69.84%)
0.075
0.050
0.025
0.000
−0.025
0.00
0.05
0.10
G3
T3
T1
A1
A3
G1
C1
C3
c_alx
c_alx
c_kan
c_son
c_son
c_xan
e_D
e_D
e_C
e_C
e_C
e_C
e_A
e_A
e_A
e_A
e_A
e_A
e_A
e_B
e_E
e_mac
Anc
h
n_hor
n_hor
n_hor
n_hor
n_tai
n_tai
n_kop
n_kop
n_sic
n_yes
n_cen
n_yak
a
a

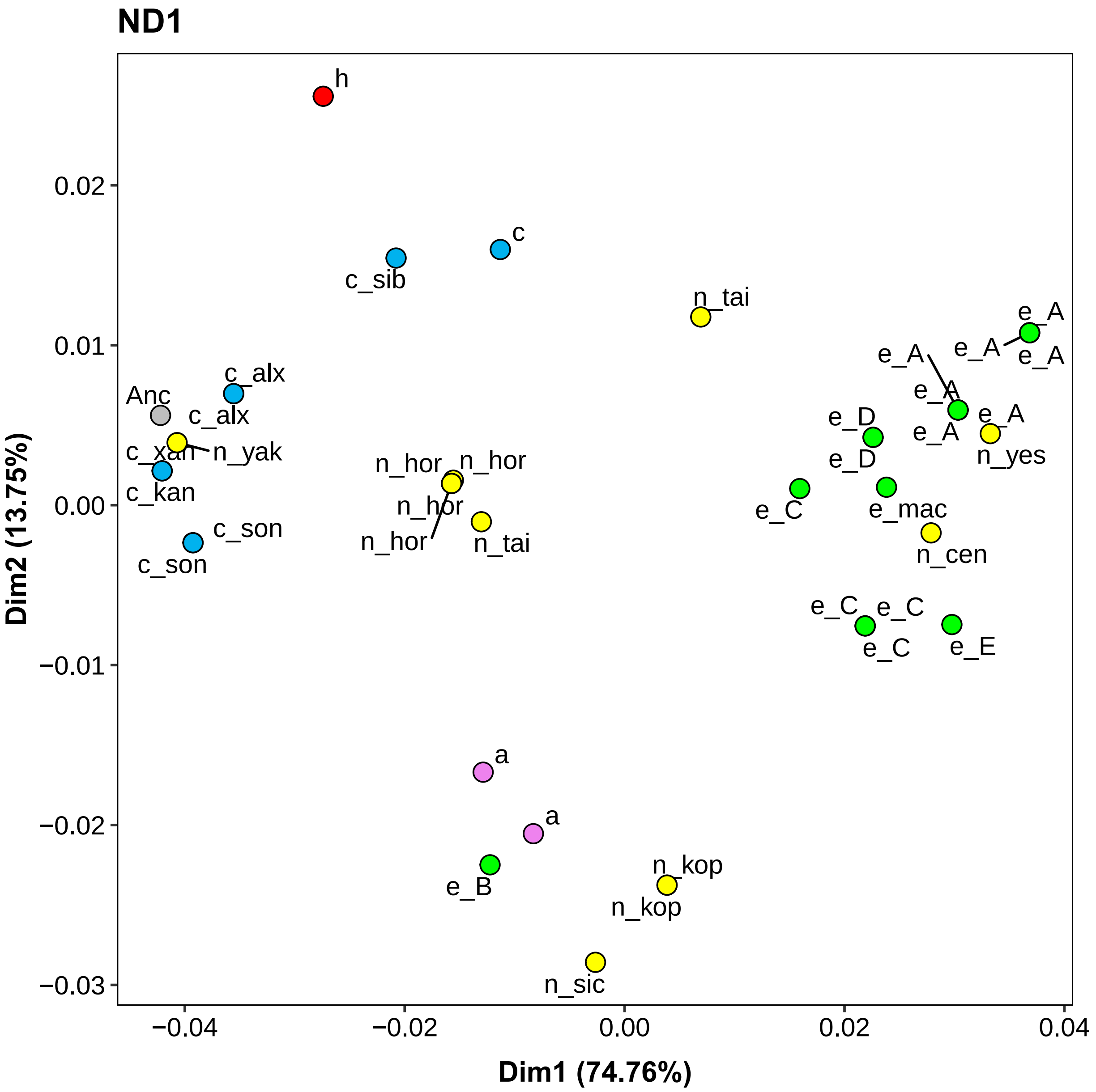
ND1
h
c
c_sib
n_tai
e_A
e_A
e_A
e_A
c_alx
Anc
c_alx
c_xan
n_yak
c_kan
e_A
e_A
e_A
e_D
n_yes
e_D
n_hor
n_hor
n_hor
n_hor
n_tai
e_C
e_mac
n_cen
c_son
c_son
e_C
e_C
e_C
e_E
a
a
e_B
n_kop
n_kop
n_sic
0.02
0.01
0.00
−0.01
−0.02
−0.03
−0.04
−0.02
0.00
0.02
0.04
Dim2 (13.75%)
Dim1 (74.76%)

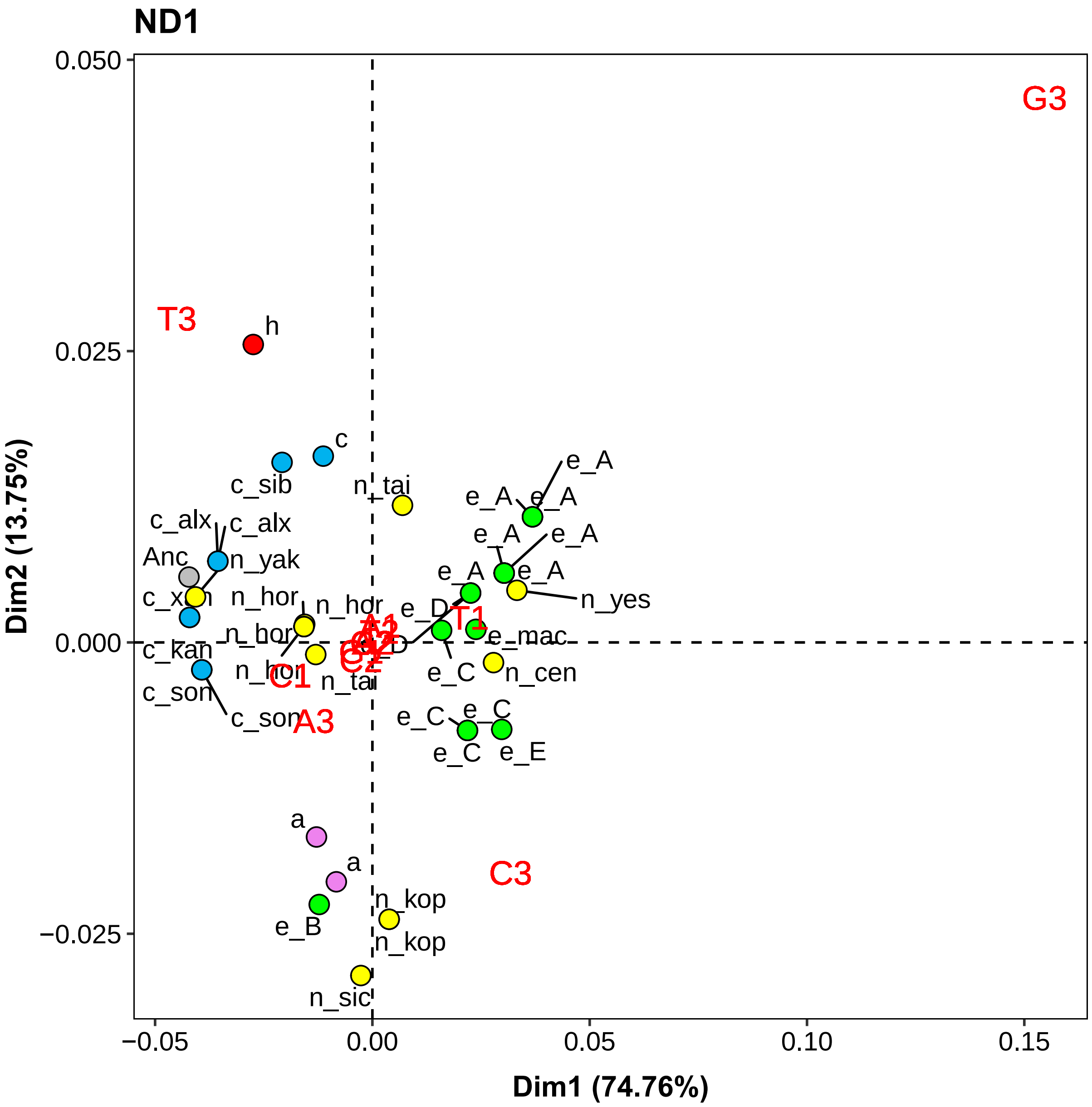

ND1
G3
T3
h
c
c_sib
n_tai
e_A
c_alx
c_alx
n_yak
Anc
c_xin
n_hor
n_hor
e_D
T1
n_yes
e_mac
A1
A2
C2
C1
n_cen
c_kan
c_son
c_son
A3
n_tai
e_C
e_C
e_E
a
a
C3
e_B
n_kop
n_kop
n_sic
Dim1 (74.76%)
Dim2 (13.75%)

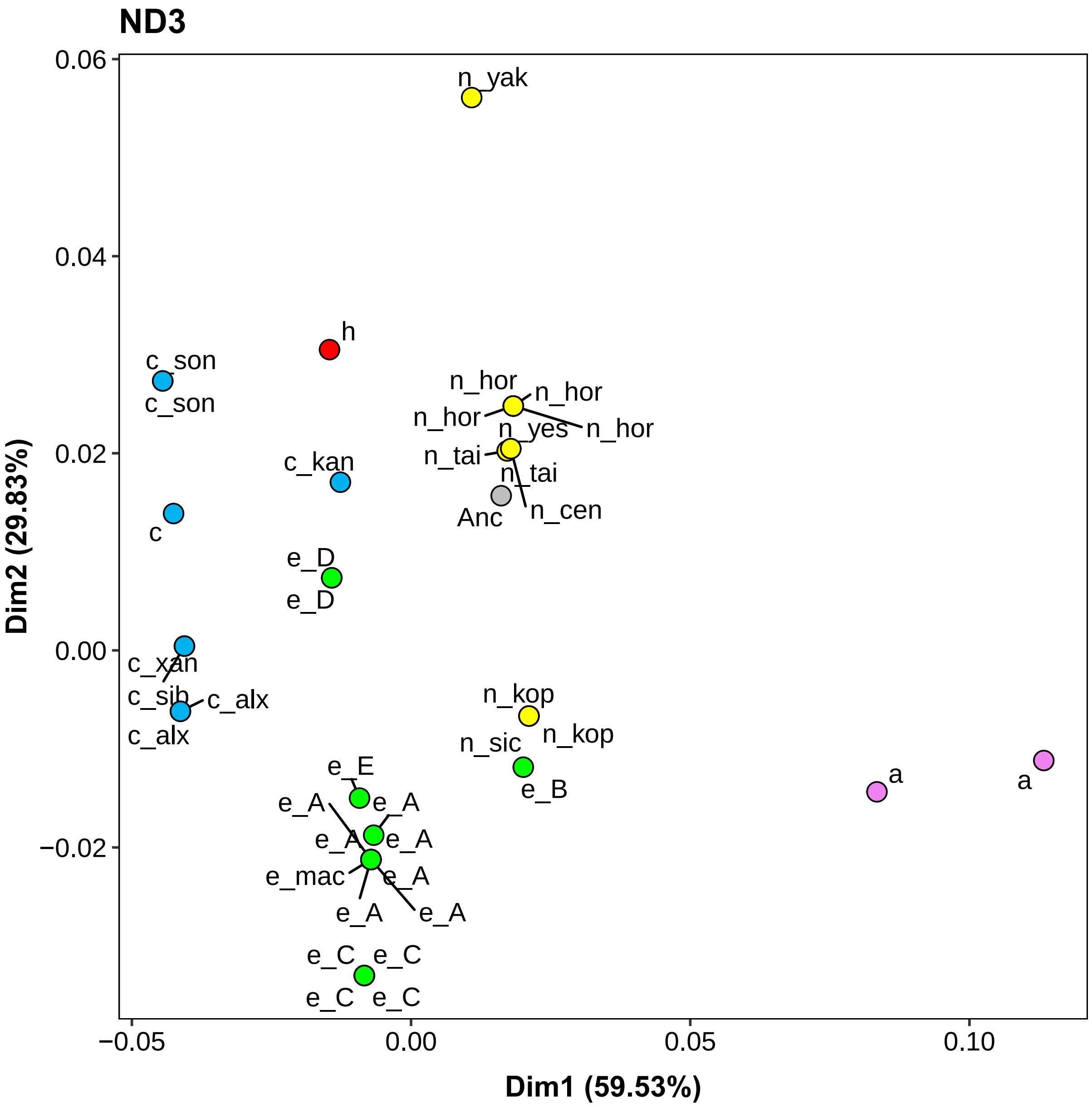

ND3
Dim1 (59.53%)
Dim2 (29.83%)
0.06
0.04
0.02
0.00
−0.02
−0.05
0.00
0.05
0.10
n_yak
h
c_son
c_son
n_hor
n_hor
n_hor
n_hor
n_yes
n_tai
n_tai
n_cen
Anc
c_kan
c
e_D
e_D
c_xan
c_sib
c_alx
c_alx
n_kop
n_kop
n_sic
e_B
a
a
e_E
e_A
e_A
e_A
e_A
e_mac
e_A
e_A
e_A
e_C
e_C
e_C
e_C

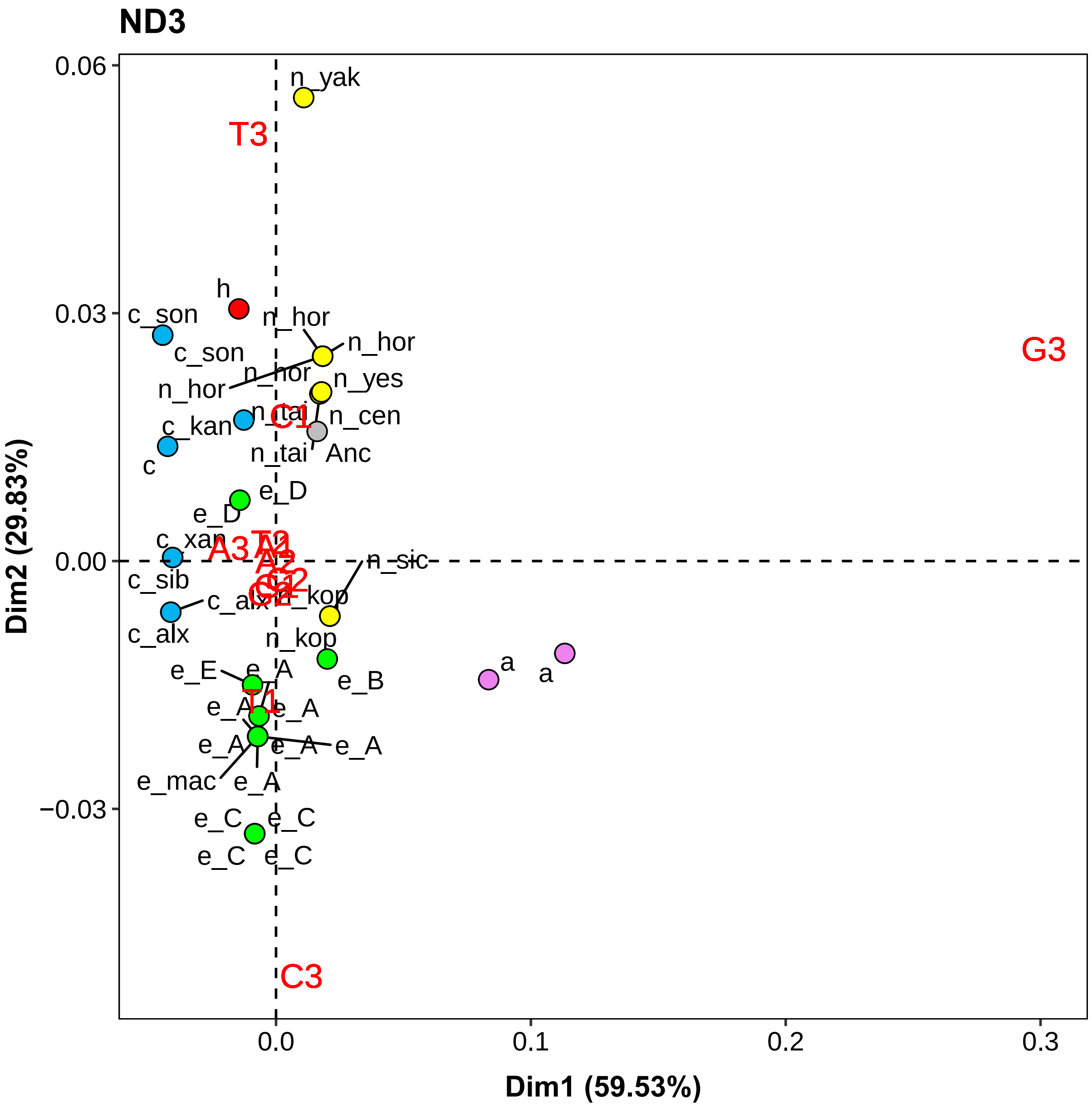
ND3
Dim1 (59.53%)
Dim2 (29.83%)
0.06
0.03
0.00
−0.03
0.0
0.1
0.2
0.3
n_yak
T3
h
c_son
n_hor
n_hor
c_son
G3
n_hor
n_hor
n_yes
n_tai
C1
c_kan
n_cen
c
n_tai
Anc
e_D
e_D
c_xan
A3
n_sic
c_sib
c_alx
n_kop
c_alx
n_kop
a
a
e_E
e_A
e_B
T1
e_A
e_A
e_A
e_A
e_A
e_mac
e_A
e_C
e_C
e_C
e_C
C3

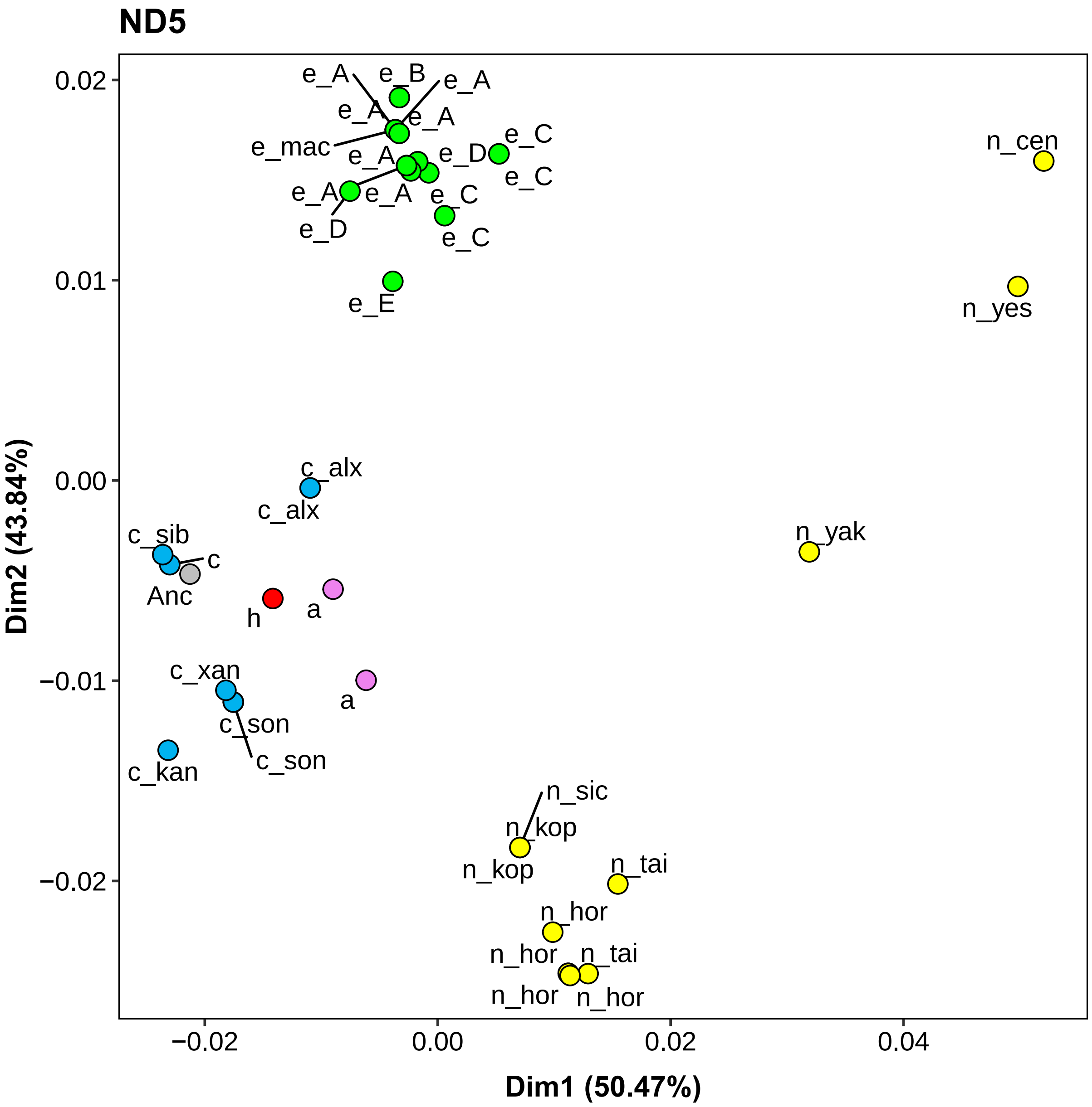
ND5
Dim1 (50.47%)
Dim2 (43.84%)
0.02
0.01
0.00
−0.01
−0.02
−0.02
0.00
0.02
0.04
e_A
e_B
e_A
e_A
e_A
e_mac
e_C
e_A
e_D
e_C
e_A
e_A
e_C
e_D
e_C
e_E
n_cen
n_yes
c_alx
c_alx
n_yak
c_sib
c
Anc
h
a
a
c_xan
c_son
c_son
c_kan
n_sic
n_kop
n_kop
n_tai
n_hor
n_hor
n_tai
n_hor
n_hor

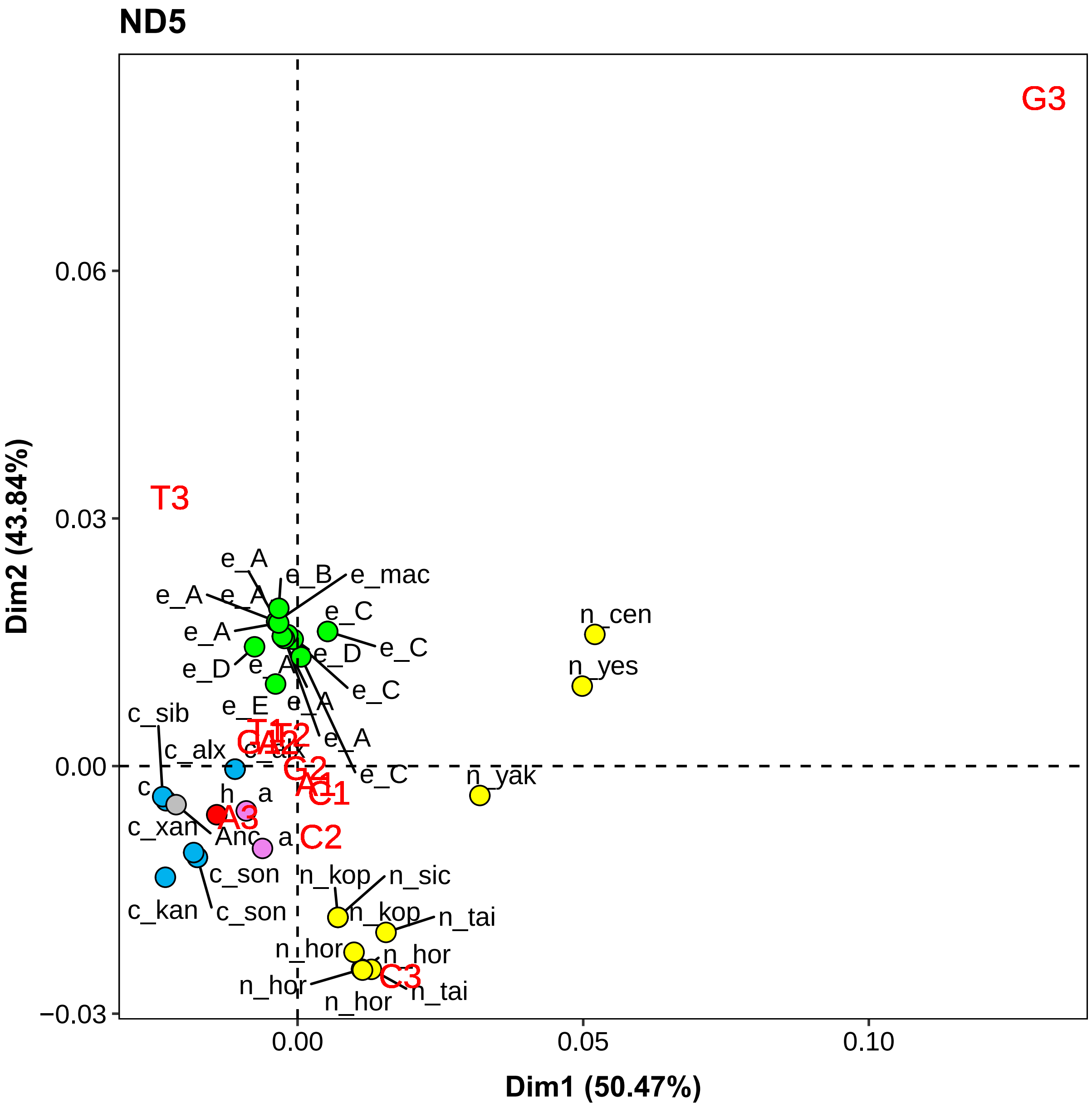
ND5
Dim1 (50.47%)
Dim2 (43.84%)
0.06
0.03
0.00
−0.03
0.00
0.05
0.10
G3
T3
C2
C3
C1
A3
e_A
e_B
e_mac
e_C
e_D
e_E
n_cen
n_yes
n_yak
n_kop
n_sic
n_tai
n_hor
c_sib
c_alx
c_xan
c_son
c_kan
Anc
h
a

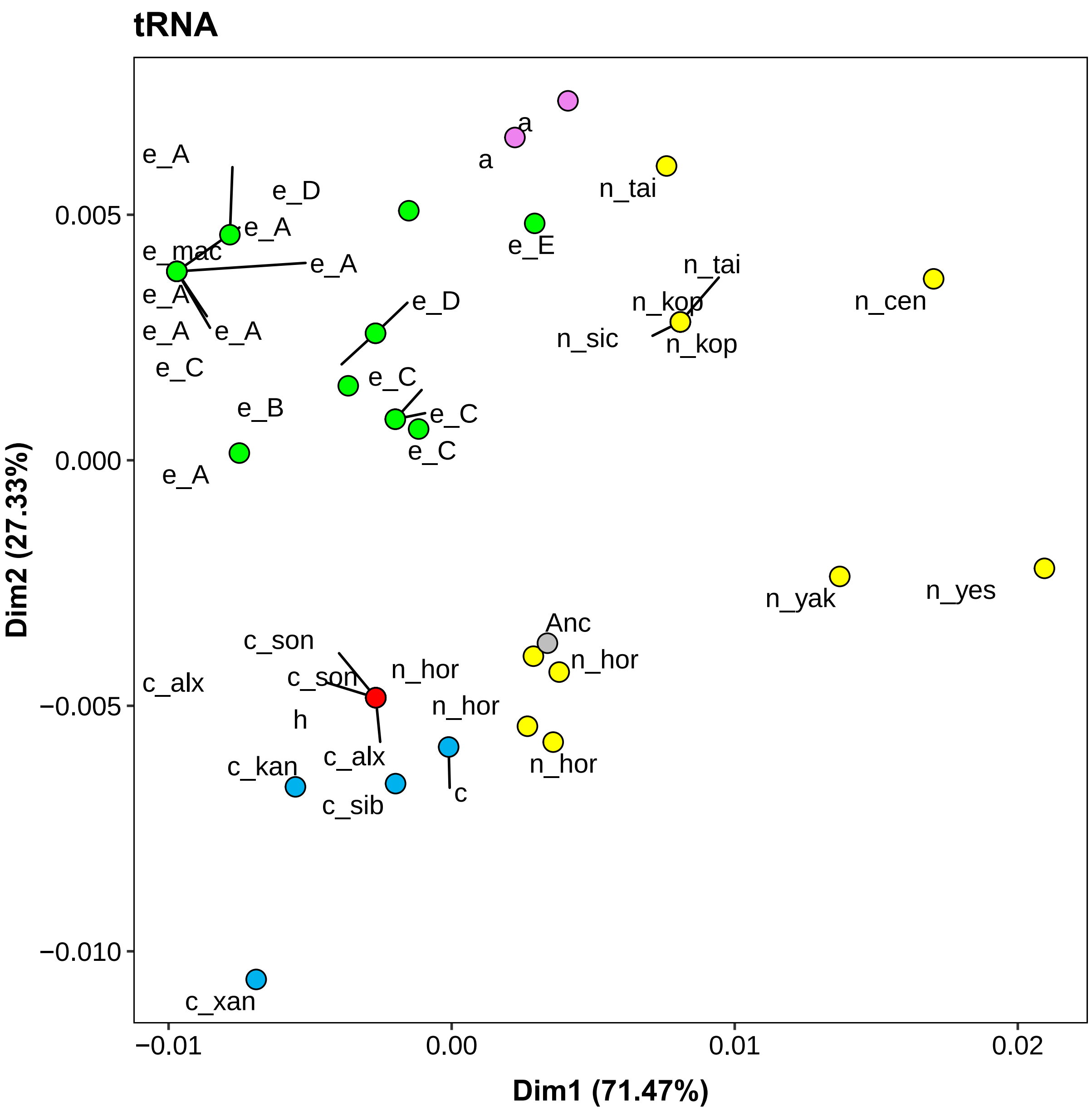
tRNA
Dim1 (71.47%)
Dim2 (27.33%)
−0.01
0.00
0.01
0.02
0.005
0.000
−0.005
−0.010
a
a
n_tai
e_A
e_D
e_A
e_mac
e_A
e_E
n_tai
n_cen
e_A
e_D
n_kop
e_A
e_A
n_sic
n_kop
e_C
e_C
e_B
e_C
e_C
e_A
n_yak
n_yes
Anc
c_son
n_hor
c_son
n_hor
c_alx
n_hor
h
c_alx
n_hor
c_kan
c_sib
c
c_xan

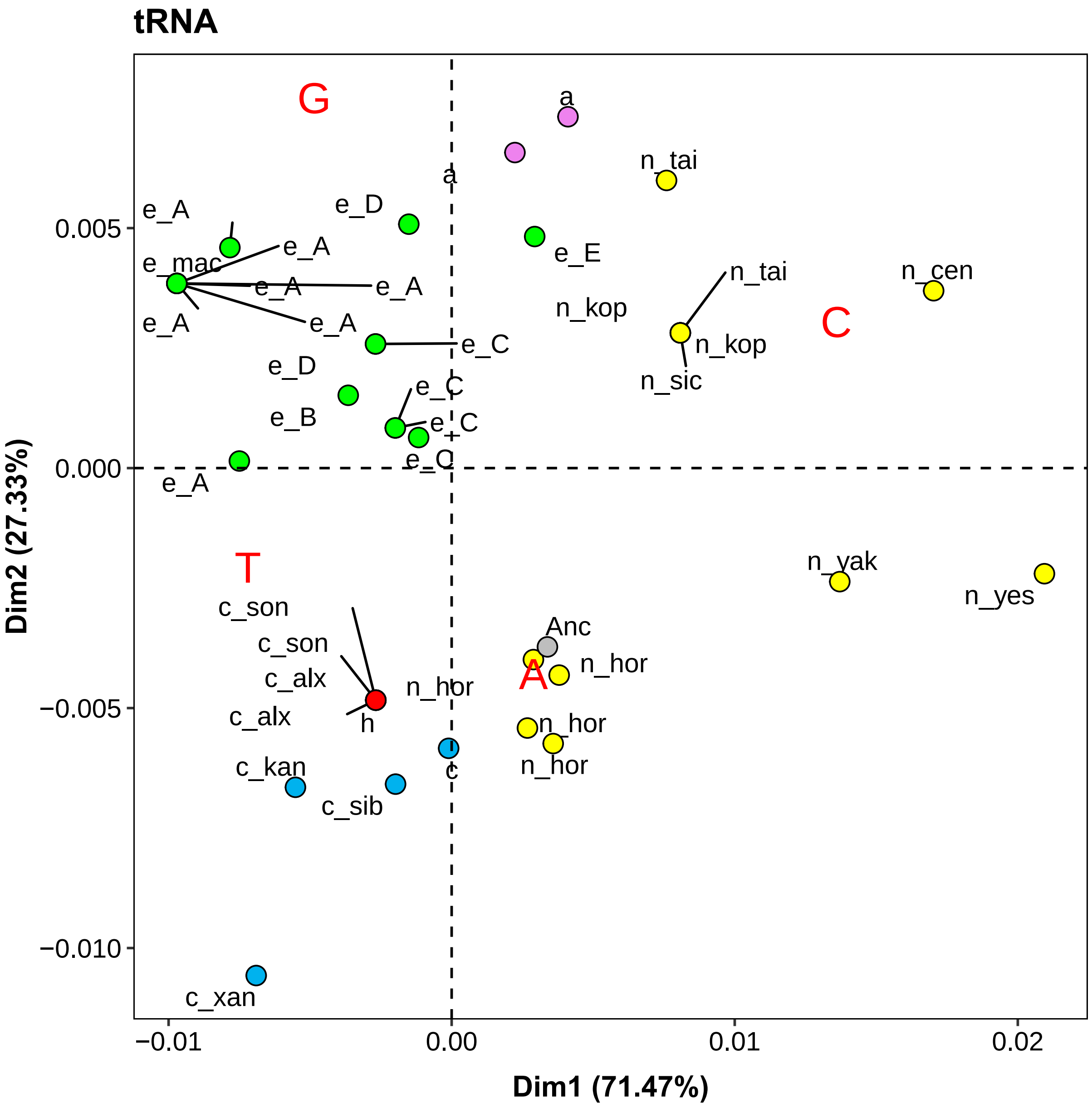
tRNA
G
T
A
C
a
a
n_tai
n_tai
e_A
e_D
e_E
e_A
e_mac
e_A
e_A
e_A
e_A
n_kop
n_kop
n_cen
e_C
e_D
n_sic
e_C
e_B
e_C
e_C
e_A
n_yak
n_yes
c_son
Anc
c_son
n_hor
c_alx
n_hor
c_alx
h
n_hor
c_kan
c
n_hor
c_sib
c_xan
0.005
0.000
−0.005
−0.010
−0.01
0.00
0.01
0.02
Dim2 (27.33%)
Dim1 (71.47%)

References

Bellucci, L, F Bona, P Corrado, D Magri, I Mazzini, F Parenti, G Scardia, R Sardella. 2014. Evidence of late Gelasian dispersal of African fauna at Coste San Giacomo (Anagni Basin, central Italy): Early Pleistocene environments and the background of early human occupation in Europe. *Quaternary Science Reviews* 96:72-85.

Chikuni K, Mori Y, Tabata T, Saito M, Monma M, Kosugiyama M. 1995. Molecular phylogeny based on the kappa-casein and cytochrome b sequences in the mammalian suborder Ruminantia. *Journal of Molecular Evolution* 41:859-866.

Croitor, R. 2018. *Plio-Pleistocene deer of Western Palearctic: Taxonomy, Systematics, Phylogeny*. Chișinău: Institute of Zoology of the Academy of Sciences of Moldova.

Croitor, R. 2020. A new form of wapiti *Cervus canadensis* Erxleben, 1777 (Cervidae, Mammalia) from the Late Pleistocene of France. *Palaeoworld* 29:789-806.

Deng, T. 2006. Chinese Neogene mammal biochronology. *Vertebrata PalAsiatica* 44:143–163.

Dong, W. 2007. New material of *Muntiacinae* (Artiodactyla, Mammalia) from the Late Miocene of the northeastern Qinghai-Tibetan Plateau, China. *Comptes Rendus Palevol* 6:335-343.

Fajardo V, Gonzalez I, Lopez-Calleja I, Martin I, Rojas M, Hernandez PE, Garcia T, Martin R. 2007. Identification of meats from red deer (*Cervus elaphus*), fallow deer (*Dama dama*), and roe deer (*Capreolus capreolus*) using polymerase chain reaction targeting specific sequences from the mitochondrial 12S rRNA gene. *Meat Science* 76:234-240.

Matosiuk M, Sheremetyeva IN, Sheremetyev IS, Saveljev AP, Borkowska A. 2014. Evolutionary neutrality of mtDNA introgression: evidence from complete mitogenome analysis in roe deer. *Journal of Evolutionary Biology* 27:2483-2494.

Świsłocka, M, M Matosiuk, M Ratkiewicz, A Borkowska, M Czajkowska, P Mackiewicz. 2020. Phylogeny and diversity of moose (*Alces alces*, Cervidae, Mammalia) revealed by complete mitochondrial genomes. *Hystrix-Italian Journal of Mammalogy* 31:1-9.

Vislobokova, IA. 2011. Historical development and geographical distribution of giant deer (Cervidae, Megacerini). *Paleontological Journal* 45:674-688.

Vislobokova, IA. 2012. Giant deer: Origin, evolution, role in the biosphere. *Paleontological Journal* 46:643-775.

Wood NJ, Phua SH. 1996. Variation in the control region sequence of the sheep mitochondrial genome. *Animal Genetics* 27:25-33.

Zachos J, Pagani M, Sloan L, Thomas E, Billups K. 2001. Trends, rhythms, and aberrations in global climate 65 Ma to present. *Science* 292:686-693.
